# Hierarchical quasiparticle dynamics in antiferromagnets revealed by time- and momentum-resolved X-ray scattering

**DOI:** 10.1038/s41467-026-77964-5

**Published:** 2026-09-29

**Authors:** Arnau Romaguera, Elizabeth Skoropata, Yun Yen, Biaolong Liu, Abhishek Nag, Shih-Wen Huang, Ludmila Leroy, Katja Sophia Moos, Gian Parusa, Serhane Zerdane, Ritwika Mandal, Céline Mariette, Matteo Levantino, Eugenio Paris, Luc Patthey, Ekaterina Pomjakushina, Urs Staub, Monica Ciomaga Hatnean, Michael Schüler, Elia Razzoli, Hiroki Ueda

**Affiliations:** 1 PSI Center for Photon Science, Paul Scherrer Institute, Villigen-PSI, Switzerland; 2 PSI Center for Scientific Computing, Theory and Data, Paul Scherrer Institute, Villigen-PSI, Switzerland; 3 École Polytechnique Fédérale de Lausanne (EPFL), Lausanne, Switzerland; 4 Institute for Theoretical Physics and Bremen Center for Computational Materials Science, University of Bremen, Bremen, Germany; 5 Department of Physics, University of Fribourg, Fribourg, Switzerland; 6 Institut des Matériaux de Nantes Jean Rouxel (IMN) – UMR 6502, Nantes Université, CNRS, Nantes, France; 7 ESRF – The European Synchrotron, 71 Avenue des Martyrs, CS40220, Grenoble, Cedex 38043 France; 8 PSI Center for Neutron and Muon Sciences, Paul Scherrer Institute, Villigen-PSI, Switzerland; 9 Materials Discovery Laboratory, Department of Materials, ETH Zürich, Zürich, Switzerland; 10 Present Address: ESRF- The European Synchrotron, 71 Avenue des Martyrs, CS40220, Grenoble, Cedex France; 11 Present Address: Department of Physics, Indian Institute of Technology, Roorkee, Uttarakhand India

**Keywords:** Magnetic properties and materials, Electronic properties and materials

## Abstract

Energy flows among coupled subsystems are essential for ultrafast dynamics and high-speed technologies. In magnetic materials, spin fluctuations —magnons— mediate these flows in ultrafast magnetism. Yet momentum-resolved access to low-energy magnons governing the microscopic dynamics has been lacking. Using time-resolved resonant diffuse scattering alongside complementary time-resolved X-ray techniques and quantum-kinetic simulations, we unveil the hierarchical energy pathways among correlated systems in the photoexcited antiferromagnet CuO. Above-bandgap excitation triggers near-instantaneous spin disorder, generating non-thermal magnons throughout reciprocal space within femtoseconds. Real-time momentum-resolved tracking reveals picosecond magnon quasi-thermalization, followed by nanosecond recovery via momentum-selective magnon-phonon scattering. The quasiparticle dispersion mismatch creates recovery bottlenecks that control non-equilibrium lifetimes. This microscopic framework transcends phenomenological models and generalizes across antiferromagnetic insulators, establishing design principles for ultrafast control of antiferromagnetic order.

## Abstract (web summary)

Using time-resolved scattering technique alongside complementary X-ray probes and simulations, Romaguera et al, reveal the hierarchical energy flow following photoexcitation in the antiferromagnet, CuO, from femtosecond demagnetization and magnon generation, through picosecond magnon quasi-thermalization, to nanosecond recovery.

## Introduction

Ultrafast manipulation of material states offers a powerful pathway to tailor material properties by driving systems into non-equilibrium states that are otherwise inaccessible^1–7^. Understanding the microscopic mechanisms underlying these ultrafast phenomena is essential for fundamental physics and next-generation high-speed technologies. Ultrafast magnetism has emerged as a particularly compelling research area, encompassing phenomena such as ultrafast demagnetization^8^, coherent magnon creation^9^, photo-induced phase transitions^1^, spin current generation^10^, all-optical magnetization switching^11^, and ultrafast modifications of spin fluctuations^12^, which can influence emergent phenomena, including superconductivity^13^, Peierls transitions^14^, and transport^15^. This breadth of phenomena highlights the significant potential for technological applications in spintronics, magnonics, and ultrafast magnetic storage, electronics, and logic devices.

Ultrafast magnetism typically involves complex interactions among multiple subsystems –spins, charges, orbitals, and the lattice– rather than occurring solely within the spin sector. While the multi-temperature model provides a qualitative framework for describing these coupled dynamics^8^, detailed microscopic insights require understanding the specific roles of other degrees of freedom, including phonons^16–18^, magnetic anisotropy^19^, and charge dynamics^20^. The intricate interactions following photoexcitation clarify leading mechanisms in fundamental magnetism, yet a comprehensive understanding demands tracking the dynamics of these interconnected subsystems and their hierarchical energy transfer pathways.

Here, we selectively investigate the transient behavior of the magnetic and lattice systems associated with ultrafast sublattice demagnetization in the antiferromagnetic (AFM) insulator cupric oxide (CuO). We combine three complementary time-resolved X-ray techniques, each with specific sensitivity to different degrees of freedom, enabling us to disentangle hierarchical interactions and track energy flow pathways. Together with microscopic quantum-kinetic simulations (photoexcited Hubbard model and quantum Boltzmann equation) and density-functional theory (DFT) calculations, these measurements establish a generalizable, comprehensive microscopic framework for the excitation and recovery processes of ultrafast sublattice demagnetization in AFM insulators.

**Fig. 1:**
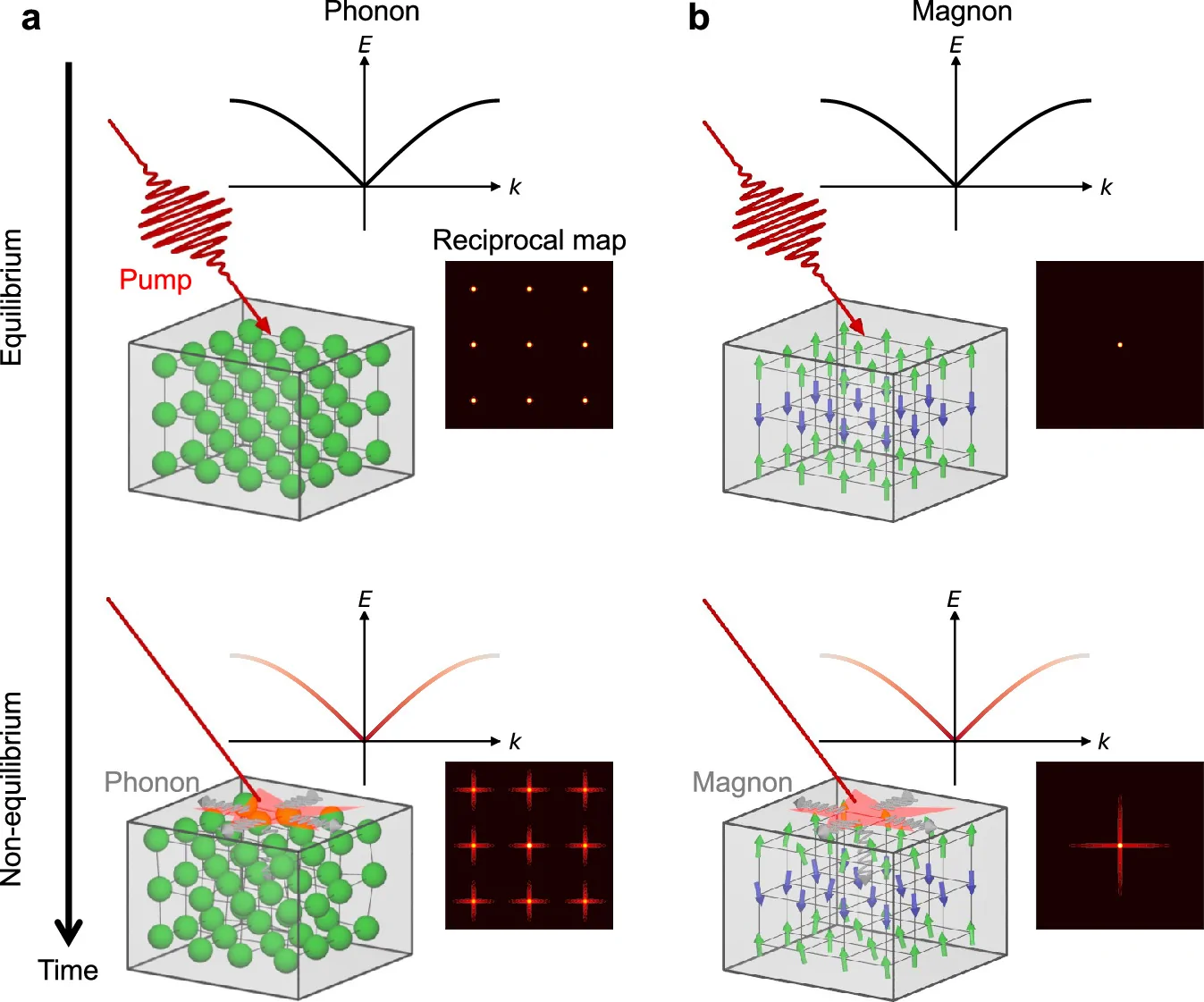
Schematic illustration of time-resolved diffuse scattering. **a** Time-resolved non-resonant diffuse scattering to probe phonons. **b** Time-resolved resonant diffuse scattering to probe magnons. Top: periodic arrangement of atoms or spins produces corresponding Bragg reflections in reciprocal space and low-energy collective excitations. Bottom: photoexcitation shakes the atomic positions or randomizes spin orientations, represented by the creation of low-energy quasiparticles along the dispersion curve, i.e., phonons and magnons, respectively. The enhanced density of low-energy quasiparticles results in diffuse scattering around Bragg reflections as streaks, enabling momentum-resolved tracking of collective excitation dynamics in non-equilibrium states.

Time-resolved *non-resonant* diffuse scattering (tr-NRDS) measures time- and momentum-dependent low-energy phonons, which appear as diffuse streaks around Bragg reflections^21,22^ (Fig. 1a) owing to the charge sensitivity of non-resonant X-ray scattering. In contrast, tuning the incident X-ray energy to an atomic resonance provides sensitivity to electronic degrees of freedom. Time-resolved resonant X-ray diffraction (tr-RXD) thus directly probes the time evolution of magnetic order^18,23,24^. In analogy, time-resolved *resonant* diffuse scattering (tr-RDS) is expected to access time- and momentum-resolved information on low-energy magnons (Fig. 1b), which would create diffuse streaks around magnetic Bragg reflections, though it has not yet been experimentally demonstrated. This emerging technique represents a powerful tool for revealing magnon dynamics in non-equilibrium states, providing unprecedented insights into ultrafast spin dynamics.

## Results

### Resonant diffuse scattering of non-equilibrium magnon origin

**Fig. 2:**
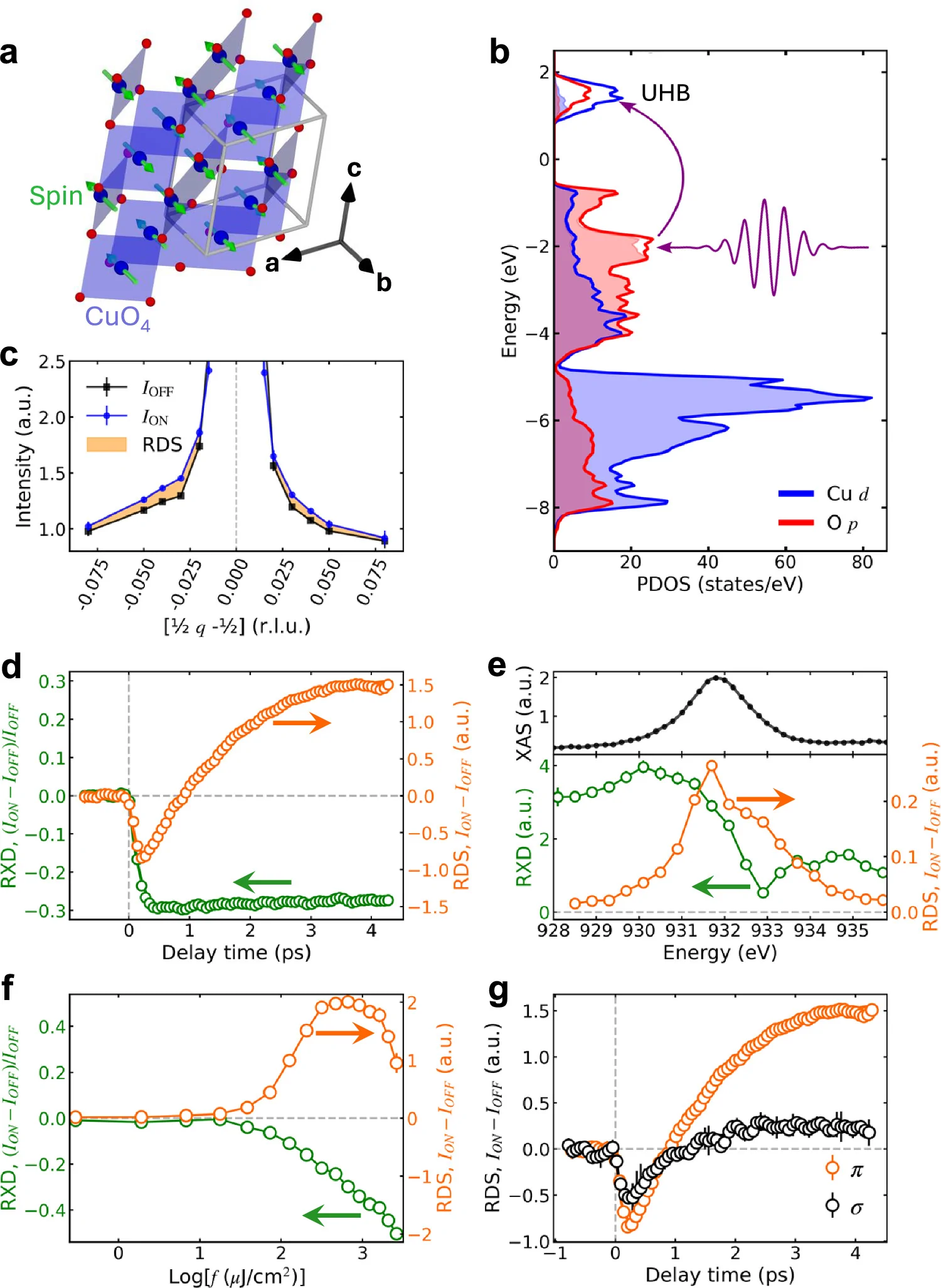
Resonant diffuse scattering from non-equilibrium magnons. **a** A magnetic structure of CuO in the collinear AFM phase. **b** Calculated projected density of states (PDOS) of CuO, showing the charge-transfer excitation by 400 nm laser pulses from O 2*p* band to Cu 3*d* upper Hubbard band (UHB). **c** Comparison of resonant X-ray scattering profiles around the (1/2 0 −1/2) RXD peak before (black) and 3 ps after photoexcitation (blue). The profiles were extracted from the tr-RDS data shown in Fig. 3a. The top of the diffraction peak is missing due to saturation of the detector. **d** Simultaneously collected time traces of the (1/2 0 −1/2) RXD intensity (green) and RDS intensity (orange). RDS intensity was collected at (0.49, 0.01, −0.51), which is the momentum allowing simultaneous collection with RXD intensity, unless otherwise stated. **e** X-ray absorption spectrum (XAS, black), and fixed-momentum incident X-ray energy dependences of the (1/2 0 −1/2) RXD intensity (green) and RDS intensity at (1/2, −0.08, −1/2) without intensity reduction due to the diffraction tail (orange). The large dip in RXD reflects the self-absorption effect^26^. **f** Laser fluence (*f*) dependence of the RXD (green) and RDS (orange) intensities measured at 4.5 ps after the photoexcitation. **g** Incident X-ray polarization dependence of RDS time traces for *π* (orange) and *σ* (black) polarizations. The fast intensity reduction observed in (**d**, **g**) is due to the RXD tail contribution (see main text). Error bars represent standard error. All the measurements were performed at 190 K. The pump laser fluence for the data shown in (**c**–**e**, **g**) was 3 mJ cm^−2^.

Monoclinic CuO (space group *C*2/*c*^25^) exhibits collinear AFM order (Fig. 2a), manifested by the (1/2 0 −1/2) magnetic Bragg reflection at the Cu *L*_3_ edge^26^. The electronic structure indicates that CuO is a charge-transfer insulator^27^, as consistent with our DFT calculations (Fig. S19). A femtosecond laser pulse with a wavelength of 400 nm photo-dopes electrons into the upper Hubbard band of Cu 3*d* orbitals via charge transfer excitation from O 2*p* orbitals (Fig. 2b). The resultant doublons (doubly occupied electrons on Cu sites) quench exchange interactions via spin-charge coupling^28,29^ (Supplementary Note 1) and trigger ultrafast sublattice demagnetization on a femtosecond timescale set by the electron hopping during the pump duration. Indeed, the magnetic Bragg reflection intensity quasi-instantaneously decreases in a step-function-like manner within ~70 fs (Fig. 2d), consistent with the previous observation with an 800 nm excitation^23^. Typical doublon lifetimes in copper-based oxides are within ~200 fs^30,31^. Note that this ultrafast sublattice demagnetization process, driven by the intrinsic spin-charge coupling in Mott insulators, differs fundamentally from phonon-mediated mechanisms in itinerant ferromagnets^32,33^.

Remarkably, the resonant X-ray scattering intensity measured simultaneously at (0.49, 0.01, −0.51), slightly detuned from the Bragg point (see “Methods” and Fig. S15), shows a substantial upturn signal above the baseline after an initial fast decrease (Fig. 2d), which reflects the magnetic Bragg reflection tail. Fitting to two exponential functions (Supplementary Note 2) reveals a distinct upturn component with a much longer time constant than the RXD reduction. This upturn signal exhibits clear resonant enhancement at the Cu *L*_3_ edge (Fig. 2e). Note that the spectral difference from RXD signals exhibiting a dip structure due to strong self-absorption^26^ likely originates from a larger magnon density created by the photoexcitation, as discussed later, near the surface with a depth profile, limiting the self-absorption effect. Pump fluence dependence at a fixed delay time after photoexcitation (4.5 ps) demonstrates that the upturn signal arises only above a certain fluence where ultrafast sublattice demagnetization occurs (Fig. 2f). The upturn signal decreases at high fluence because the RXD intensity reduction is larger and nonlinear at temperatures closer to the AFM transition temperature (Supplementary Note 3 for temperature estimation). Crucially, the incident X-ray polarization dependence shows significantly larger upturn signals for *π* polarization than *σ* polarization (Fig. 2g). This polarization dependence is similar to the magnetic RXD profile (Fig. S18) and reveals a magnetic origin of the upturn signals rather than an isotropic charge origin. Furthermore, the (1/2 0 −1/2) RXD peak width remains nearly constant while the tail intensity rises (Fig. S4), excluding the possibility of a change in the correlation length of the long-range order^22^ as the origin of the observed dynamics. This observation also indicates that our excitation does not quench the AFM order parameter. Therefore, the recovery process is not described by the Kibble-Zurek mechanism and subsequent coarsening of small domains^34^. A photo-induced phase transition into the incommensurate phase^23^ is inconsistent with our observation (Supplementary Note 4).

As established for resonant inelastic X-ray scattering (RIXS), the inelastic part of the resonant X-ray scattering cross-section contains information on the dynamical charge susceptibility and the dynamical spin-spin correlation function^35^. The phase factor around the half-integer momentum point suppresses the dynamical charge susceptibility and low-energy phonon contributions. In contrast to time-resolved RIXS (tr-RIXS), tr-RDS measures the time (*t*)-dependent, energy-integrated spin-spin correlation^36^. As detailed in the Supplementary Note 5, the RDS intensity follows:

1 $${I}_{{{\rm{RDS}}}}({{\bf{q}}},t)\propto \mathop{\sum}\limits_{\alpha {\alpha }^{{\prime} }}{[{\hat{\epsilon }}_{i}\times {\hat{\epsilon }}_{o}]}_{\alpha }^{*}{[{\hat{\epsilon }}_{i}\times {\hat{\epsilon }}_{o}]}_{{\alpha }^{{\prime} }}{S}_{\alpha {\alpha }^{{\prime} }}({{\bf{q}}},t)\,,$$

where ${\hat{\epsilon }}_{i}$ (${\hat{\epsilon }}_{o}$) denotes the incoming (outgoing) polarization; ${S}_{\alpha {\alpha }^{{\prime} }}({{\bf{q}}},t)$ is the momentum (**q**)-dependent spin-spin correlation function for each time snapshot; *α* and ${\alpha }^{{\prime} }$ denote the Cartesian coordinate directions of the lab frame. In leading order, the correlation function is determined by the magnon distribution *f*_*μ*_(**q**, *t*)^37^:

2 $${S}_{\alpha {\alpha }^{{\prime} }}({{\bf{q}}},t)\propto \mathop{\sum}\limits_{\mu }\left[{A}_{\mu }({{\bf{q}}}){f}_{\mu }({{\bf{q}}},t)+{\bar{A}}_{\mu }({{\bf{q}}}){\bar{f}}_{\mu }({{\bf{q}}},t)\right]\,,$$

where *A*_*μ*_(**q**) and ${\bar{A}}_{\mu }({{\bf{q}}})$ are prefactors specific to the magnetic ground state (see “Methods”) and ${\bar{f}}_{\mu }={f}_{\mu }+1$. Hence, tr-RDS allows us to directly access the time- and momentum-dependent magnon distribution triggered by the ultrafast spin disorder, apart from geometric factors.

**Fig. 3:**
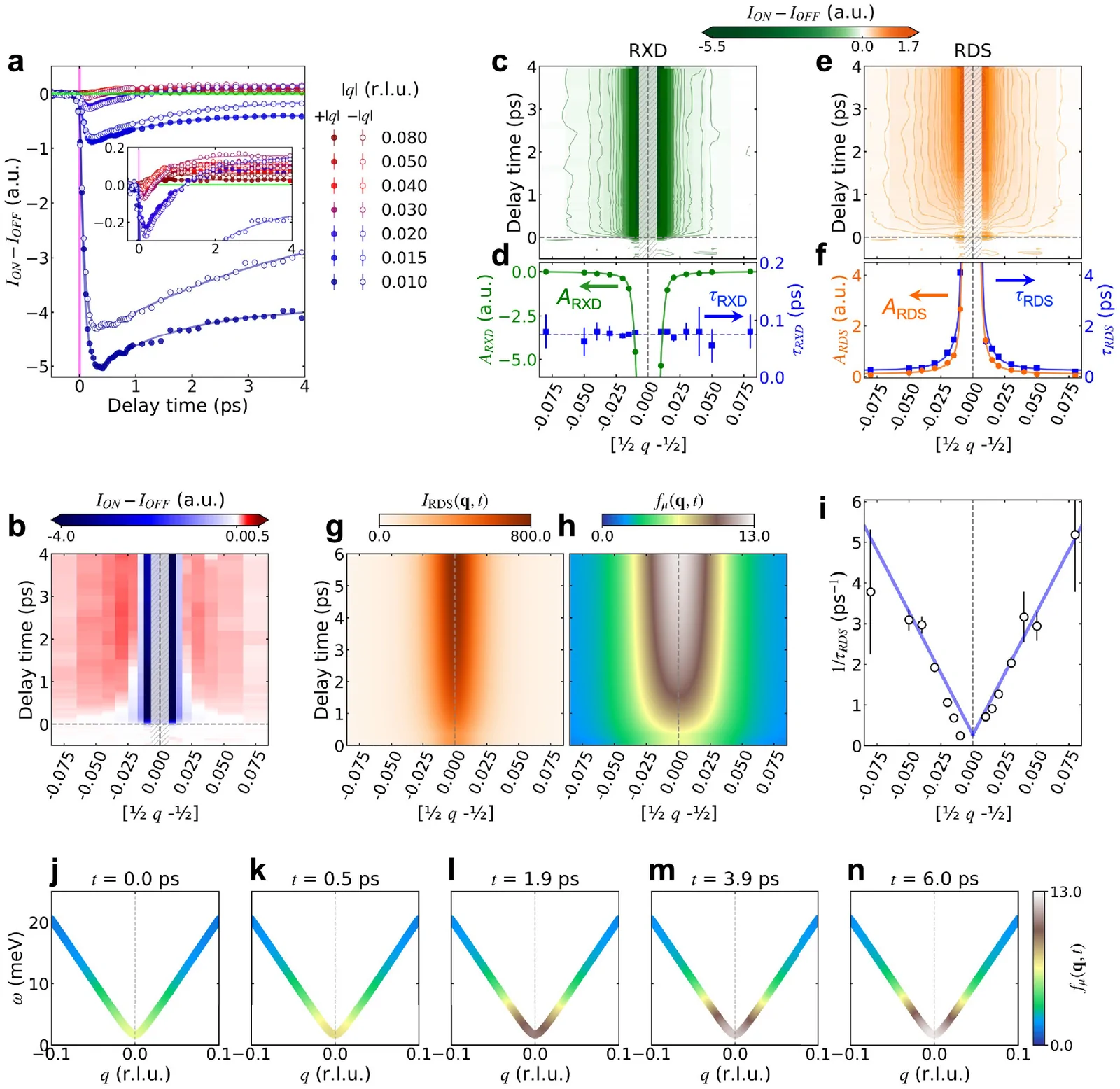
Momentum dependence of time-resolved resonant diffuse scattering. **a** Time traces of resonant X-ray scattering intensities at momentum points around **q**_RXD_ (see text) along b^*^ collected with the pump laser fluence of 3 mJ cm^−2^. Solid curves represent fits using two exponential functions to separate the RXD reduction and RDS upturn components (Supplementary Note 2). Filled (open) circles indicate data measured at positive (negative) momentum deviation *q* from **q**_RXD_. The inset shows a zoom-in of the upturn signals. The slight deviation from the symmetric behavior, represented by systematically larger signals in the negative *q* range than the positive *q* range, is due to a small error in the definition of the origin (*q* = 0). **b** Momentum-delay time contour plots of the experimental data shown in (**a**). **c**,** e** Momentum-delay time contour plots of the RXD (**c**) and RDS (**e**) components derived from the time traces in **a** by decomposing into the two components. **d**,** f** Momentum dependence of RXD-reduction (**d**) and RDS-upturn (**f**) amplitudes (*A*) and their time constants (*τ*) extracted from fits to the data in (**a**), demonstrating the symmetric amplitude profile expected from the symmetry and magnon redistribution, as shown by the green, blue, and orange solid curves representing the Gaussian-dominated pseudo-Voigt fits (guides to the eye). Error bars represent standard error in **a** and are from the fit in (**d**, **f**). The hatched area represents the momentum range where strong RXD signals prevented us from measuring resonant X-ray scattering intensities due to saturation. **g** Simulated RDS time traces and **h** time-dependent magnon distribution along [1/2 *q *−1/2]. **i** The scattering rate 1/*τ*_RDS_ as a function of momentum along [1/2 *q *−1/2]. Solid lines are guides to the eye. **j–n** Magnon density distribution along [1/2 *q *−1/2] at **j** *t* = 0 ps, **k** 0.5 ps, **l** 1.9 ps, **m** 3.9 ps and **n** 6.0 ps, derived from tr-RDS data using Eq. (2).

The magnon dispersion, defined by exchange interactions, follows the magnetic structure symmetry (magnetic point group $2/m.{1}^{{\prime} }$), and therefore the RDS signals of magnon origin should also respect the symmetry. Figure 3 shows the momentum dependence of the RDS intensities around the (1/2 0 −1/2) reflection (hereafter, referred to as **q**_RXD_) along b^*^. Although the resonant X-ray scattering signals at the momentum points close to **q**_RXD_ have a sizable quasi-instantaneous intensity reduction attributed to the RXD tail, the signals also show a substantial upturn at these momentum points at later timescales (Fig. 3a). Fitting with two exponential functions successfully separates the RDS signals from the RXD signals (Figs. 3c, e and Fig. S3). While the RXD signal amplitude increases with proximity to **q**_RXD_, its time constant remains constant (Fig. 3d), indicating a global suppression of the AFM order, i.e., ultrafast sublattice demagnetization. The amplitude of RDS signals is symmetric with respect to **q**_RXD_ along b^*^ [Fig. 3f (orange)], as expected from the magnon dispersion in a centrosymmetric structure. Compared to RDS signals along b^*^, the amplitude of RDS signals along [*h*0*h*] significantly differs (Supplementary Note 6 and Fig. S7), indicating strong anisotropy in magnon dispersion as expected from the symmetry, while RXD signals are nearly identical between the two directions.

### Quasi-thermalization after photoexcitation

Unlike the RXD time traces, the time constant of RDS signals [Fig. 3f (blue)] strongly depends on momentum. This indicates a time-dependent redistribution of the magnon density in reciprocal space along the dispersion curve. Given the time scale of a few picoseconds, the likely primary mechanism behind the observed magnon redistribution is magnon-magnon scattering. Magnon-electron scattering can be excluded since the electrons are already thermalized within femtosecond timescales^30,31^; magnon-phonon scattering occurs at later timescales (discussed in the next section). Considering the significant difference in spin gap energies between the acoustic (~1.3 meV) and optical (~23.2 meV) magnon branches^38,39^, the observed tr-RDS signals around the Γ point dominantly originate from the acoustic magnons and their quasi-thermalization via magnon-magnon scattering. Thus, tr-RDS complements tr-RIXS, as the latter accesses only high-energy magnetic excitation due to limited intrinsic energy resolution^12,40^.

To theoretically investigate the magnon dynamics, we employ a quantum-kinetic approach based on a spin-charge-coupled model derived from the Hubbard model^41^ (Supplementary Notes 1 and 7). The photoexcitation is modeled as quasi-instantaneous creation of doublons, creating non-thermal magnons. With this initial state as input, we solve the quantum Boltzmann equation for magnons,

3 $$\begin{array}{r}\frac{d}{dt}{f}_{\mu }({{\bf{q}}},t)={S}_{\mu }[\{f\}]({{\bf{q}}},t)\,,\end{array}$$

where *S*_*μ*_[{*f*}](**q**, *t*) is the magnon-magnon scattering integral (similar to electron-electron scattering) derived from the spin Hamiltonian in leading order beyond linear spin-wave theory. Our calculations are performed for a simplified orthorhombic unit cell (see “Methods”) to reduce computational costs while faithfully reproducing the low-energy magnon dynamics. Combining the calculated time-dependent magnon distribution *f*_*μ*_(**q**, *t*) with Eqs. (1, 2) enable us to simulate the tr-RDS signals.

Our simulations reveal that the magnons created via the spin-charge coupling are initially distributed relatively broadly throughout the Brillouin zone, redistribute energy through the magnon-magnon scattering, and then accumulate around the Γ point (Figs. 3j–n and S8), where the acoustic magnon energy is minimum with a spin gap^38,39^. Note that the quasi-instantaneous spin-charge coupling is only weakly momentum dependent, and thus the initial magnon distribution is non-thermal (Fig. S2), leading to momentum-dependent hot magnon temperatures, similar to previous studies on a ferromagnet^33^. The scattering rate (1/*τ*_RDS_) is proportional to momentum (Fig. 3i). Hydrodynamic magnon slowdown via magnon-magnon scattering was reported in antiferromagnets^42^, and our simulations also show the slowdown (Fig. S9).

Our tr-RDS signals exhibit self-similarity, as shown in Fig. S10a. Far-from-equilibrium universality in antiferromagnets was theoretically discussed for general two-dimensional Heisenberg Hamiltonians^43^. By using the same scaling exponents as in ref. ^43^, we obtained a power-law exponent of 2.28 ± 0.08 (see Fig. S10b), reasonably close to the nominal value (2.33) for an inverse particle cascade, in which a conserved number of particles flows to lower momentum in reciprocal space, consistent with and further validating our microscopic view of magnon dynamics (see also Supplementary Note 8). Differences in dimensionality and detailed Hamiltonian, and a possible non-conserving scattering process, as well as a finite pump pulse duration and doublon lifetime, may account for the small discrepancy in the small *t*^*β*^*q* range.

The calculated tr-RDS profile based on the time evolution of magnon density distribution (Fig. 3h) reproduces the momentum-delay time plots of the upturn component of the resonant X-ray scattering intensities (compare Fig. 3e, g). The magnon-magnon scattering changes the magnon distribution while keeping the total magnon energy constant and reaches a quasi-thermalized state within the branch fulfilling Bose-Einstein statistics. While similar magnon redistribution behavior was previously implied in Mott insulators using tr-RIXS without detailed time-momentum mapping^40^, our tr-RDS measurements together with the fully quantum-mechanical calculations provide the first direct visualization of this microscopic quasi-thermalization process in reciprocal space. Energy integration in tr-RDS enables momentum-space mapping with higher photon counts than energy-resolved tr-RIXS, facilitating direct visualization of real-time momentum-resolved quasi-particle dynamics.

### Magnetic order recovery via magnon-phonon scattering

**Fig. 4:**
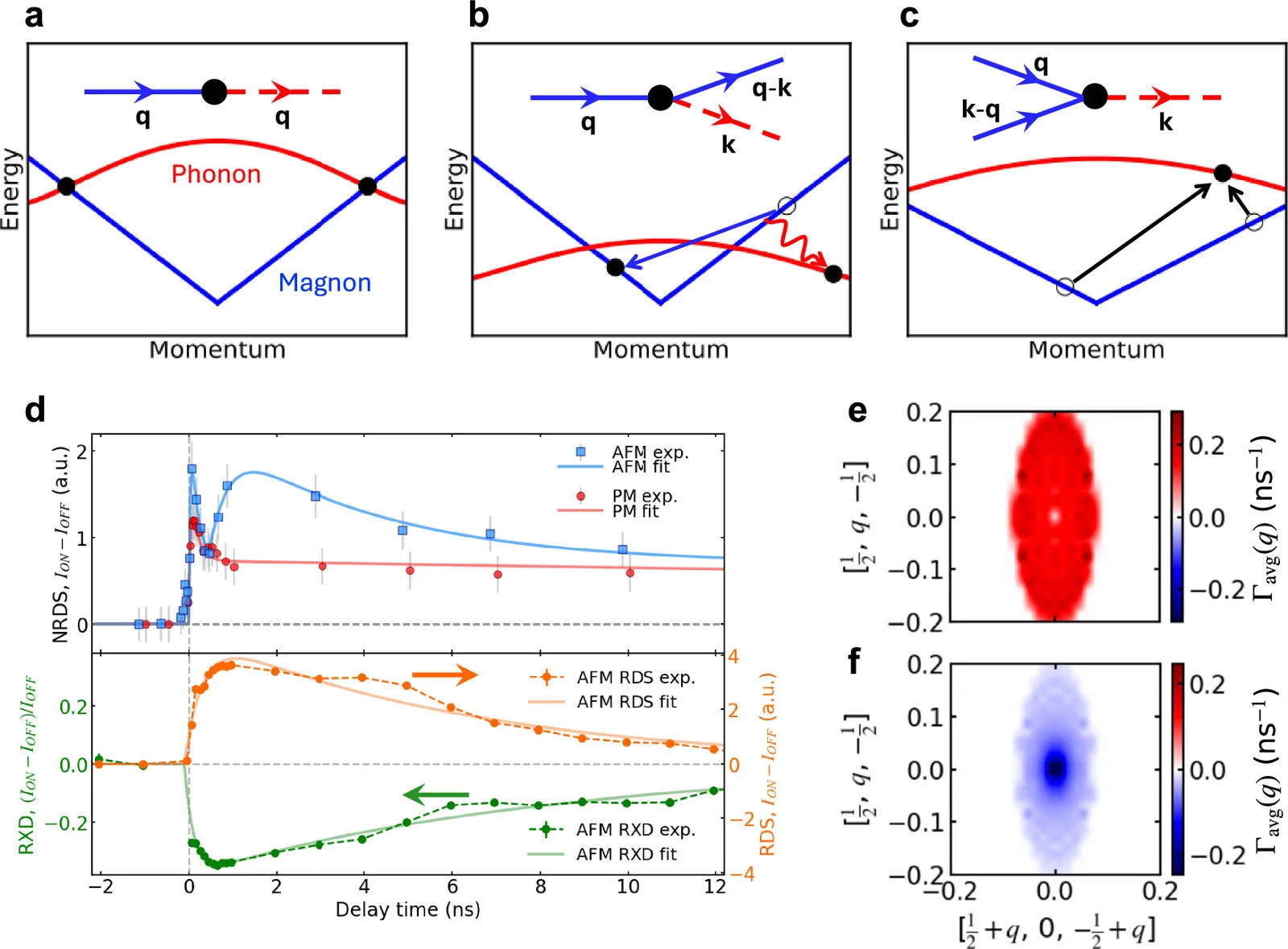
Energy transfer from magnons to phonons. **a–c** Possible magnon-phonon coupling mechanisms: **a** magnon-phonon interconversion; **b** magnon number-conserved scattering, where a magnon loses energy by creating a phonon; **c** anomalous scattering, where two magnons combine into a single phonon. Insets show corresponding Feynman diagrams, where **q** and **k** represent the momenta of quasiparticles. **d** Long timescale comparison of the (1/2 0 −1/2) RXD intensity (green), RDS intensity at (0.47, 0, −0.53) (orange) (bottom), and NRDS intensities integrated around the (0 0 −2) Bragg peak within 0.1 r.l.u. (see Fig. S11) in the collinear AFM (blue) and PM (red) phases (top), revealing a correlation between magnon annihilation and phonon creation during magnetic recovery. The used pump laser fluence was 3 mJ cm^−2^. Error bars represent standard error. **e**,** f** Simulated two-dimensional distribution patterns of the scattering rate for the lowest-energy optical phonons (**e**) and the acoustic magnons (**f**) via the anomalous scattering, calculated using the simplified orthorhombic unit cell.

After quasi-thermalization through magnon-magnon scattering, the suppressed AFM order starts to recover at significantly later timescales with a ~7 ns time constant, as diagnosed from the restoration of the RXD intensity (Fig. 4d, green). The RDS intensity at (0.47, 0, −0.53) shows a similar time constant (orange) to the RXD time trace. This demonstrates that magnon annihilation restores the AFM ground state.

The spin gap at the Γ point prevents magnons from full relaxation solely via magnon-magnon scattering. To fully recover the AFM order, phonons provide the most plausible energy sink for magnon annihilation. Supporting this relaxation mechanism, tr-NRDS intensity around the (0 0 −2) Bragg reflection collected in the AFM phase exhibits a broad enhancement around 2 ns, a similar timescale to the RDS signals (Fig. 4d, blue), indicating phonon creation during the magnon annihilation process. The occurrence of this process suggests that the magnon diffusion process is inefficient, likely due to the magnon accumulation around the Γ point after quasi-thermalization. This enhancement is absent in the paramagnetic phase (red), where magnon annihilation and phonon creation are unnecessary for relaxation since spins are already thermally randomized. Note that this enhancement is not caused by a shift of the Bragg peak due to thermal expansion (see Supplementary Note 9 and Fig. S11c). The short-lived peak with a ~160 ps time constant observed in both phases immediately after excitation arises from photo-created phonons^44^ (Fig. 1a) or via magnon-number conserved scattering (see below) and provides the phonon quasi-thermalization timescale. The tr-NRDS signals arise predominantly where equilibrium phonons exist (Figs. S11a, c), consistent with the faster phonon quasi-thermalization timescale compared to the phonon creation timescale via magnon annihilation.

There are three major pathways to decay energies from magnons into phonons based on a harmonic oscillator coupled to magnons^45^ (Supplementary Note 10): (i) the magnon-phonon interconversion process at their crossing point (Fig. 4a), (ii) number-conserving magnon-phonon scattering, where a magnon loses energy by creating a phonon (Fig. 4b), and (iii) the anomalous scattering process, where two magnons combine into a single phonon (Fig. 4c). The RDS decay (Fig. 4d, orange) demonstrates that magnon annihilation occurs on nanosecond timescales. Of the three lowest-order magnon-phonon coupling processes, only (i) and (iii) can annihilate magnons, while (ii) conserves magnon number, likely contributing to the short-lived peak in tr-NRDS signals for quasi-thermalization. Efficient scattering requires phonon energies comparable to magnon energies, severely restricting the available phase space due to energy and momentum conservation. (i) is especially inefficient because the reciprocal space volume occupied by the intersecting points is tiny. Besides the spin gap and irrelevant magnon diffusion, the inefficient coupling strengths impose an intrinsic bottleneck for the magnetic recovery.

To elucidate the dominant magnon-phonon coupling mechanism, we calculated the respective scattering rates based on the quantum Boltzmann equation (Supplementary Note 10), assuming the quasi-thermalized magnon distribution after magnon-magnon scattering as the initial state. The anomalous process between the lowest-energy optical phonons and the acoustic magnons is found as the dominant magnon decay channel [see Fig. 4e (phonons) and f (magnons) for their scattering rates; see also Fig. S12]. The characteristic pattern is due to the phase-space constraints for the scattering process. The effective magnon-phonon coupling strength via the anomalous scattering estimated from the magnon relaxation timescale (Fig. 4d) is ~59 μeV, while spin-phonon coupling in CuO can be as large as ~6.2 meV^46^. Even though spin-lattice coupling strength in CuO is expected to be generally large, as manifested by multiferroicity^47^ and their persistence correlation at high temperatures^48^, the strong restriction of the available phase space results in the unexpectedly small effective magnon-phonon coupling strength. This suggests the possibility of designing the lifetime of photo-induced intermediate states by exploiting corresponding quasiparticle dispersions.

## Discussion

In summary, our observations establish the complete hierarchical energy transfer pathways in non-equilibrium states associated with ultrafast sublattice demagnetization in AFM CuO. Although respective timescales may differ depending on scattering efficiencies, the processes are generalizable to any gapped magnetic insulators, including ferromagnets when they break the SU(2) symmetry, beyond multi-temperature models^8^. (1) At first, photoexcitation creates spin disorder via a high-energy electronic configuration, which quickly recovers on femtosecond timescales, leaving significant excitation energy in the spin sector. This results in non-thermal low-energy magnons distributed throughout the Brillouin zone (Fig. 3j), alongside photo-created phonons. (2) Magnon-magnon scattering drives the magnetic system into quasi-equilibrium on picosecond timescales, with magnons accumulating towards the spin gap (Fig. 3j–n), alongside possible magnon decays from high-energy branches and magnon-conserving scattering with phonons. (3) Magnon-phonon coupling releases energy from the spin system to phonons and recovers the magnetic order on nanosecond timescales via the anomalous scattering, annihilating magnons (Fig. 4f). The principle that dispersion overlap between magnons and phonons controls coupling efficiency applies generally to control the lifetime of non-equilibrium states by engineering the dispersions. (4) Phonon annihilation recovers the equilibrium state by transporting energy away on microsecond timescales (Fig. S13). This process may be dominated by electron-phonon scattering rather than phonon diffusion in CuO because of the temperature dependence of the phonon annihilation time constant (Supplementary Note 11).

Direct observation of these processes remained experimentally challenging, as it requires sensitivity to multiple subsystems with momentum resolution at ultrafast timescales. The time- and momentum-resolved view of magnon and phonon dynamics addresses the fundamental questions of ultrafast magnetism, crucial for realizing high-speed, high-repetition devices: (1) energy transfer pathways, (2) subsystem coupling efficiencies, and (3) dynamical bottlenecks. The established general framework predicts that materials with strongly overlapping dispersions of magnons and phonons will exhibit faster recovery, enabling rational design of device operation speeds.

While tr-NRDS accesses low-energy lattice excitations^21^, tr-RDS emerges as a transformative tool to investigate low-energy electronic counterparts in non-equilibrium states. This new capability complements tr-RIXS, which accesses a variety of high-energy fundamental excitations, including magnons, orbital excitations, excitons, plasmons, and polarons^49^, indicating the applicability of tr-RDS to a wide class of materials^50^, including superconductors^2^, quantum paraelectrics^3^, and systems controlled via Floquet engineering^51^. Low-energy quasiparticles often dominate physics on various timescales, including phase transitions, transport phenomena, and non-equilibrium states. The ability to track low-energy quasiparticle kinetics across multiple subsystems in real time provides unprecedented insight into non-equilibrium states and energy flow pathways, and determines mode-selective coupling strength. Its independence from Fourier-transform limits due to energy integration enables, in principle, attosecond-scale investigations of electronic quantum dynamics. Tr-RDS measurements capture the ultrafast dynamics of magnons, which most conventional and newly developed static techniques cannot access because of fundamental limitations in pulse duration and inefficient cross-sections, e.g., inelastic neutron scattering, electron energy-loss spectroscopy^52^, and Mie-enhanced Brillouin light scattering^53^. Our framework establishes the foundation for a new generation of ultrafast control protocols, bridging fundamental condensed matter physics with transformative quantum technologies.

## Methods

### Experimental setup of tr-RXD and tr-RDS

The experimental geometry for time-resolved resonant X-ray diffraction (tr-RXD) and time-resolved resonant diffuse scattering (tr-RDS) measurements, performed at the Furka endstation of the SwissFEL at Paul Scherrer Institute, is illustrated in Fig. S15. A single-crystal CuO sample, oriented with its largest surface parallel to the (1 0 −1) plane, was mounted on a four-circle diffractometer equipped with three sample rotation stages (*θ*, *φ*, *χ*) and two detector rotation stages (2*θ*) in an ultra-high vacuum chamber. Signal detection was achieved using a photodiode for RXD measurements and two reverse-biased avalanche photodiodes for RDS measurements. The active areas of all the detectors were masked with a thin aluminum foil to reject optical photons. The photodiode and one of the avalanche photodiodes were mounted on a single tower assembly to enable simultaneous data acquisition at nearby momentum points. While the photodiode was placed on the horizontal plane, the avalanche photodiode mounted on the same tower was slightly above it. The other avalanche photodiode was on the horizontal plane. Our momentum resolution in tr-RDS measurements is estimated as ~5 × 10^−3^ r.l.u. from the beamline energy resolution (~0.5 eV) and the angular acceptance of the avalanche photodiode (~2.25^∘^). For the investigation of RXD at high excitation fluence, we used a 0.5 M Jungfrau detector. Note that tr-RDS signals were detected only with avalanche photodiodes due to their high quantum efficiency. The X-ray absorption spectrum was measured with an avalanche photodiode in the total fluorescence yield mode. The sample temperature was fixed at 190 K using a cryostat.

The optical pump pulses were generated via second-harmonic generation of fundamental Ti: sapphire laser pulses (800 nm) through a 150 μm-thick *β*-BaB_2_O_4_ crystal, providing 400 nm pulses with *p*-polarization, ~35 fs pulse duration, and 100 Hz repetition rate. Laser fluence *f* is defined as *f* = *p*/*πr*^2^, where *p* and *r* are the pulse energy and the beam waist measured at 1/*e*^2^, respectively. The pump beam was nearly collinear relative to the probe beam using an in-vacuum mirror positioned adjacent to the probe beam path. “Off” shots were collected for normalization by introducing a 28 ns delay to one out of every three pump pulses. The penetration depth of the pump beam is estimated as ~450 nm^54^.

X-ray probe pulses were delivered along the Athos beamline of the SwissFEL^55^, utilizing self-amplified spontaneous emission with photon energies tuned to the Cu *L*_3_ edge (~930 eV) monochromatized with a bandwidth of ~0.5 eV. The pulse duration and repetition rate were ~50 fs and 100 Hz, respectively. X-ray polarization was controlled between *σ* (vertical) and *π* (horizontal) using the Apple X undulators^56^. Transmission gratings were used to perform shot-by-shot photon diagnostics, i.e., measuring arrival time relative to the pump beam and incoming intensity for normalization, without interfering with tr-RXD and tr-RDS measurements. The X-ray probe depth is estimated as ~18 nm on the resonance at the incidence angle (~63.5^∘^) satisfying the magnetic Bragg condition^57^. The combined time resolution is ~60 fs full-width at half-maximum.

### Experimental setup of tr-NRDS

The experimental configuration of time-resolved non-resonant diffuse scattering (tr-NRDS) measurements, conducted at the ID09 beamline of the European Synchrotron Radiation Facility (ESRF), is schematically depicted in Fig. S16. The identical single-crystal sample was mounted on a motorized goniometer with precise angular control. A 1M Jungfrau two-dimensional detector was used to collect diffuse scattering patterns. Sample temperature was controlled between 190 K and 300 K using a nitrogen cryostream. Optical pump pulses were provided by a Ti: sapphire picosecond laser (800 nm wavelength and 1 kHz repetition rate) with optical second-harmonic generation to convert the wavelength to 400 nm using a *β*-BaB_2_O_4_ crystal. The incidence angle of the pump beam was approximately 88^∘^, yielding a penetration depth of ~500 nm. The X-ray probe beam was polychromatic radiation at 8.7 keV below the Cu *K* edge to suppress fluorescence signals. Grazing incidence geometry (~2^∘^) was employed for the probe beam, resulting in the probe depth of ~1.5 μm in the vicinity of the (0 0 −2) Bragg condition. X-ray pulses with ~100 ps pulse duration and *π* polarization were synchronized to the pump laser through a mechanical chopper system^58^, reducing the repetition rate to 1 kHz to match the pump laser. The pump pulse duration in tr-NRDS measurements is longer than in tr-RDS measurements, leading to continuous electronic excitation during the energy dissipation into magnons. However, this effect is likely not significant for the resulting states after optical excitation, because the typical doublon lifetime exceeds the pulse duration in tr-RDS measurements and limits the energy transfer process regardless of pump pulse duration.

### Sample preparation and characterization

A single crystal of CuO was grown using the floating zone technique^59^ and subsequently characterized by powder X-ray diffraction and magnetic susceptibility measurements to confirm phase purity and magnetic ordering temperatures. Magnetic susceptibility measurements reveal two distinct anomalies around *T*_N1_ = 213 K and *T*_N2_ = 230 K, corresponding to successive magnetic phase transitions (Fig. S5). The low-temperature magnetic structure (*T* < *T*_N1_) exhibits collinear antiferromagnetic ordering with propagation vector (1/2, 0, −1/2), while the intermediate temperature range (*T*_N1_ < *T* < *T*_N2_) is characterized by non-collinear spin-spiral ordering with propagation vector (0.506, 0, −0.483)^38^. As visualized through polarized optical microscopy (Fig. S17), the grown crystal contains a minimal volume of twin domains, consistent with previous reports^60^. Contributions from different domains are negligible in our time-resolved X-ray experiments because diffraction selects the properly oriented domain, and diffuse scatterings appear in the vicinity of Bragg reflections. Figure S18a, b shows a diffraction profile and a rocking curve around the (1/2 0 −1/2) magnetic Bragg reflection measured at 929.9 eV, demonstrating the well-defined magnetic ordering and sample quality, respectively. Magnetic scattering produces significant linear polarization dependence.

### DFT calculations

First-principles calculations were performed within density functional theory (DFT) using the Quantum ESPRESSO package^61^. The exchange-correlation functional was treated within the generalized gradient approximation using the Perdew-Burke-Ernzerhof (PBE) functional^62^. To account for strong electronic correlations in the Cu 3*d* orbitals, we employed the DFT + U method with an on-site Hubbard interaction *U *= 7.0 eV applied to the Cu 3*d* states^63^. Antiferromagnetic (AFM) order was imposed in accordance with experimentally established magnetic structures (Fig. S1a). For convenience, the unit cell was rotated such that the crystallographic *b* direction aligns with the spin quantization (*z*) axis. Within this collinear AFM configuration, we obtained an insulating ground state with a band gap (Fig. S1b) of 1.49 eV and a Cu magnetic moment of μ = 0.61μ_B_, in good agreement with experimental values^27,64,65^ and previous theoretical studies^63,66,67^.

### Wannier construction and time-dependent simulations

Following the convergence of the ground-state electronic structure, we constructed a Wannier Hamiltonian using the Wannier90 package^68^. The Wannierization process included Cu-3*d* and O-2*p* orbitals to accurately capture the relevant states. Time-dependent simulations were then performed using the dynamics-w90 code^69^, incorporating an external electric field pulse of the form

4 $$\vec{E}(t)={\vec{E}}_{0}{e}^{-{(t-{t}_{0})}^{2}/2{T}_{p}^{2}}\sin (\omega (t-{t}_{0}))\,,$$

with a pulse duration *T*_*p*_ corresponding to a full width at half maximum (FWHM) of 35 fs and a frequency *ω* = 3.1 eV, consistent with experimental conditions. The density matrix was propagated in time according to the equation of motion

5 $$i\frac{d}{dt}\rho (\vec{k},t)=[H(\vec{k},t),\rho (\vec{k},t)]\,.$$

Combining the occupations after the pump with the orbital-resolved density of states then yields the plot in Fig. 2b.

### Spin model and magnon dispersion

To verify that the effective magnetic interactions underlying the calculations reproduce the experimentally observed spin excitations, we analyzed a simplified spin model derived from the electronic structure. The Cu^2+^ ions in CuO carry localized spin-1/2 moments, which can be described by an effective Heisenberg Hamiltonian obtained from the strong-coupling limit of the Hubbard model.

For this purpose, we adopted a simplified orthorhombic unit cell containing one Cu site per layer, neglecting the small monoclinic distortion and the weak interlayer structural complexity (Fig. S1b). This approximation captures the dominant in-plane magnetic interactions relevant to the low-energy magnon spectrum. The model includes nearest- and next-nearest-neighbor exchange interactions with representative values *J*_AFM_ = 120 meV, *J*_FM_ = −15 meV, and an interlayer coupling of *J*_out_ = −18 meV, as well as a small easy-axis anisotropy *D* = 0.025 meV.

The magnon dispersion was calculated within linear spin-wave theory. The resulting acoustic magnon branch (Fig. S1c) closely reproduces the experimentally measured dispersion obtained from inelastic neutron scattering^38^, validating both the simplified orthorhombic description and the effective magnetic parameters used in the analysis.

### Magnon excitation and magnon scattering

This feedback of charge dynamics onto the spin sector was incorporated by mapping the time-dependent electronic state onto an effective Hubbard model in the Mott-insulator regime. Following ref. ^41^, in the limit of large *U* and weak excitation, the Hubbard model can be approximated by spin excitations coupled to doublons (corresponding to Cu sites with *d*^10^ configuration) and holons (*d*^8^ configuration). The creation of spin waves due to the presence of photoexcitation doublons is then modeled by the sudden ramp of the doublon number and the associated spin-charge coupling. The excitation parameters were chosen to be consistent with the experiment. Details are described in Supplementary Note 1.

The photoexcited magnon distribution is then taken as the initial state for the magnon thermalization dynamics. The magnon-magnon scattering is treated on the level of the quantum Boltzmann equation (QBE), as described in the main text. The scattering integral in Eq. (3) is computed on a *N* = 32 × 48 × 32 grid of k-points in the magnetic Brillouin zone, similar to electron-electron scattering. The magnon-magnon interaction vertex is derived from the effective Heisenberg Hamiltonian by including the leading order beyond linear spin wave theory (see Supplementary Note 7 for details). Similarly, magnon-phonon scattering is modeled by parameterizing the relevant phonon modes known from experiments^70^ and deriving the magnon-phonon coupling from the Heisenberg Hamiltonian. This coupling is then used to define the corresponding QBE scattering integral.

## Supplementary information

Supplementary Information

Transparent Peer Review file

## Data Availability

All data needed to evaluate the conclusions in the main text or the Supplementary Information are available at the PSI Data Repository^71^.
