# Supplementary material for: Hierarchical quasiparticle dynamics in antiferromagnets revealed by time- and momentum-resolved X-ray scattering
Source: Nat Commun. 2026 Sep 29;17:11222. doi: 10.1038/s41467-026-77964-5 (PMC13720009; doi:10.1038/s41467-026-77964-5)
Supplement: Supplementary file 1 [file 41467_2026_77964_MOESM1_ESM.pdf]

Supplementary Information

Hierarchical quasiparticle dynamics in antiferromagnets revealed by time- and momentum-resolved X-ray scattering

Arnau Romaguera, Elizabeth Skoropata, Yun Yen, Biaolong Liu, Abhishek Nag, Shih-Wen Huang, Ludmila Leroy, Katja Sophia Moos, Gian Parusa, Serhane Zerdane, Ritwika Mandal, Céline Mariette, Matteo Levantino, Eugenio Paris, Luc Patthey, Ekaterina Pomjakushina, Urs Staub, Monica Ciomaga Hatnean, Michael Schüler, Elia Razzoli, and Hiroki Ueda*

*Correspondence: hiroki.ueda@psi.ch

This file includes:

Supplementary Notes 1–12

Supplementary Figs. S1–S19

Supplementary Notes

Supplementary Note 1 — Theory of spin-charge coupling in the photoexcited state

The d^9 configuration of the Cu^{2+} ions in CuO gives rise to a localized spin-1/2 moment on each Cu site. This situation can be described by the Hubbard model at half-filling in the limit of strong on-site Coulomb repulsion U:

$$\hat{H} = -t_0 \sum_{\langle \mathbf{R},\mathbf{R}' \rangle,\sigma} \hat{c}^\dagger_{\mathbf{R},\sigma} \hat{c}_{\mathbf{R}',\sigma} + U \sum_{\mathbf{R}} \hat{n}_{\mathbf{R},\uparrow} \hat{n}_{\mathbf{R},\downarrow} \ . \tag{S1}$$

Here, $\hat{c}^\dagger_{\mathbf{R},\sigma}$ ($\hat{c}_{\mathbf{R},\sigma}$) creates (annihilates) an electron with spin σ on site $\mathbf{R}$, and $\hat{n}_{\mathbf{R},\sigma} = \hat{c}^\dagger_{\mathbf{R},\sigma}\hat{c}_{\mathbf{R},\sigma}$ is the corresponding number operator.

In the language of the Hubbard model (S1), the photodoping excitation can be described as the creation of additional doublons (doubly occupied Cu sites). Previous studies on the Hubbard model have already demonstrated the strong impact of photodoping on the AFM order [1]. Furthermore, as shown in Ref. [2], the Coulomb interaction between the photodoped carriers in the ligand orbitals and the upper Hubbard band provides another channel of ultrafast doublon-holon creation. This mechanism is the basis for the ultrafast suppression of AFM order observed in our experiments. Additionally, the O-2p holes can create holons via the coupling to the lower Hubbard band, creating another source of the suppression of AFM order.

When the system is photoexcited, additional doublons (doubly occupied Cu sites) are created. These photodoped carriers can interact with the localized spins, leading to a modification of the exchange interaction and consequently affecting the AFM order. The presence of doublons and holons introduces new channels for spin fluctuations and can enhance spin-charge coupling in the system.

To describe the effect of photodoping on the spin dynamics, we follow the approach from Ref. [3]. In this framework, the Hubbard model (S1) is mapped onto an effective Hamiltonian including doublon, holons, and spin degrees of freedom:

$$\hat{H}_{\text{eff}} = -B_0 \sum_{\langle \mathbf{R},\mathbf{R}' \rangle} \left(\hat{d}^\dagger_{\mathbf{R}} \hat{d}_{\mathbf{R}'} - \hat{e}^\dagger_{\mathbf{R}} \hat{e}_{\mathbf{R}'} \right) \left(\hat{s}_{\mathbf{R}} + \hat{s}^\dagger_{\mathbf{R}'} \right) + \hat{H}_{\text{spin}} \ . \tag{S2}$$

Here, $B_0 = \sqrt{(m+1/2)}t_0$, where m is magnetization. The operators $\hat{d}^\dagger_{\mathbf{R}}$ ($\hat{d}_{\mathbf{R}}$) and $\hat{e}^\dagger_{\mathbf{R}}$ ($\hat{e}_{\mathbf{R}}$) create (annihilate) doublons and holons on site $\mathbf{R}$, respectively. The effective bosonic annihilation operator $\hat{s}_{\mathbf{R}}$ acts on the localized spin-1/2 moment at site $\mathbf{R}$. For the pure Hubbard model, the spin Hamiltonian $\hat{H}_{\text{spin}}$, up to second order in t_0/U, is given by:

$$\hat{H}_{\text{spin}} = 4(2m+1)J\sum_{\mathbf{R}}\hat{s}^\dagger_{\mathbf{R}}\hat{s}_{\mathbf{R}} + \left(m+\frac{1}{2}\right)J\sum_{\langle\mathbf{R},\mathbf{R}'\rangle}\left(\hat{s}^\dagger_{\mathbf{R}}\hat{s}^\dagger_{\mathbf{R}'} + \hat{s}_{\mathbf{R}}\hat{s}_{\mathbf{R}'}\right)\ , \tag{S3}$$

where $J = 4t_0^2/U$ is the exchange interaction between neighboring spins. The spin Hamiltonian (S3) describes the magnon excitations in the AFM state, and it can be paralleled with the linear spin wave theory for the Heisenberg model.

To simplify the following calculations, we consider a simplified unit cell containing only one Cu site by ignoring the atoms between the layers along the c direction (Fig. S1**a**). We further ignore the small monoclinic distortion and treat the system as orthorhombic. Introducing next-nearest-neighbor exchange couplings $J_{\text{FM}} = -15$ meV, $J_{\text{AFM}} = 120$ meV, and $J_{\text{out}} = -18$ meV (see Fig. S1**b**), as well as a small easy-axis anisotropy $D = 0.025$ meV, the magnon dispersion obtained from linear spin-wave theory (Fig. S1**c**) closely resembles the experimentally measured dispersion of the acoustic magnon branch in CuO as determined by inelastic neutron scattering [4]. The values chosen here are approximately consistent with calculated exchange couplings [5].

There are two inequivalent lattice sites in the AFM unit cell; denoting the corresponding bosonic operators $\hat{b}_{\mathbf{R}A} = \hat{s}_{\mathbf{R}}$ for site A and $\hat{b}_{\mathbf{R}B} = \hat{s}_{\mathbf{R}}$ for site B and introducing the Fourier transform $\hat{b}_{\mathbf{k}\alpha} = 1/\sqrt{N}\sum_{\mathbf{R}} e^{-i\mathbf{k}\cdot\mathbf{R}}\hat{b}_{\mathbf{R}j}$ ($j = A, B$), the spin Hamiltonian (S3) can be written as

$$\hat{H}_{\text{spin}} = \sum_{\mathbf{k}} \mathbf{x}^\dagger_{\mathbf{k}}\mathbf{H}(\mathbf{k})\mathbf{x}_{\mathbf{k}}\ . \tag{S4}$$

Here, we introduced the vector $\mathbf{x}_{\mathbf{k}} = (\hat{b}_{\mathbf{k}A}, \hat{b}_{\mathbf{k}B}, \hat{b}^\dagger_{-\mathbf{k}A}, \hat{b}^\dagger_{-\mathbf{k}B})^T$ and the 4×4 matrix

$$\mathbf{H}(\mathbf{k}) = \begin{pmatrix} A(\mathbf{k}) - C & B_{\mathbf{k}} \\ B^\dagger_{\mathbf{k}} & A^*(-\mathbf{k}) - C \end{pmatrix}. \tag{S5}$$

The 2×2 matrices $A(\mathbf{k})$, $B_{\mathbf{k}}$, and C are defined in Ref. [6] with the exchange couplings given above. Diagonalizing the Hamiltonian (S4) using a Bogoliubov-like transformation yields the magnon eigenmodes $\hat{\alpha}_{\mathbf{k}\nu}$, $\nu = 1, 2$, with energies $\omega_\nu(\mathbf{k})$, respectively. The transformation is given by

$$\hat{b}_{\mathbf{k}j} = \sum_{\nu=1,2}\left[Q_{j\nu}(\mathbf{k})\hat{\alpha}_{\mathbf{k}\nu} + \bar{Q}_{j\nu}(\mathbf{k})\hat{\alpha}^\dagger_{-\mathbf{k}\nu}\right]\ ; \tag{S6}$$

the coefficients $Q_{j\nu}(\mathbf{k})$ and $\bar{Q}_{j\nu}(\mathbf{k})$ are obtained from the decomposition of Eq. (33) in Ref. [6]. Substituting $\hat{s}_{\mathbf{R}} = 1/\sqrt{N}\sum_{\mathbf{k}} e^{i\mathbf{k}\cdot\mathbf{R}}\hat{b}_{\mathbf{k}j}$ (for site $j = A, B$) and using the Bogoliubov transformation (S6), the effective Hamiltonian (S2) can be expressed in terms of the magnon operators $\hat{\alpha}_{\mathbf{k}\nu}$, doublon operators $\hat{d}_{\mathbf{k}}$, and holon operators $\hat{e}_{\mathbf{k}}$. One finds

$$\hat{H}_{\text{eff}} = -\frac{B_0}{\sqrt{N}}\sum_{\mathbf{k},\mathbf{q}}\sum_{\nu=1,2}\left[\left(\hat{d}^{\dagger}_{\mathbf{k}A}\hat{d}_{\mathbf{k}-\mathbf{q}B} - \hat{e}^{\dagger}_{\mathbf{k}A}\hat{e}_{\mathbf{k}-\mathbf{q}B}\right)\left(Q_{A\nu}(\mathbf{q})\hat{\alpha}_{\mathbf{q}\nu} + \bar{Q}_{B\nu}(\mathbf{q})\hat{\alpha}^{\dagger}_{-\mathbf{q}\nu}\right) + (A \leftrightarrow B)\right] + \hat{H}_{\text{spin}} . \tag{S7}$$

The Hamiltonian (S7) constitutes an interacting fermion-boson model, which in its full complexity is difficult to solve. To make further progress, we treat the doublons and holons as classical particles within a mean-field approximation. This is justified since in the experiments the photodoped carriers are generated incoherently by the pump pulse. Within this approximation, we replace the doublon and holon operators in Eq. (S7) by their expectation values $\langle \hat{d}^{\dagger}_{\mathbf{k}A}\hat{d}_{\mathbf{k}'B}\rangle = \sqrt{N}n_d\delta_{\mathbf{k},\mathbf{k}'}$ and $\langle \hat{e}^{\dagger}_{\mathbf{k}A}\hat{e}_{\mathbf{k}'B}\rangle = \sqrt{N}n_h\delta_{\mathbf{k},\mathbf{k}'}$, where n_d and n_h are the doublon and holon densities, respectively. Since the exchange couplings have been chosen to reproduce the experimental magnon dispersion, we can neglect renormalization effects in the ground state. Thus, we only consider terms proportional to the time-dependent change of the doublon and holon densities. We then obtain the time-dependent magnon Hamiltonian

$$\hat{H}_{\text{mag}}(t) = \sum_{\mathbf{q},\nu}\omega_\nu(\mathbf{q})\hat{\alpha}^{\dagger}_{\mathbf{q}\nu}\hat{\alpha}_{\mathbf{q}\nu} - B_0\sum_{\mathbf{q},\nu}\Delta n(t)\left[\left(Q_{A\nu}(\mathbf{q}) + Q_{B\nu}(\mathbf{q})\right)\hat{\alpha}_{\mathbf{q}\nu} + \left(\bar{Q}_{A\nu}(\mathbf{q}) + \bar{Q}_{B\nu}(\mathbf{q})\right)\hat{\alpha}^{\dagger}_{-\mathbf{q}\nu}\right] . \tag{S8}$$

Here, we introduced the total photodoped carrier density $\Delta n(t) = \Delta n_d(t)$, where $\Delta n_d(t)$ is the time-dependent changes in the doublon densities (the number of holons remains approximately constant).

Equation (S8) describes how magnons are excited by the time-dependent photodoped carrier density $\Delta n(t)$. The solution of Eq. (S8) is given in terms of a driven harmonic oscillator. Assuming that initially the magnons are in thermal equilibrium at temperature T, the time-dependent magnon occupation is given by

$$f_\nu(\mathbf{q}, t) = f^{\text{eq}}_\nu(\mathbf{q}) + |y_{\mathbf{q}\nu}(t)|^2 , \tag{S9}$$

where $f_\nu^{\text{eq}}(\mathbf{q})$ is given by the equilibrium Bose-Einstein distribution and

$$\dot{y}_{\mathbf{q}\nu}(t) = -i\omega_\nu(\mathbf{q})y_{\mathbf{q}\nu}(t) - iB_0\Delta n(t)\left(\bar{Q}_{A\nu}(\mathbf{q}) + \bar{Q}_{B\nu}(\mathbf{q})\right) . \tag{S10}$$

For simplicity, the time-dependence of $\Delta n(t)$ is modeled as a smooth step function with a rise time of 25 fs, which is consistent with the simulations of the electronic dynamics described above. To estimate the effective strength of the spin-charge coupling, we compute the sublattice magnetization $S_{A,B}$ per Cu site after photoexcitation using Eq. (50) in Ref. [6]. For the spin-1/2 model considered here, $S_A^{\text{eq}} = 0.39\hbar$ in equilibrium at $T = 190$ K. We then chose the amplitude of $B_0\Delta n(t)$ to reproduce the experimentally observed reduction of the AFM order by approximately 50%.

The resulting magnon occupation after photoexcitation (Fig. S2**b**) is significantly non-thermal, with a broad distribution of magnons excited across the entire Brillouin zone. Similar non-thermal magnon distributions have been reported in Ref. [7].

Supplementary Note 2 — Deconvolution of RXD and RDS signals

RDS signals exhibit enhanced intensity in the vicinity of the (1/2 0 −1/2) magnetic Bragg reflection due to the energy minimum in the magnon dispersion, leading to a large population of the bosonic excitations. Resonant X-ray scattering measurements inherently contain contributions from both RXD and RDS components, which show distinct dynamics following photoexcitation. While ultrafast demagnetization produces a quasi-instantaneous decrease in RXD intensity, the subsequent creation and redistribution of magnon density manifest as an increase in RDS intensity at later timescales. To capture these dynamics, we analyzed data using two exponential functions:

$$I_{\text{ON}}(t) - I_{\text{OFF}} = A_{\text{RXD}} \exp\left(-t/\tau_{\text{RXD}}\right) + A_{\text{RDS}} \exp\left(-t/\tau_{\text{RDS}}\right) . \tag{S11}$$

Here, A_{RXD} (< 0) and A_{RDS} (> 0) represent the amplitude, and τ_{RXD} and τ_{RDS} denote the time constant for the photo-induced changes in RXD and RDS intensities, respectively. The deconvolution of the momentum-dependent data presented in Fig. 3**a** into the RXD and RDS components is shown in Figs. S3**a**–S3**c** using this model.

Supplementary Note 3 — ”Temperature” estimation

The non-thermally distributed magnon densities allow us to estimate corresponding ”temperatures” as a function of momentum. When an excitation predominantly couples to a specific mode, the phenomenological temperature model requires subdividing the subsystem: for example, hot optical phonons in the lattice[8]. In our case, the spin system is subdivided into hot acoustic magnons. Hot-magnon temperatures at fast timescales, estimated from the simulated magnon density assuming a Boltzmann distribution, reach ~500 K at $(0.5, 0.1, -0.5)$, far exceeding the bulk quasi-equilibrium limit of ~210 K. This indicates that the excitation energy initially deposited in the electronic system, resulting in doublon creation that quickly decays, transfers into the magnonic subsystem at ultrafast timescales, while the electronic states return to equilibrium [9, 10] and the average temperature, except for hot carriers, remains below T_{N1}.

Supplementary Note 4 — Transient incommensurate state at high excitation regime

As shown in the main text, moderate excitation fluence leads to strongly non-thermal magnons but keeps the long-range collinear AFM state because the average temperature remains below T_{N1}. Although a photo-induced phase transition into the incommensurate phase [11] could result in an upturn signal in a certain momentum range via the transient appearance of a corresponding magnetic Bragg peak, it does not explain our experimental observation in the moderate fluence regime. This is because (i) the measured momentum point $(0.49, 0.01, -0.51)$ is distant from the incommensurate peak position at $(0.506, 0, -0.483)$, (ii) it does not explain the systematic tail intensity increase around the magnetic Bragg peak (Fig. 2**c**) and (iii) the momentum-dependent time constant (Fig. 3**f**), and (iv) we did not experimentally detect any significant upturn signal at the incommensurate peak position compared to other momentum points after the photoexcitation within our experimental precision. The overall temperature increase estimated by the specific heat [12] and deposited energy density is below 20 K ($< T_{\mathrm{N1}}$), indicating the remaining collinear AFM state after the photoexcitation. The constant width (0.6°) of rocking curve scans with and without the optical excitation, shown in Fig. S4, confirms the constant correlation length of the AFM order. This observation indicates that we do not melt the AFM order into the disordered phase but rather

reduce the amplitude of the AFM order parameter.

Increasing the excitation fluence raises the average temperature above T_{N1} and, as expected from the equilibrium phase diagram (Fig. S5), stabilizes the incommensurate spin-spiral phase, consistent with the previous study[11]. Figure S6**a** shows a two-dimensional RXD profile at 14.7 ps after the photoexcitation. While the region with an intensity decrease corresponds to the commensurate peak, the region with an intensity increase corresponds to the incommensurate peak that appears transiently. Figure S6**b** shows the upturn (top row) and reduction (bottom row) amplitudes and time constants of integrated time traces in multiple regions of interests along the direction connecting the two peak positions. While the reduction amplitude is symmetric around the commensurate peak, the upturn amplitude and time constant are symmetric around the incommensurate peak, which is in contrast to the tr-RDS signals symmetric around $\mathbf{q}_{\mathrm{RDS}}$ (see main text). The larger time constant of the incommensurate peak time traces than the previous study [11] is due to the absence of the coexistence of the two orders, which may appear in a specific temperature window. The incommensurate order can respond rapidly by moving domain walls when the two phases coexist.

Supplementary Note 5 — Theory of RDS of magnon origin

The magnetic diffuse scattering intensity is calculated based on Kramers-Heisenberg equation [13]. At $T = 0$, only the ground state is taken into account as initial state. Scattering intensity with incoming (outgoing) photon momentum, photon energy, and polarization $\mathbf{k}_i, \omega_i, \boldsymbol{\epsilon}_i$ ($\mathbf{k}_o, \omega_o, \boldsymbol{\epsilon}_o$) can be written as

$$I_{\mathrm{DS}}(\mathbf{k}_i, \mathbf{k}_o, \omega_i, \omega_o, \epsilon_i, \epsilon_o) = \sum_f |\langle f|\hat{D}_o^\dagger \frac{1}{\omega_i + E_g + i\tau_n - \hat{H}_n} \hat{D}_i|g\rangle|^2 L_{\tau_f}(\omega_i - \omega_o + E_g - E_f)\ , \tag{S12}$$

where $\hat{D}_i$ ($\hat{D}_o$) is light-matter dipole operator for incoming (outgoing) photon, which excites one core electron into the valence orbital. $|g\rangle$ is the initial state (before scattering) with energy E_g and $|f\rangle$ is the final state (after scattering) with energy E_f. The propagator in between dipole operators uses Hamiltonian $\hat{H}_n$ in the presence of one excited core-hole and a corresponding lifetime τ_n. $L_{\tau_f}(E)$ is a Lorentzian with lifetime broadening τ_f. We simplify the scattering process by defining

a local transition operator $\hat{T}_{io}^{\mathbf{R}}$ [14] as

$$\hat{T}_{io}^{\mathbf{R}} \equiv \hat{D}_o^{\mathbf{R}\dagger} \frac{1}{\omega_i + E_g + i\tau_n - \hat{H}_n} \hat{D}_i^{\mathbf{R}} . \tag{S13}$$

Here the local dipole operator $\hat{D}^{\mathbf{R}}$ ($\hat{D}^{\mathbf{R}\dagger}$) annihilate (create) a core-hole at site $\mathbf{R}$. The local transition operator $\hat{T}_{io}^{\mathbf{R}}$ is defined so that the incoming and outgoing photons act on the same site $\mathbf{R}$, and its Fourier transformation is defined as $\hat{T}_{io}^{\mathbf{q}} = \sum_{\mathbf{R}} e^{i\mathbf{q}\cdot\mathbf{R}} \hat{T}_{io}^{\mathbf{R}}$. We now restrict the possible excitations ($|l\rangle$) to be within a small energy range $\omega_i - \omega_o \approx 0$ on the scale of the electronic spectrum. The cross-section is then separated into an *elastic* and *inelastic* part as

$$\begin{aligned} I_{\mathrm{DS}}(\mathbf{q} = \mathbf{k}_i - \mathbf{k}_o, \omega_i - \omega_o \to 0) &\approx |\langle g|\hat{T}_{io}^{\mathbf{q}}|g\rangle|^2 \\ &+ \sum_{l \neq g, E_l - E_g \to 0} \langle g|\hat{T}_{io}^{\mathbf{q}\dagger}|l\rangle\langle l|\hat{T}_{io}^{\mathbf{q}}|g\rangle L_{\tau_f}(\omega_i - \omega_o + E_g - E_l), \end{aligned} \tag{S14}$$

where in the second line the summation runs over low-energy excitation $|l\rangle$ close to the ground states. The form of transition operator $\hat{T}_{io}^{\mathbf{R}}$ can be related to the optical conductivity tensor [14, 15]. If site $\mathbf{R}$ has local magnetization [14], the matrix elements of $\hat{T}_{io}^{\mathbf{q}}$ in Eq. (S14) can be approximated as matrix elements of a series of quantum spin operators [16, 14]. Here, we approximate the matrix element expression to the first order, i.e., one spin operator for one $\hat{T}_{io}^{\mathbf{R}}$. In our case of magnetic diffuse scattering in CuO, we approximate the point group symmetries of all Cu sites to be spherical symmetry for simplification. The time-dependent scattering signal is then calculated by the time-dependent magnon distribution, simulated with QBE. Now we denote the time-dependent excited magnon distribution as $|g(t)\rangle$. For incoming (outgoing) light polarization $\boldsymbol{\epsilon}_i$ ($\boldsymbol{\epsilon}_o$), the first elastic term in the right-hand side of Eq. (S14) can be simplified as

$$I_{io}^{\mathrm{elastic}}(t) = |\langle g(t)|\hat{T}_{io}^{\mathbf{q}}|g(t)\rangle|^2 \sim |\langle g(t)| \sum_{\alpha=x,y,z} c_\alpha \sum_{\mathbf{R},j} e^{i\mathbf{q}\cdot(\mathbf{R}+\mathbf{r}_j)} \hat{S}_{\mathbf{R}j}^{\alpha} |g(t)\rangle|^2 = |\langle g(t)| \sum_{\alpha} c_\alpha \hat{S}^{\alpha}(\mathbf{q})|g(t)\rangle|^2 , \tag{S15}$$

where the coefficient $c_\alpha = [\boldsymbol{\epsilon}_i \times \boldsymbol{\epsilon}_o]_\alpha$ reflects the experimental scattering geometry. The Fourier transformation of spin operator is defined as $\hat{S}^{\alpha}(\mathbf{q}) = \sum_{\mathbf{R}} e^{i\mathbf{q}\cdot\mathbf{R}} \hat{S}_{\mathbf{R}}^{\alpha}$. Equation (S15) measures the *magnetic Bragg peak* with extra coefficient c_α. Similarly, the second inelastic term in Eq. (S14)

reads as

$$I_{io}^{\text{inelastic}}(t) \propto \sum_{l} \langle g(t)|\hat{T}_{io}^{\mathbf{q}\dagger}|l\rangle\langle l|\hat{T}_{io}^{\mathbf{q}}|g(t)\rangle \sim \sum_{\alpha\alpha'} c_{\alpha}^{*} c_{\alpha'} \sum_{l} \langle g(t)|\hat{S}^{\alpha}(-\mathbf{q})|l\rangle\langle l|\hat{S}^{\alpha'}(\mathbf{q})|g(t)\rangle$$
$$= \frac{1}{N} \sum_{\mathbf{R}\mathbf{R}'} \sum_{jj'} e^{i\mathbf{q}\cdot(\mathbf{R}-\mathbf{R}'+\mathbf{r}_j+\mathbf{r}_{j'})} \sum_{\alpha\alpha'} F_{\alpha\alpha'} \langle g(t)|\hat{S}_{\mathbf{R}j}^{\alpha} \hat{S}_{\mathbf{R}'j'}^{\alpha'}|g(t)\rangle \,, \tag{S16}$$

where N refers to the number of unit cells and where j, j' run over the sublattice sites. The last term in Eq. (S16) defines the spin-spin correlation function

$$S_{\alpha\alpha'}(\mathbf{q}, t) = \frac{1}{N} \sum_{\mathbf{R}\mathbf{R}'} \sum_{jj'} e^{i\mathbf{q}\cdot(\mathbf{R}-\mathbf{R}'+\mathbf{r}_j+\mathbf{r}_{j'})} \langle g(t)|\hat{S}_{\mathbf{R}j}^{\alpha} \hat{S}_{\mathbf{R}'j'}^{\alpha'}|g(t)\rangle \,. \tag{S17}$$

Hence, the inelastic scattering signal (S16) directly reveals the spin-spin correlation function

Equation (S16) is exactly spin structural factor with extra prefactor $F_{\alpha\alpha'} = c_{\alpha}^{*} c_{\alpha'}$, which is determined by the scattering geometry. Next, the spin operators in Eq. (S16) are expressed with bosonic operators with Holstein-Primakoff transformation, and further transformed to magnon eigenmodes as in Eq. (S6). We obtain

$$I_{io}^{\text{inelastic}}(t) \propto \sum_{\alpha\alpha'} F_{\alpha\alpha'} \sum_{\nu} \left[A_{\nu}(\mathbf{q}) f_{\nu}(\mathbf{q}, t) + \bar{A}_{\nu}(\mathbf{q}) \bar{f}_{\nu}(\mathbf{q}, t) \right] \,, \tag{S18}$$

where the coefficients $A_{\nu}(\mathbf{q})$, and $\bar{A}_{\nu}(\mathbf{q})$ are calculated from the transformation coefficients $Q_{j\nu}$ and $\bar{Q}_{j\nu}$ in Eq. (S6), and where $\bar{f}_{\nu} = f_{\nu} + 1$. Equation (S18) can be directly evaluated based on time-dependent magnon distribution simulated from QBE. We include both s and p polarizations in the geometric coefficient $F_{\alpha\alpha'} = c_{\alpha}^{s*} c_{\alpha}^{s} + c_{\alpha}^{p*} c_{\alpha}^{p}$, and they are calculated based on experimental geometry as

$$\begin{aligned} &F_{xx} = 0.505, F_{xy} = 0.453, F_{xz} = -0.130, \\ &F_{yx} = 0.453, F_{yy} = 0.415, F_{yz} = -0.037, \\ &F_{zx} = -0.130, F_{zy} = -0.037, F_{zz} = 0.822. \end{aligned} \tag{S19}$$

The coordinate system for the light polarization is the same as for the spin orientation, sketched in Fig. S1**a**.

Supplementary Note 6 — Momentum dependence along $[h\ 0\ h]$

We measured the momentum dependence of tr-RDS time traces along $[h\ 0\ h]$ (Fig. S7), in addition to along $\mathbf{b}^*$ (Fig. 3). The same fitting procedure using Eq. S11 allows us to decompose the time traces into RXD (Fig. S7**b**) and RDS (Fig. S7**d**) components. While the RXD component shows a similar amplitude and the same time constant within the error bars as the dataset along $\mathbf{b}^*$, the RDS component shows substantially larger upturn signals. This indicates strong anisotropy in the monoclinic structure.

Supplementary Note 7 — Theory of magnon-magnon scattering

After their initial excitation, the magnons undergo magnon-magnon scattering processes, redistributing energy and momentum until a thermal magnon distribution is reached. To model this thermalization process, we employ a quantum Boltzmann equation (QBE) for the magnon occupation $f(\mathbf{k}, t)$:

$$\frac{d}{dt} f_\nu(\mathbf{k}, t) = S_\nu[f](\mathbf{k}, t) \ . \tag{S20}$$

Note that the two magnon branches $\nu = 1, 2$ are degenerate in our simplified model. The magnon-magnon scattering term $S_\nu[f](\mathbf{k}, t)$ is derived by going to fourth order in the Holstein-Primakoff expansion of the underlying Heisenberg model, as demonstrated in Ref. [17] for the square lattice. Here we generalize this approach to the present case of CuO with the exchange couplings introduced above. To this end, we start from Heisenberg Hamiltonian (now $\mathbf{R}$ runs over all lattice vectors in the magnetic unit cell, while $j, j' = A, B$ denote the two sublattices):

$$\hat{H}_{\text{Heis}} = \sum_{\langle \mathbf{R},\mathbf{R}' \rangle} \sum_{jj'} J_{jj'}(\mathbf{R} - \mathbf{R}') \hat{\mathbf{S}}_{\mathbf{R}j} \cdot \hat{\mathbf{S}}_{\mathbf{R}'j'} = \sum_{\langle \mathbf{R},\mathbf{R}' \rangle} \sum_{jj'} J_{jj'}(\mathbf{R} - \mathbf{R}') \hat{\mathbf{S}}'_{\mathbf{R}i} \mathbf{V}_j^T \mathbf{V}_{j'} \hat{\mathbf{S}}'_{\mathbf{R}'j'} \ , \tag{S21}$$

where $\hat{\mathbf{S}}'_{\mathbf{R}} = \mathbf{V}_j^T \hat{\mathbf{S}}_{\mathbf{R}}$ are the spin operators in the rotated frame where all spins are aligned. It is convenient to express the rotation matrices $\mathbf{V}_j$ in terms the vectors $\mathbf{u}_j$, $\mathbf{v}_j$ as in Ref. [6]. Expanding the Holstein-Primakoff transformation up to fourth order and defining $J'_{j,j'}(\mathbf{R}) = \mathbf{V}_j^T J_{jj'}(\mathbf{R}) \mathbf{V}_{j'}$,

we obtain the quartic magnon Hamiltonian

$$\hat{H}_{\mathrm{mag-mag}} = -\frac{1}{8}\sum_{\mathbf{R},\mathbf{R}'}\sum_{j,j'} J'_{jj'}(\mathbf{R}-\mathbf{R}')\Big[\mathbf{u}_j^\dagger\mathbf{u}_{j'}^*\hat{b}_{\mathbf{R}j}\hat{b}^\dagger_{\mathbf{R}'j'}\hat{b}_{\mathbf{R}'j'}\hat{b}_{\mathbf{R}'j'} + \mathbf{u}_j^\dagger\mathbf{u}_{j'}\hat{b}_{\mathbf{R}j}\hat{b}^\dagger_{\mathbf{R}'j'}\hat{b}^\dagger_{\mathbf{R}'j'}\hat{b}_{\mathbf{R}'j'} + \mathbf{u}_j^\dagger\mathbf{u}_{j'}^*\hat{b}^\dagger_{\mathbf{R}j}\hat{b}_{\mathbf{R}j}\hat{b}_{\mathbf{R}j}\hat{b}_{\mathbf{R}'j'} + \mathbf{u}_j^\dagger\mathbf{u}_{j'}\hat{b}^\dagger_{\mathbf{R}j}\hat{b}_{\mathbf{R}j}\hat{b}_{\mathbf{R}j}\hat{b}^\dagger_{\mathbf{R}'j'} + \mathrm{H.c.}\Big] . \tag{S22}$$

Fourier transforming Eq. (S22) yields $\hat{H}_{\mathrm{mag-mag}} = -(\hat{V}^{(1)} + \hat{V}^{(2)} + \mathrm{H.c.})/4$ with

$$\hat{V}^{(1)} = \frac{1}{N}\sum_{j,j'}\sum_{\mathbf{k}_1,\mathbf{k}_2,\mathbf{k}_3,\mathbf{k}_4}\delta_{\mathbf{k}_1,\mathbf{k}_2-\mathbf{k}_3-\mathbf{k}_4}A^*_{jj'}(\mathbf{k}_1)\hat{b}_{\mathbf{k}_1 j}\hat{b}^\dagger_{\mathbf{k}_2 j'}\hat{b}_{\mathbf{k}_3 j'}\hat{b}_{\mathbf{k}_4 j'} , \tag{S23}$$

$$\hat{V}^{(2)} = \frac{1}{N}\sum_{j,j'}\sum_{\mathbf{k}_1,\mathbf{k}_2,\mathbf{k}_3,\mathbf{k}_4}\delta_{\mathbf{k}_1,\mathbf{k}_2+\mathbf{k}_3-\mathbf{k}_4}B_{jj'}(\mathbf{k}_1)\hat{b}_{\mathbf{k}_1 j}\hat{b}^\dagger_{\mathbf{k}_2 j'}\hat{b}^\dagger_{\mathbf{k}_3 j'}\hat{b}_{\mathbf{k}_4 j'} , \tag{S24}$$

where we have defined the coefficients $A_{jj'}(\mathbf{k}) = \mathbf{u}_j^T\mathbf{u}_{j'}J_{jj'}(-\mathbf{k})$ and $B_{jj'}(\mathbf{k}) = \mathbf{u}_j^\dagger\mathbf{u}_{j'}J_{jj'}(\mathbf{k})$. Finally, substituting the transformation (S6) into Eqs. (S23), we obtain the magnon-magnon interaction in terms of the magnon eigenmodes $\hat{\alpha}_{\mathbf{k}\nu}$:

$$\hat{H}_{\mathrm{mag-mag}} = \frac{1}{N^2}\sum_{\mathbf{k}_1,\mathbf{k}_2,\mathbf{k}_3,\mathbf{k}_4}\sum_{\nu_1,\nu_2,\nu_3,\nu_4} V_{\nu_1\nu_2\nu_3\nu_4}(\mathbf{k}_1,\mathbf{k}_2,\mathbf{k}_3,\mathbf{k}_4)\hat{\alpha}^\dagger_{\mathbf{k}_1\nu_1}\hat{\alpha}^\dagger_{\mathbf{k}_2\nu_2}\hat{\alpha}_{\mathbf{k}_3\nu_3}\hat{\alpha}_{\mathbf{k}_4\nu_4}\delta_{\mathbf{k}_1+\mathbf{k}_3,\mathbf{k}_2+\mathbf{k}_4} . \tag{S25}$$

Using the magnon-magnon vertex $V_{\nu_1\nu_2\nu_3\nu_4}(\mathbf{k}_1,\mathbf{k}_2,\mathbf{k}_3,\mathbf{k}_4)$ from Eq. (S25) for the degenerate mode $\nu_1 = \nu_2 = \nu_3 = \nu_4$, the magnon-magnon scattering term in the QBE is given by

$$S_\nu[f](\mathbf{k}_1,t) = \frac{2\pi}{N^2}\sum_{\mathbf{k}_2,\mathbf{k}_3,\mathbf{k}_4}\delta_{\mathbf{k}_1+\mathbf{k}_2,\mathbf{k}_3+\mathbf{k}_4}|V_{\nu\nu\nu\nu}(\mathbf{k}_1,\mathbf{k}_2,\mathbf{k}_3,\mathbf{k}_4)|^2\delta(\omega_\nu(\mathbf{k}_1)+\omega_\nu(\mathbf{k}_2)-\omega_\nu(\mathbf{k}_3)-\omega_\nu(\mathbf{k}_4)) \times\left[\bar{f}_\nu(\mathbf{k}_1,t)\bar{f}_\nu(\mathbf{k}_2,t)f_\nu(\mathbf{k}_3,t)f_\nu(\mathbf{k}_4,t) - f_\nu(\mathbf{k}_1,t)f_\nu(\mathbf{k}_2,t)\bar{f}_\nu(\mathbf{k}_3,t)\bar{f}_\nu(\mathbf{k}_4,t)\right] . \tag{S26}$$

Here, $\bar{f}_\nu(\mathbf{k},t) = 1 + f_\nu(\mathbf{k},t)$. We solve the QBE (S20) numerically using a discretization of the Brillouin zone with $N = 32\times48\times32$ **k**-points. The Dirac-delta function is approximated by a biweight kernel with a width of 3.0 meV. The time-stepping of Eq. (S20) is performed using an adaptive Dormand-Prince Runge-Kutta solver.

Figure S8 shows the time-dependent distribution for a few representative time steps. Initially, the distribution shows broad tails extending to high energies. As time progresses, magnon-magnon

scattering leads to a redistribution of magnons towards lower energies. Due the large phase-space volume for magnon-magnon scattering in the three-dimensional system, the magnon distribution close to Γ grows quickly.

To investigate the momentum dependence of the thermalization dynamics in more detail, we plot the time-dependent change of the magnon occupation $\Delta f(\mathbf{q}, t)$ for selected **q**-points in Fig. S9, similar to the experimental analysis. The results show that the magnon occupation shows that away from the magnetic Γ-point, the population dynamics saturates quickly. Closer to Γ, however, the magnon occupation continues to grow even at later times, consistent with the experimental observations.

Supplementary Note 8 — Self-similarity and dynamical scaling

Our microscopic understanding of magnon redistribution from the non-thermal photoexcited state via magnon-magnon scattering is equivalent to the inverse particle cascade, in which a conserved number of particles flows to lower momentum in reciprocal space, discussed in Ref. [18]. This theoretical study on two-dimensional Heisenberg antiferromagnets reported that a long-lived pre-thermal regime, in which an inverse particle cascade occurs, exhibits self-similarity in the fluctuations of the staggered magnetization

$$< M_{-q}(t) M_q(t) >= t^{\alpha} f(t^{\beta}|\mathbf{q}|) \ . \tag{S27}$$

Here, $\alpha = 1.0$, and $\beta = 0.5$. f is the universal function and exhibits scaling $f(x) \sim 1/|x|^{\nu}$ for a broad range of momentum, with $\nu = 2.33$. The universality applies as long as the Heisenberg Hamiltonian preserves the SU(2) symmetry. Our effective Hamiltonian is rather three-dimensional, but of a Heisenberg type. Figure S10**a** shows the momentum dependence of $q^2 I_{\mathrm{RDS}}$, exhibiting a peak that fits well to a Gaussian and indicates the self-similarity across different delays. We used the same spatial-temporal scaling exponents α and β as in Ref. [18] to rescale our tr-RDS signals (data shown in Fig. 3**e**), which measure the spin-spin correlation function (see Fig. S10**b**). The obtained power-law exponent is 2.28 ± 0.08, close to the nominal value (2.33) for the model shown in Ref. [18]. The small deviation may be accounted for by differences in dimensionality and detailed Hamiltonian, a possible non-conserving scattering process, a finite pump pulse duration, and a finite doublon lifetime. Nevertheless, the reasonable agreement supports our microscopic

understanding of magnon-magnon scattering, redistributing magnon density in reciprocal space.

Supplementary Note 9 — Tr-NRDS of phonon origin

As described in the main text, photoexcited magnon energy is transferred to phonons via the anomalous scattering and subsequently dissipated into the lattice, resulting in possible thermal expansion and corresponding Bragg peak shifts. Figures S11**a** and S11**b** display the equilibrium two-dimensional X-ray scattering intensity pattern around the (0 0 −2) Bragg reflection and pump-induced change in the pattern at 3 ns after the photoexcitation, representing a tr-NRDS intensity profile. Phonon density transiently increases along $[0\,0\,l]$ and $[h\,0\,0]$ on top of pre-existing phonons in equilibrium, as marked by streaks, due to less steep acoustic phonon dispersions along these directions compared to other directions [19]. We chose the region of interest between two dashed ellipses with different sizes as shown in Fig. S11**c** to avoid possible contamination by Bragg peak shift, which is clear along $[0\,0\,l]$ at delay times where tr-NRDS signals are less substantial, e.g., at 0.15 ns and 0.6 ns. Consequently, we can conclude that our tr-NRDS time traces are due to diffuse scattering by phonons.

Supplementary Note 10 — Theory of magnon-phonon scattering

We have also investigated the role of magnon-phonon scattering for the recovery dynamics. To this end, we analyzed the scattering term $S_\nu^{\mathrm{mp}}[f](\mathbf{k}, t)$ due to magnon-phonon interactions within the QBE (S20). To derive a microscopic model of magnon-phonon coupling, we start from the Heisenberg Hamiltonian with exchange couplings $J_{jj'}(\mathbf{R}-\mathbf{R}')$ that depend on the atomic positions. Assuming the magnetic exchange couplings depend on the relative distance between two Cu sites, we expand $J_{jj'}(\mathbf{R}-\mathbf{R}')$ to linear order in the atomic displacements $\mathbf{u}_{\mathbf{R}j}$:

$$J_{jj'}(\mathbf{R}-\mathbf{R}') \approx J^0_{jj'}(\mathbf{R}-\mathbf{R}') + \nabla J^0_{jj'}(\mathbf{R}-\mathbf{R}') \cdot \left(\mathbf{u}_{\mathbf{R}j} - \mathbf{u}_{\mathbf{R}'j'}\right) \ , \tag{S28}$$

where $J^0_{jj'}(\mathbf{R}-\mathbf{R}')$ are the equilibrium exchange couplings. This yields the magnon-phonon interaction Hamiltonian

$$\hat{H}_{\mathrm{mag-ph}} = \sum_{\langle \mathbf{R},\mathbf{R}' \rangle} \sum_{jj'} \nabla J^0_{jj'}(\mathbf{R}-\mathbf{R}') \cdot \left(\mathbf{u}_{\mathbf{R}j} - \mathbf{u}_{\mathbf{R}'j'}\right) \hat{\mathbf{S}}_{\mathbf{R}j} \cdot \hat{\mathbf{S}}_{\mathbf{R}'j'} \ . \tag{S29}$$

The atomic displacements can be expressed in terms of phonon modes as

$$\mathbf{u}_{\mathbf{R}j} = \frac{1}{\sqrt{N}} \sum_{\mathbf{k},\lambda} \frac{1}{\sqrt{2M_j \Omega_\lambda(\mathbf{k})}} \mathbf{e}_{j\lambda}(\mathbf{k}) \left(\hat{a}_{\mathbf{q}\lambda} e^{i\mathbf{k}\cdot(\mathbf{R}+\mathbf{r}_j)} + \hat{a}^\dagger_{-\mathbf{k}\lambda} e^{-i\mathbf{k}\cdot(\mathbf{R}+\mathbf{r}_j)} \right) , \tag{S30}$$

where $\hat{a}_{\mathbf{k}\lambda}$ ($\hat{a}^\dagger_{\mathbf{k}\lambda}$) are phonon annihilation (creation) operators for mode λ with momentum $\mathbf{k}$, frequency $\Omega_\lambda(\mathbf{k})$, polarization vector $\mathbf{e}_{j\lambda}(\mathbf{k})$, and mass M_j of atom j in the unit cell. Next, we express the spin operators in Eq. (S29) in terms of magnon operators using the Holstein-Primakoff transformation and the Bogoliubov transformation (S6). Collecting all terms that are second order in magnon operators and first order in phonon operators, we obtain two distinct magnon-phonon interaction processes: (i) normal scattering (magnon number-conserving), where a magnon scatters from momentum $\mathbf{q}$ to $\mathbf{q}+\mathbf{k}$ by absorbing or emitting a phonon with momentum $\mathbf{k}$, and (ii) anomalous scattering (magnon pair creation/annihilation), where two magnons with momenta $\mathbf{q}$ and $-\mathbf{q}+\mathbf{k}$ are created or annihilated by absorbing or emitting a phonon with momentum $\mathbf{k}$. The respective interaction Hamiltonians read as

$$\hat{H}^{\text{normal}}_{\text{mag-ph}} = \frac{1}{\sqrt{N}} \sum_{\mathbf{q},\mathbf{k}} \sum_{\nu} \sum_{\lambda} \frac{g^{\text{normal}}_{\nu\lambda}(\mathbf{q},\mathbf{k})}{\sqrt{2M\Omega_\lambda(\mathbf{k})}} \left(\hat{a}_{\mathbf{k}\lambda} + \hat{a}^\dagger_{-\mathbf{k}\lambda} \right) \hat{\alpha}^\dagger_{\mathbf{q}+\mathbf{k}\nu} \hat{\alpha}_{\mathbf{q}\nu} + \text{h.c.} , \tag{S31}$$

$$\hat{H}^{\text{anomalous}}_{\text{mag-ph}} = \frac{1}{\sqrt{N}} \sum_{\mathbf{q},\mathbf{k}} \sum_{\nu} \sum_{\lambda} \frac{g^{\text{anomalous}}_{\nu\lambda}(\mathbf{q},\mathbf{k})}{\sqrt{2M\Omega_\lambda(\mathbf{k})}} \hat{a}^\dagger_{\mathbf{k}\lambda} \hat{\alpha}_{\mathbf{q}+\mathbf{k}\nu} \hat{\alpha}_{-\mathbf{q}\nu} + \text{h.c.} . \tag{S32}$$

The calculation of the magnon-phonon coupling constants $g^{\text{normal}}_{\nu\lambda}(\mathbf{q},\mathbf{k})$ and $g^{\text{anomalous}}_{\nu\lambda}(\mathbf{q},\mathbf{k})$ involves lengthy expressions depending on the exchange gradients $\nabla J^0_{jj'}(\mathbf{R}-\mathbf{R}')$, the Bogoliubov transformation coefficients, and the phonon polarization vectors. We concentrate here on the phase space structure of magnon-phonon scattering and simplify the coupling constants by (i) replacing $\mathbf{e}_{j\lambda}(\mathbf{k})\cdot\nabla J^0_{jj'}(\mathbf{R}-\mathbf{R}') \to c^x$, where c^x (x =normal, anomalous) are constants, and (ii) approximating the effective masses M_j by a single average mass M of copper.

Using Fermi's golden rule, we obtain the magnon-phonon scattering term in the QBE (S20) as

$$\begin{aligned} S^{\text{mp,normal}}_\nu[f](\mathbf{q}) = \frac{2\pi}{N} \sum_{\mathbf{k},\lambda} \frac{|g^{\text{normal}}_{\nu\lambda}(\mathbf{q},\mathbf{k})|^2}{2M\Omega_\lambda(\mathbf{k})} \Big\{ & \delta(\omega_\nu(\mathbf{q}+\mathbf{k}) - \omega_\nu(\mathbf{q}) - \Omega_\lambda(\mathbf{k})) \\ & \times \left[\bar{f}_\nu(\mathbf{q}+\mathbf{k}) f_\nu(\mathbf{q}) N_\lambda(\mathbf{k}) - f_\nu(\mathbf{q}+\mathbf{k},t) \bar{f}_\nu(\mathbf{q},t) \bar{N}_\lambda(\mathbf{k}) \right] \\ & + \delta(\omega_\nu(\mathbf{q}+\mathbf{k}) - \omega_\nu(\mathbf{q}) + \Omega_\lambda(\mathbf{k})) \\ & \times \left[\bar{f}_\nu(\mathbf{q}-\mathbf{k},t) f_\nu(\mathbf{q},t) \bar{N}_\lambda(\mathbf{k}) - f_\nu(\mathbf{q}-\mathbf{k},t) \bar{f}_\nu(\mathbf{q},t) N_\lambda(\mathbf{k}) \right] \Big\} , \end{aligned} \tag{S33}$$

where $N_\lambda(\mathbf{q})$ is the phonon occupation number for mode λ with momentum $\mathbf{q}$, and where $\bar{N}_\lambda(\mathbf{q}) = 1 + N_\lambda(\mathbf{q})$. The anomalous magnon-phonon scattering term reads as

$$S_\nu^{\text{mp,anomalous}}[f](\mathbf{q}) = \frac{2\pi}{N} \sum_{\mathbf{k},\lambda} \frac{|g_{\nu\lambda}^{\text{anomalous}}(\mathbf{q},\mathbf{k})|^2}{2M\Omega_\lambda(\mathbf{k})} \delta(\omega_\nu(\mathbf{q}+\mathbf{k}) + \omega_\nu(-\mathbf{q}) - \Omega_\lambda(\mathbf{k})) \times \left[\bar{f}_\nu(\mathbf{q}+\mathbf{k},t)\bar{f}_\nu(-\mathbf{q},t)N_\lambda(\mathbf{k}) - f_\nu(\mathbf{q}+\mathbf{k},t)f_\nu(-\mathbf{q},t)\bar{N}_\lambda(\mathbf{k})\right] . \tag{S34}$$

The dispersion of the acoustic phonons $\Omega_\lambda(\mathbf{k})$ are estimated from the model

$$\Omega_\lambda(\mathbf{k}) = \left(\sum_{i=1}^{3} C_{i\lambda}^{(0)} \sin^2\left(\frac{\mathbf{k}\cdot\mathbf{a}_i}{2}\right)\right)^{1/2} . \tag{S35}$$

Here, $\mathbf{a}_i$ are the primitive lattice vectors of CuO, and $C_{i\lambda}^{(0)}$ are fitting parameters chosen to reproduce the acoustic phonon velocities along high-symmetry directions from Ref. [19]. Similarly, we approximate the optical phonon modes by

$$\Omega_\lambda(\mathbf{k}) = C_\lambda^{(1)} + \sum_{i=1}^{3} C_{i\lambda}^{(2)} \cos(\mathbf{k}\cdot\mathbf{a}_i) , \tag{S36}$$

where $C_\lambda^{(1)}$ and $C_{i\lambda}^{(2)}$ are fitting parameters chosen to reproduce the optical phonon frequencies reported in ref. [19] at the Γ-point and at the zone boundaries.

With these ingredients, we evaluated Eqs. (S33) and (S34) numerically on a $N = 64 \times 64 \times 64$ $\mathbf{k}$-point grid. We fixed $c^x = 1$ eV/Å for both normal and anomalous magnon-phonon coupling constants. The phonon occupation numbers $N_\lambda(\mathbf{k})$ are defined by the Bose-Einstein distribution at $T = 200\,\mathrm{K}$ (corresponding to a slightly excited distribution with respect to the equilibrium), while we used the steady-state distribution from the magnon-magnon QBE simulation as initial magnon distribution. For simplicity, we only considered the initial scattering rate $S_\nu^{\text{mp}}[f](\mathbf{q}, t = 0)$ due to magnon-phonon interactions, from which we can estimate the magnon-phonon scattering rate as $\Gamma_\nu(\mathbf{q}) = S_\nu^{\text{mp}}[f](\mathbf{q}, t = 0)/f_\nu(\mathbf{q}, t = 0)$.

The results for the acoustic phonons and the lowest-energy optical phonons are shown in Fig. S12. While normal magnon-phonon scattering is an order of magnitude faster than anomalous scattering, the normal process cannot reduce the total magnon population. There are redistribution processes in momentum space due to the different anisotropy of the magnon and the phonon dispersion, mostly at momenta away from the magnetic Γ-point. Close to Γ, however, the normal

magnon-phonon scattering rate is strongly suppressed due to the lack of phase space for energy- and momentum conservation. The anomalous magnon-phonon scattering rate is purely negative and is generally smaller in magnitude. It is most effective close to the magnetic Γ-point, where two low-energy magnons can be annihilated by emitting a phonon. We conclude that only the anomalous magnon-phonon scattering process can contribute to the magnetic recovery dynamics observed in the experiments. The small magnitude of the anomalous magnon-phonon scattering rate, originating from the mismatch of magnon and phonon dispersion, is consistent with the experimentally observed slow recovery dynamics.

We also evaluated the magnon-phonon scattering rates for acoustic phonons. The corresponding scattering rates are orders of magnitude smaller than those for the lowest-energy optical phonons, due to the small energy scale of acoustic phonons compared to magnons.

Supplementary Note 11 — Phonon dissipation process

Figure S13 shows a long timescale comparison of tr-NRDS intensities between the AFM phase (190 K) and the PM phase (300 K), demonstrating the relaxation process of phonons at a microsecond timescale. While the time constant is ~0.1 μs in the PM phase, it is ~0.7 μs in the AFM phase. Although the slow energy transfer process from magnons via the anomalous scattering creates additional phonons in the AFM phase, the phonon diffusion efficiency should be substantially higher at low temperatures due to a larger phonon mean path length, resulting in a larger phonon thermal conductivity[20]. The temperature dependence is not explained by the previously proposed magnon scenario, either [20]. Thus, it is indicated that the electronic thermal conductivity significantly contributes to the energy dissipation process, likely due to oxygen excess[21] and Cu vacancies[22].

Supplementary Note 12 — Additional evidence of magnon density redistribution

RDS signals can exhibit more complex time evolution than the simple phenomenological model with two exponential functions due to the magnon density redistribution processes that depend on both timescale and momentum point. Figure S14 represents a time trace of resonant X-ray scattering intensity measured at (0.48, 0.01, −0.52). The initial drop is due to ultrafast demagnetization

causing RXD intensity reduction, followed by clear RDS upturn signals. However, the subsequent evolution is non-monotonic and exhibits a maximum around 5 ps and plateaus after ~10 ps until further relaxation occurs via the anomalous scattering. These intricate dynamics further support the interpretation of magnon density redistribution within the acoustic magnon branch, in addition to possible contributions from optical magnon decay and the magnon-number conserved scattering (main text).

Supplementary Figures

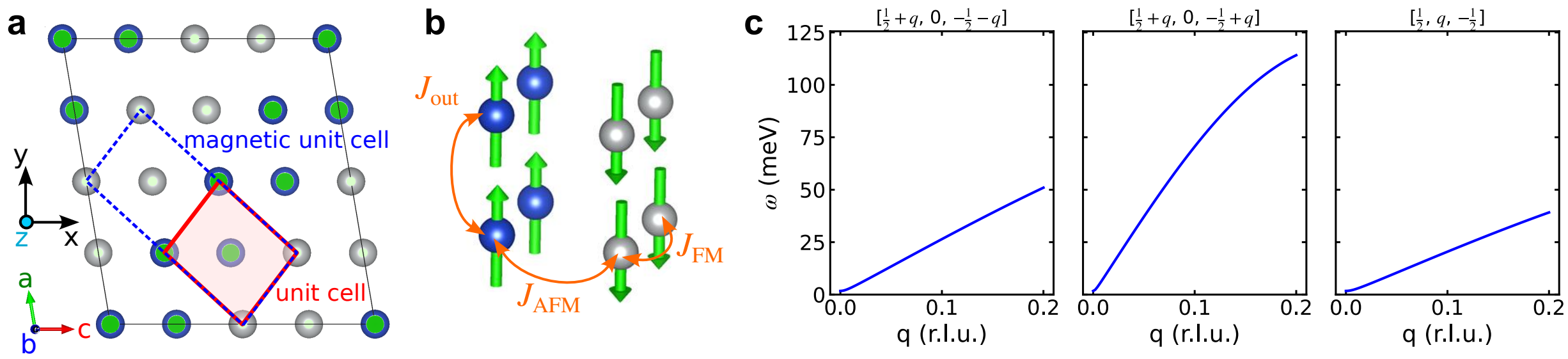

Figure S1: **Model and magnon dispersion. a**, Simplified (magnetic) unit cell. The spin orientation is described in the xyz coordinate system. **b**, Model unit cell with next-nearest-neighbor exchange couplings, drawn using VESTA [23]. **c**, Magnon dispersion of the model spin Hamiltonian. The dispersion was computed along three directions in the Brillouin zone corresponding to the simplified unit cell and convert to (approximately) to the reciprocal path directions used in the experiments.

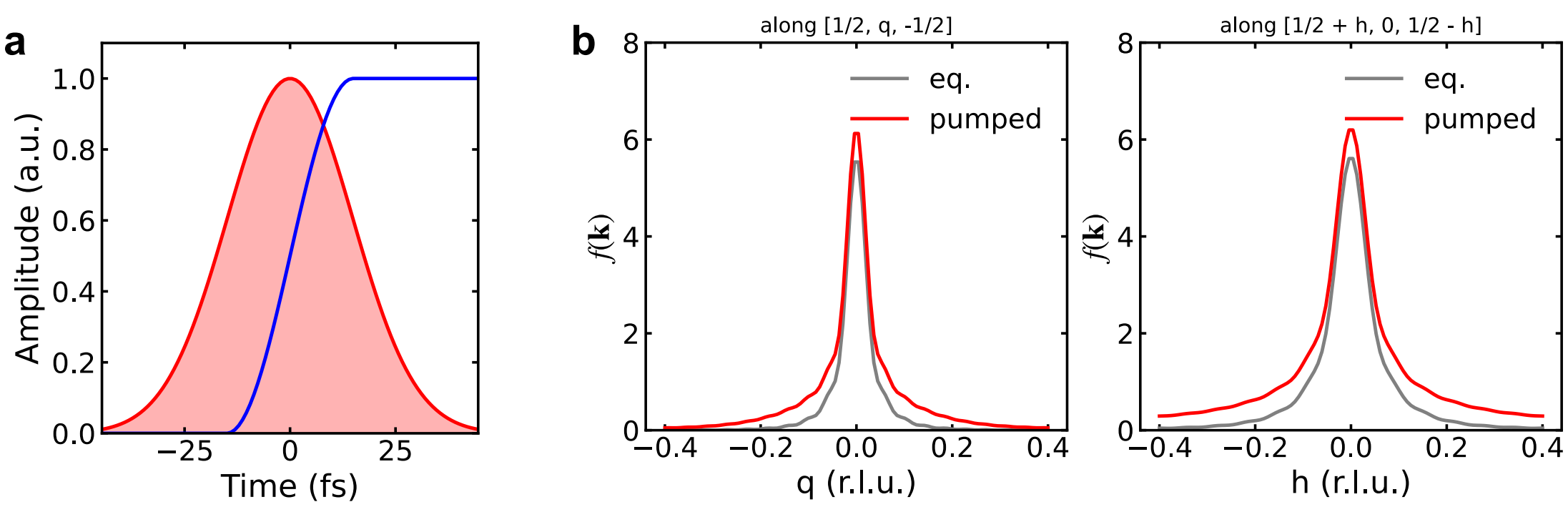

Figure S2: **Photoinduced magnon occupation. a**, Sketch of the envelope of the pump pulse and the approximated $B_0\Delta n(t)$. **b**, Magnon occupation $f(\mathbf{k})$ along the reciprocal axes in equilibrium at $T = 190$ K, and immediately after photoexcitation. The wiggles in the equilibrium distribution are due to Fourier interpolation artifacts.

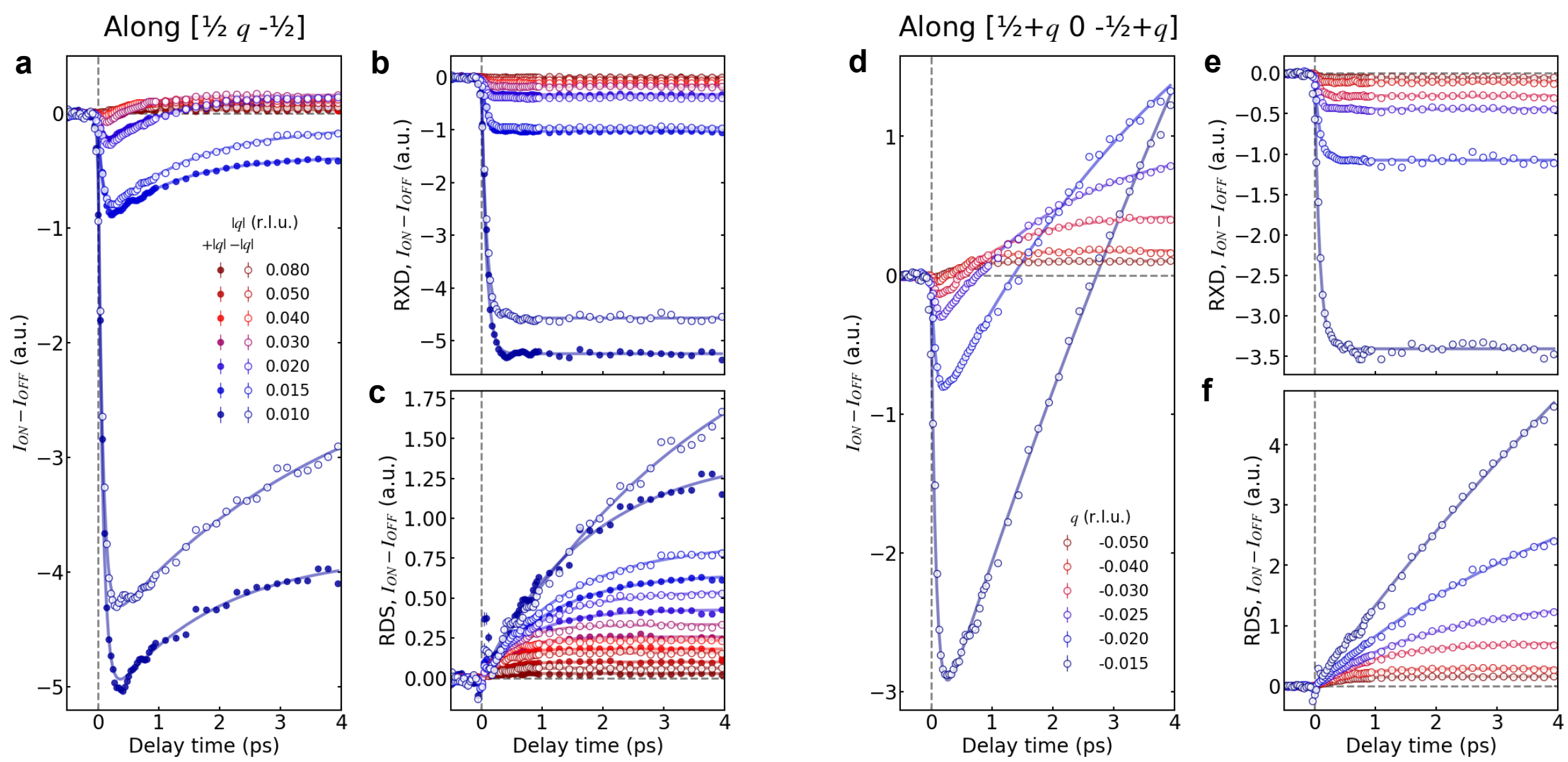

Figure S3: **Deconvolution of time-resolved resonant X-ray scattering intensities into RXD and RDS components.** **a** is identical as Fig. 3**a**. Momentum dependence of time-dependent resonant X-ray scattering intensities around the D point (see main text) (**a** and **d**) was deconvoluted into two exponential terms representing the RXD (**b** and **e**) and RDS (**c** and **f**) components. Solid curves represent fits using two exponential terms to separate the RXD drop and RDS upturn components. Filled (open) points indicate data measured at positive (negative) q.

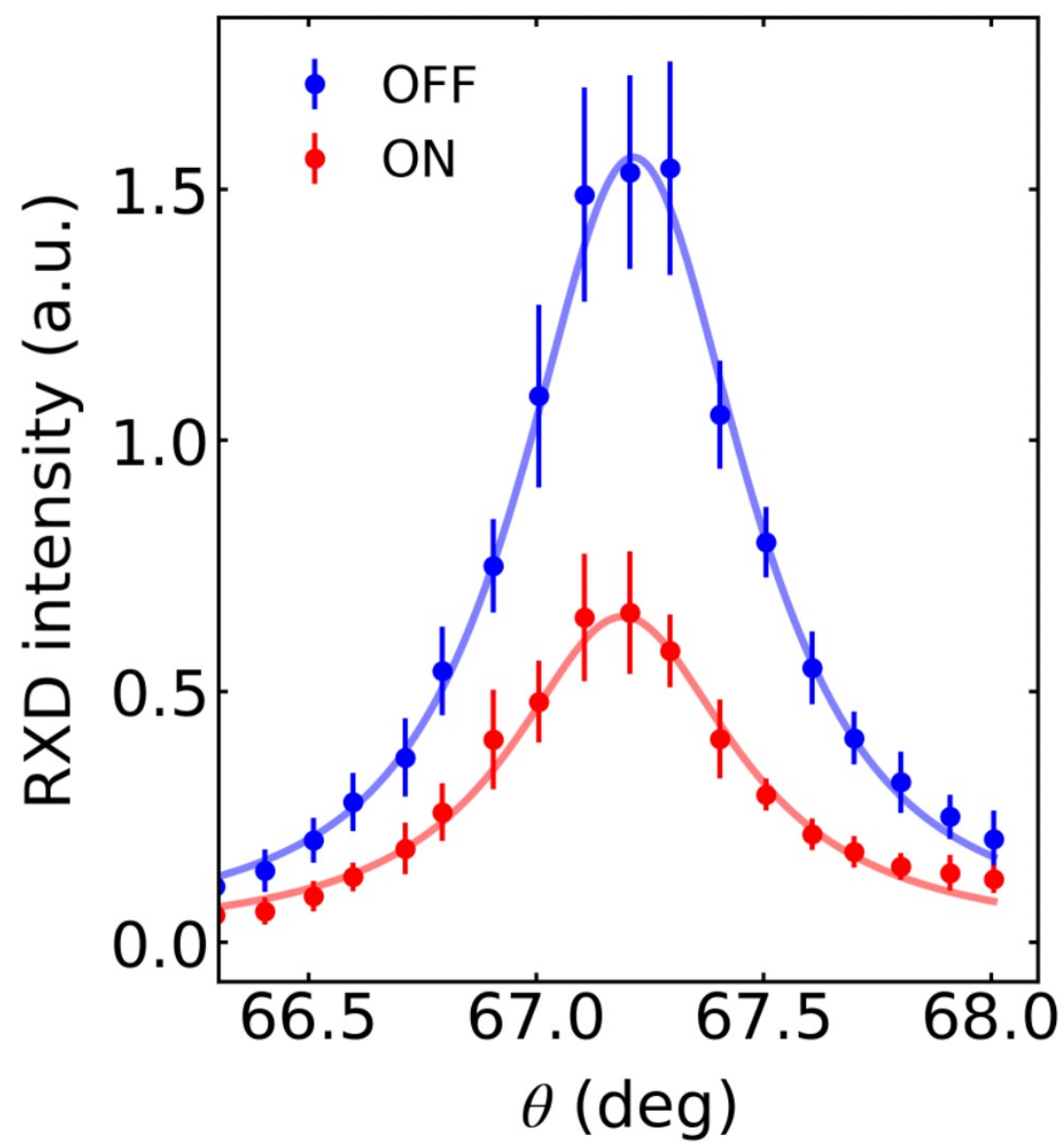

Figure S4: **Constant correlation length before and after the optical excitation.** Rocking curve with (red) and without (blue) the optical excitation, measured at a delay time of 4.7 ps.

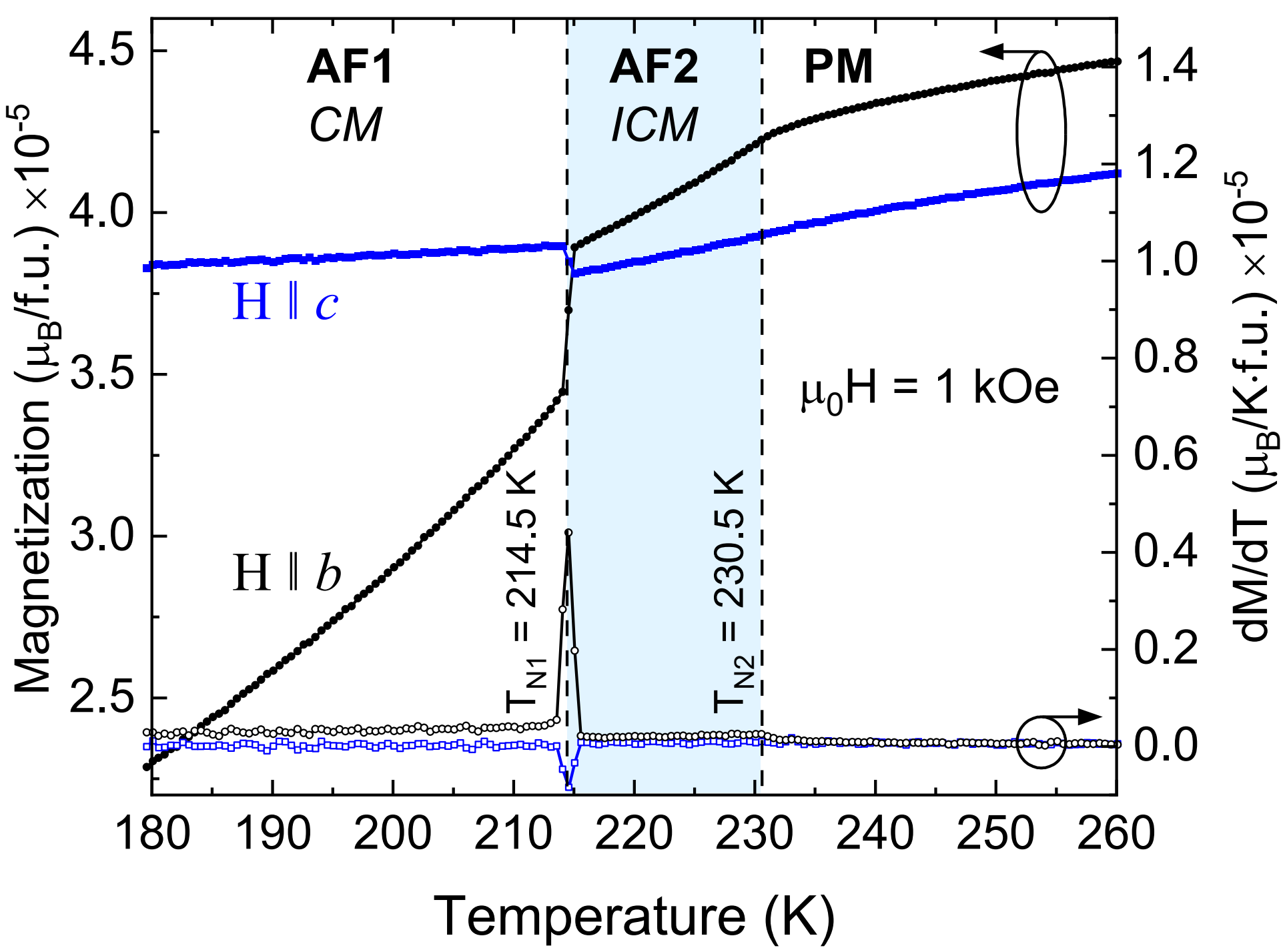

Figure S5: **Temperature dependence of magnetic susceptibility of the CuO single crystal sample.** ICM and CM represent the incommensurate magnetic phase and commensurate magnetic phase, respectively.

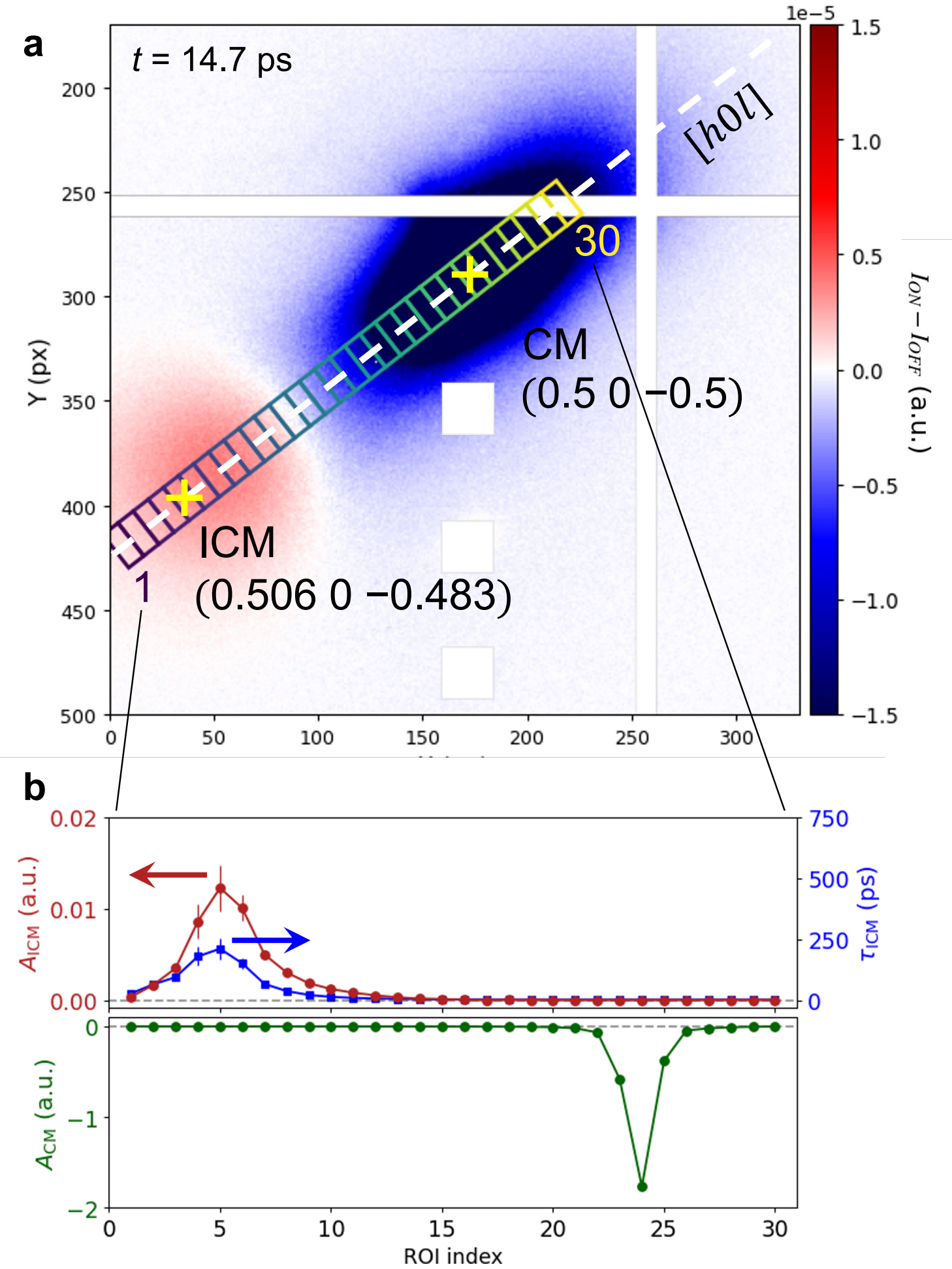

Figure S6: **Transient appearance of incommensurate order at high excitation regime. a**, Difference detector image ($I_{ON} - I_{OFF}$) of resonant X-ray scattering at a pump-probe delay of t = 14.7 ps. The blue (negative) region corresponds to the suppressed CM Bragg peak (1/2 0 −1/2); the red (positive) region indicates the transient appearance of the ICM peak. Colored rectangles mark 30 regions of interest (ROIs) along the direction connecting the ICM and CM peak positions; white areas are masked detector defects. **b**, Fit parameters extracted for each ROI by fitting the time-dependent signal with a double-exponential model analogous to Eq. S11. Top: amplitude A_{ICM} (red, left axis) and time constant τ_{ICM} (blue, right axis) of the component associated with the growth of the ICM order. Bottom: amplitude A_{CM} (green) of the CM peak suppression component. The two distinct dynamics develop symmetrically about the CM and the emerging ICM positions in reciprocal space.

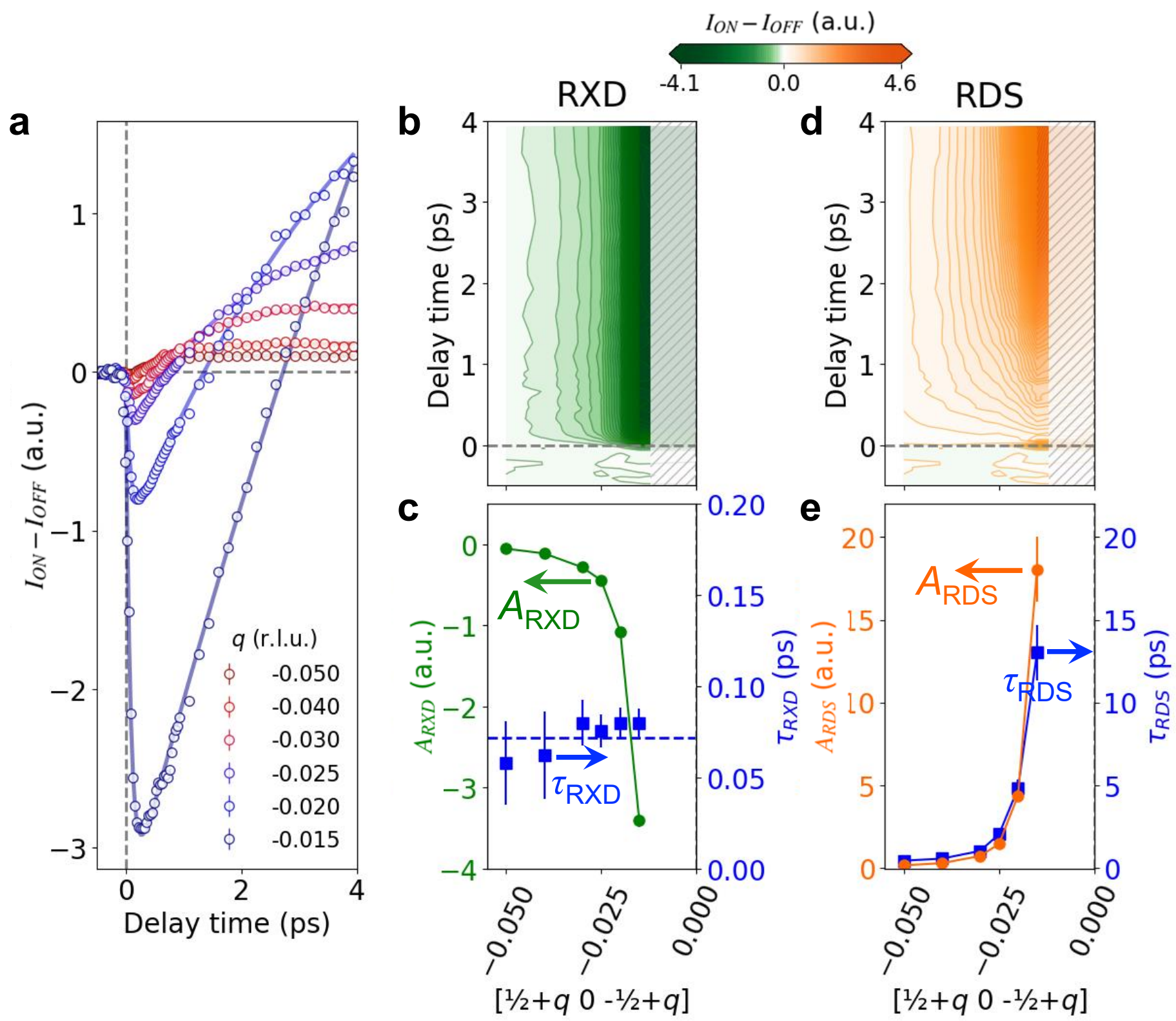

Figure S7: **Momentum dependence of time-resolved resonant diffuse scattering intensities along [h 0 h].** **a**, Time traces of RDS signals at momentum points around the D point (see main text) along $[h\ 0\ h]$. Solid curves represent fits using two exponential terms to separate the RXD drop and RDS upturn components. **b,d**, Momentum-delay time contour plots of the RXD (**b**) and RDS (**d**) components derived from the time traces in **a**. **c,e**, Momentum dependence of RXD-drop (**c**) and RDS-upturn (**e**) amplitudes and their time constants extracted from fits to the data in **a**. Error bars represent standard error in **a** and are from the fit in **c** and **e**.

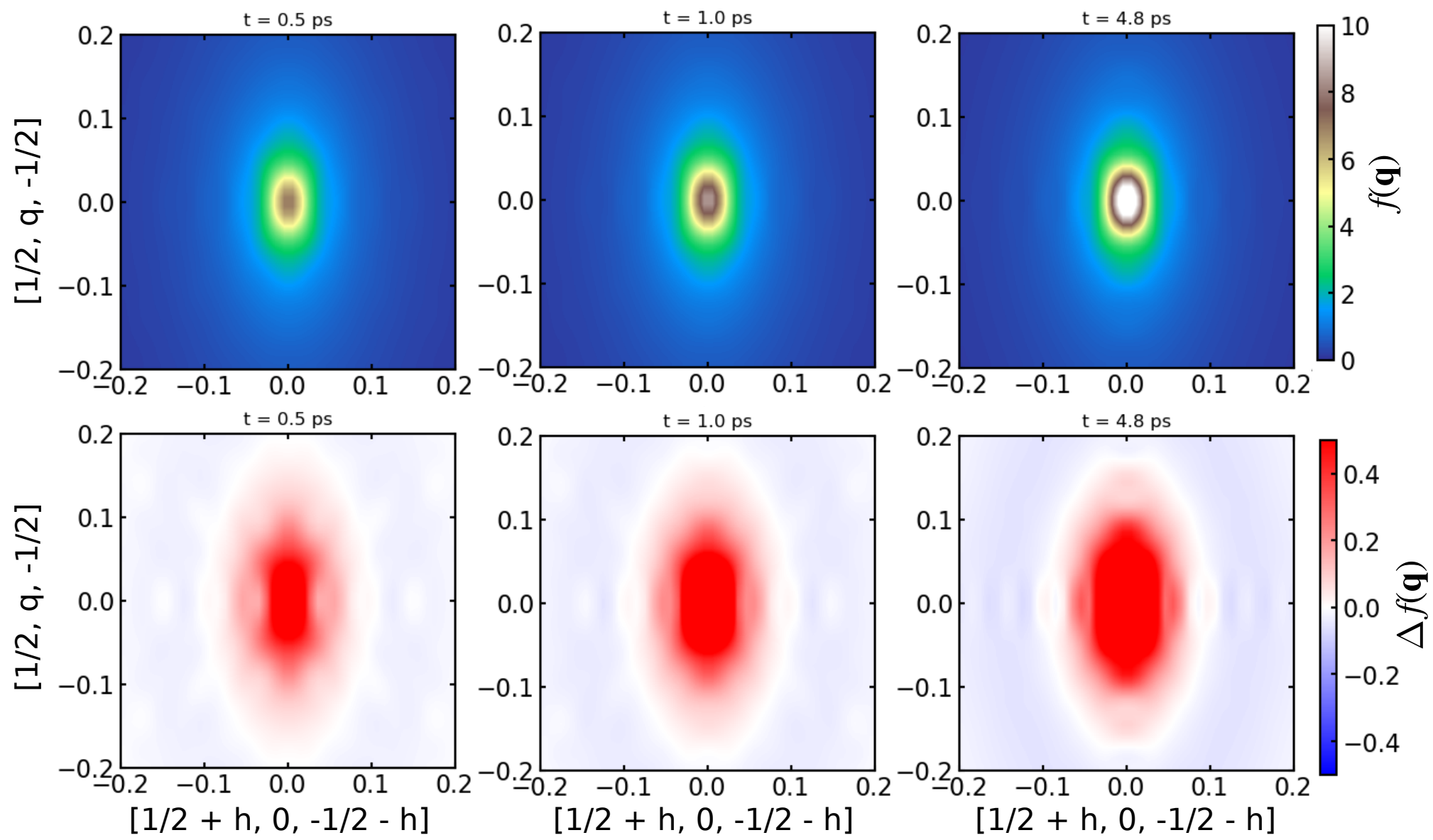

Figure S8: **Magnon thermalization dynamics.** Snapshots of the magnon distribution $f(\mathbf{q},t)$ (top row) and difference $\Delta f(\mathbf{q},t) = f(\mathbf{q},t) - f(\mathbf{q},0)$ (bottom row) along the reciprocal-space directions equivalent to the experiments for different time steps.

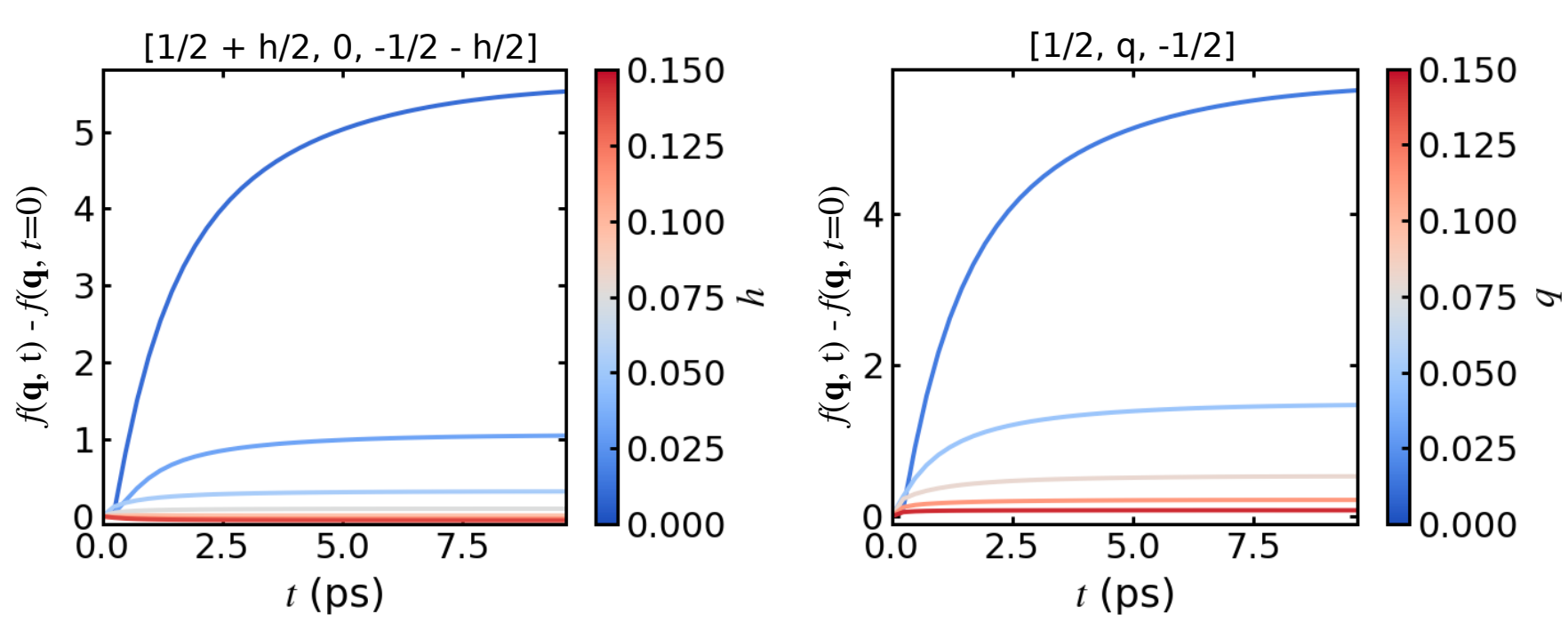

Figure S9: **Momentum-dependent magnon occupation dynamics.** Time evolution of the change of magnon occupation $\Delta f(\mathbf{q},t) = f(\mathbf{q},t) - f(\mathbf{q},0)$ for selected $\mathbf{q}$-points, indicated by the color coding.

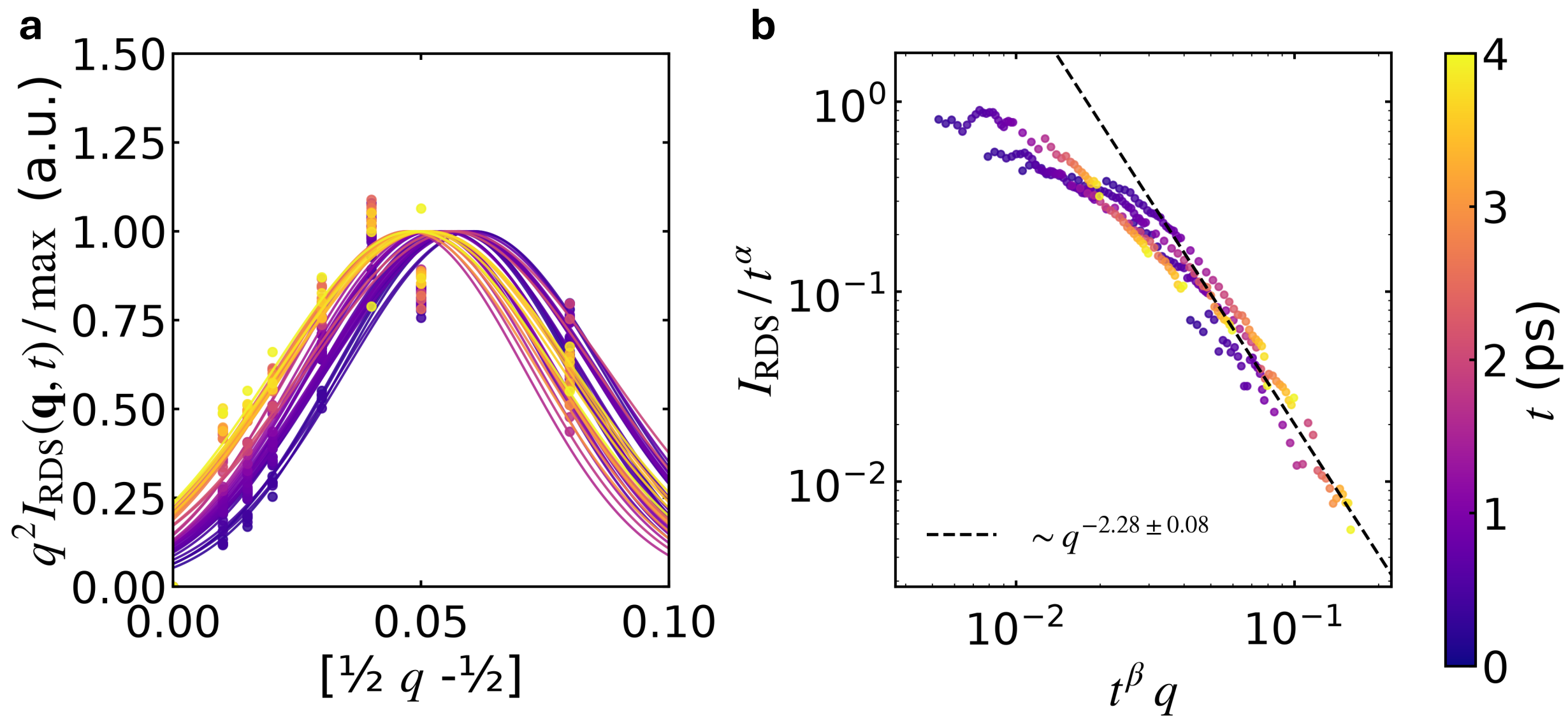

Figure S10: **Self-similarity and power-law scaling of rescaled tr-RDS signals a**, $q^2 I_{\mathrm{RDS}}$ plotted at different delays t. I_{RDS} signals are taken from Fig. 3**e**. Solid curves are fits to Gaussian peaks as guides to the eye, indicating self-similarities across different delays. The vertical axis is normalized to the maximum value for each delay. **b** Rescaled tr-RDS signals with $\alpha = 1.0$ and $\beta = 0.5$, following Ref. [18]. A linear fit yields a power-law exponent of 2.28±0.08, close to the nominal value of 2.33 for an inverse particle cascade in wave turbulence, in which particles flow toward lower momentum.

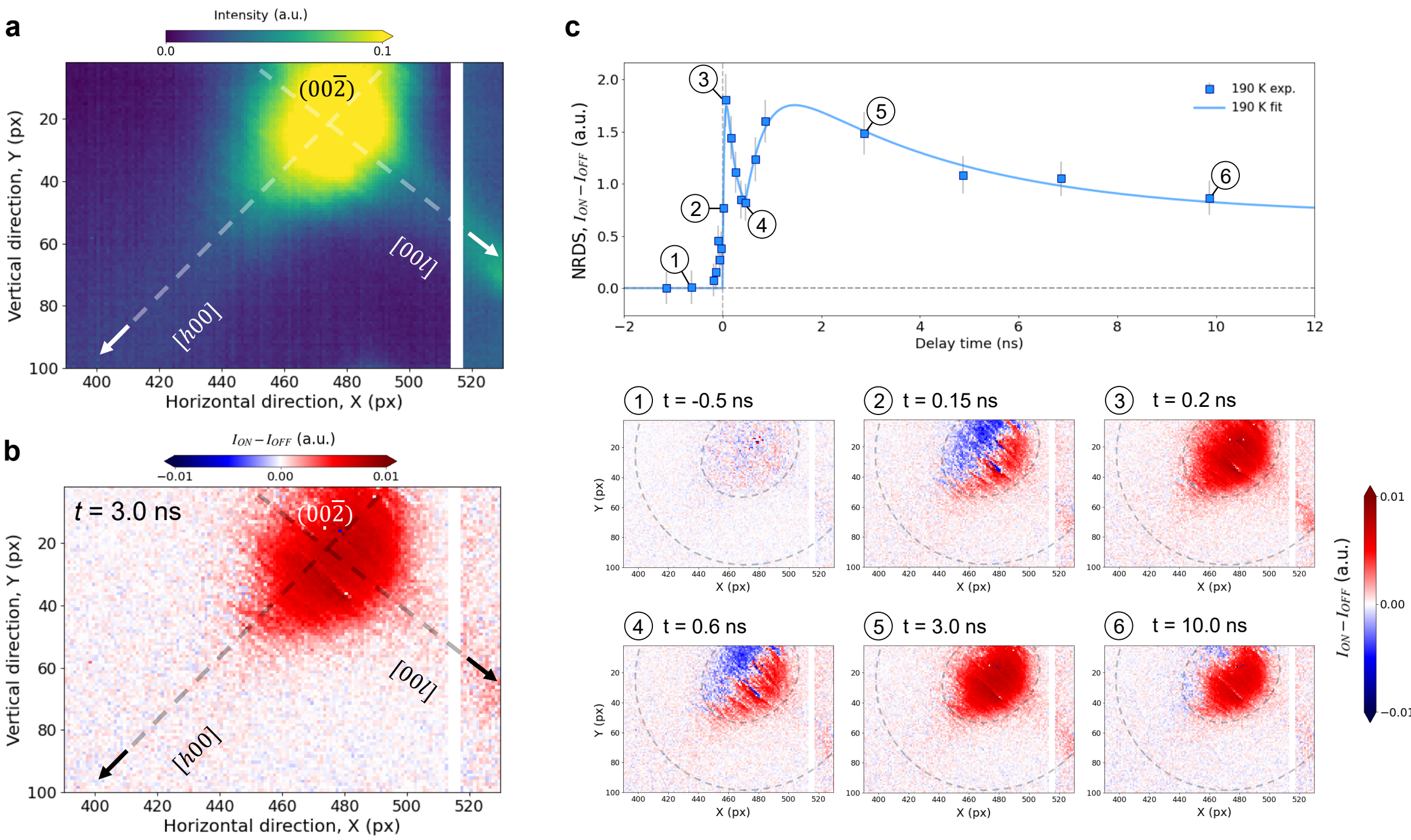

Figure S11: **Tr-NRDS signals on a two-dimensional detector. a**, (0 0 −2) diffraction pattern in equilibrium. The color scale is saturated at the high-intensity side to enhance the visibility of weak features. Streaks along [0 0 l] and [h 0 0] indicate NRDS by pre-existing phonons due to thermal energy and static disorder. **b**, Pump-induced change in X-ray scattering intensities at 3 ns after the photoexcitation. **c**, Time trace of tr-NRDS intensities, the same as the blue plot in Fig. 4**d**. Bottom panels display the pump-induced change in two-dimensional X-ray scattering intensities at respective delay times indicated by the numbers in the top panel.

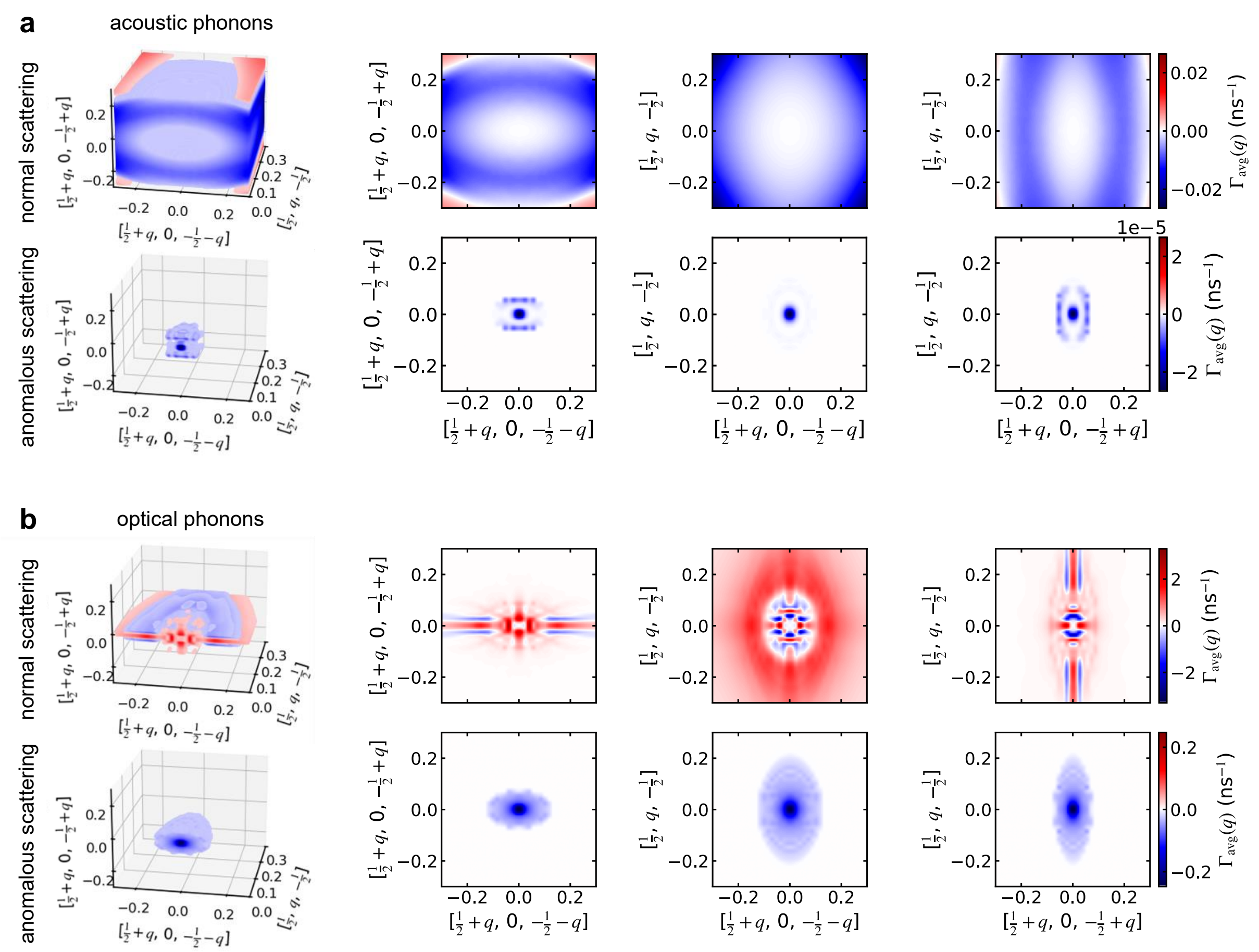

Figure S12: **Magnon-phonon scattering rate.** Mode-averaged magnon-phonon scattering rate for **a**, the acoustic phonons and **b**, the lowest-energy optical phonons in three-dimensional momentum space and along high-symmetry planes. Top row: normal scattering, bottom row: anomalous scattering.

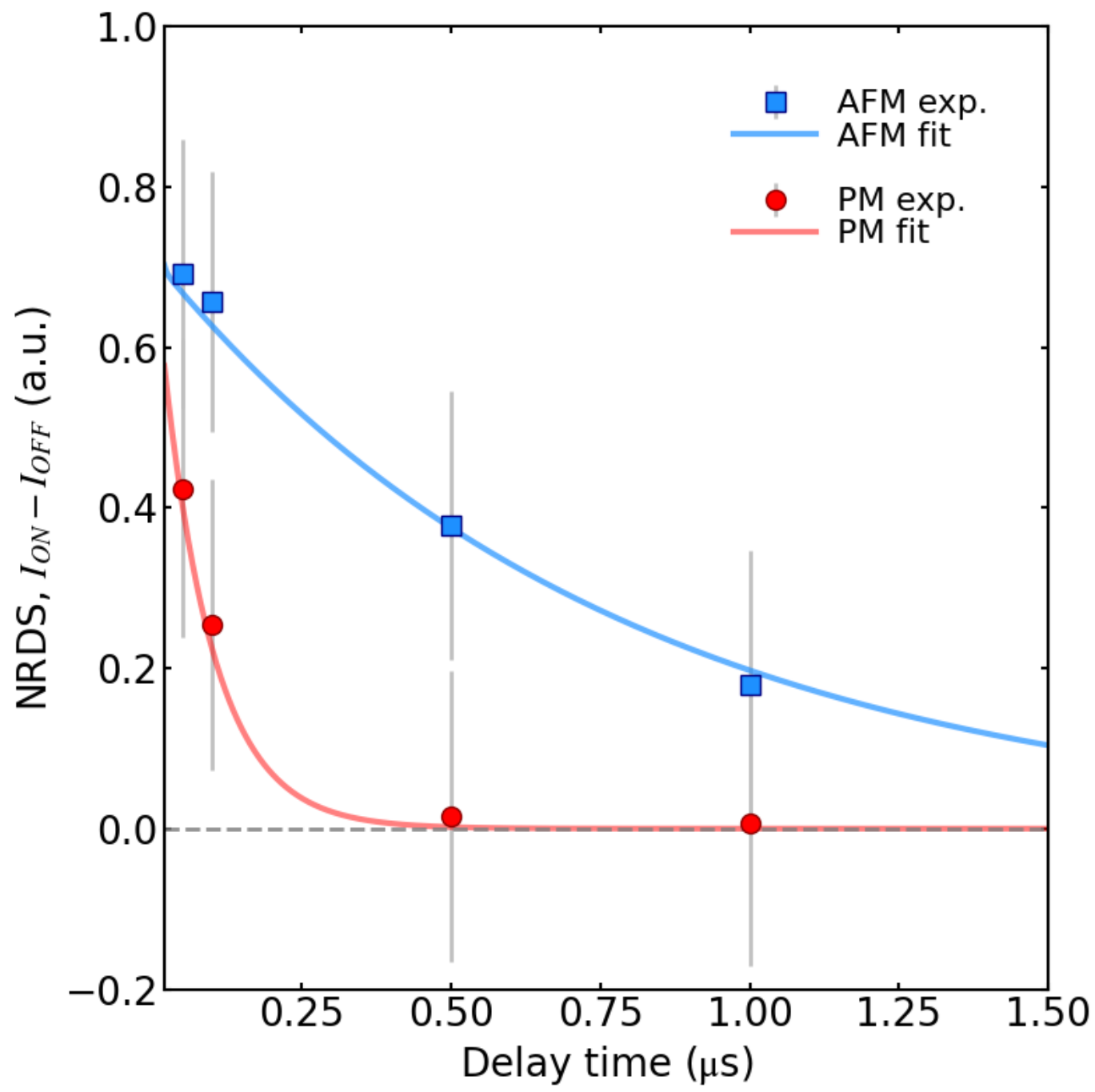

Figure S13: **Long timescale comparison of tr-NRDS intensities between the AFM phase and PM phase.**

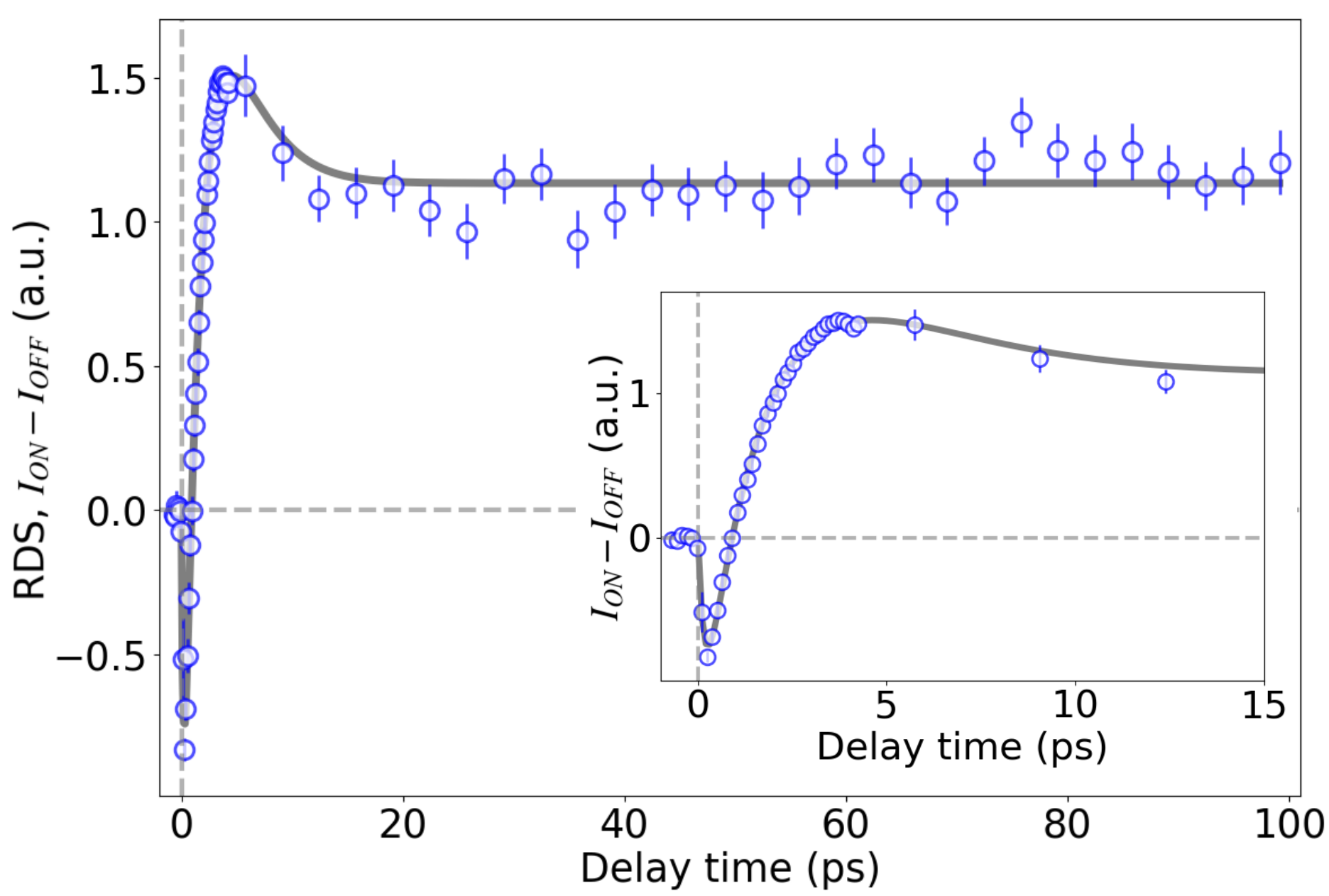

Figure S14: **Time trace of RDS at (0.48, 0.01, −0.52).** Errors are standard errors.

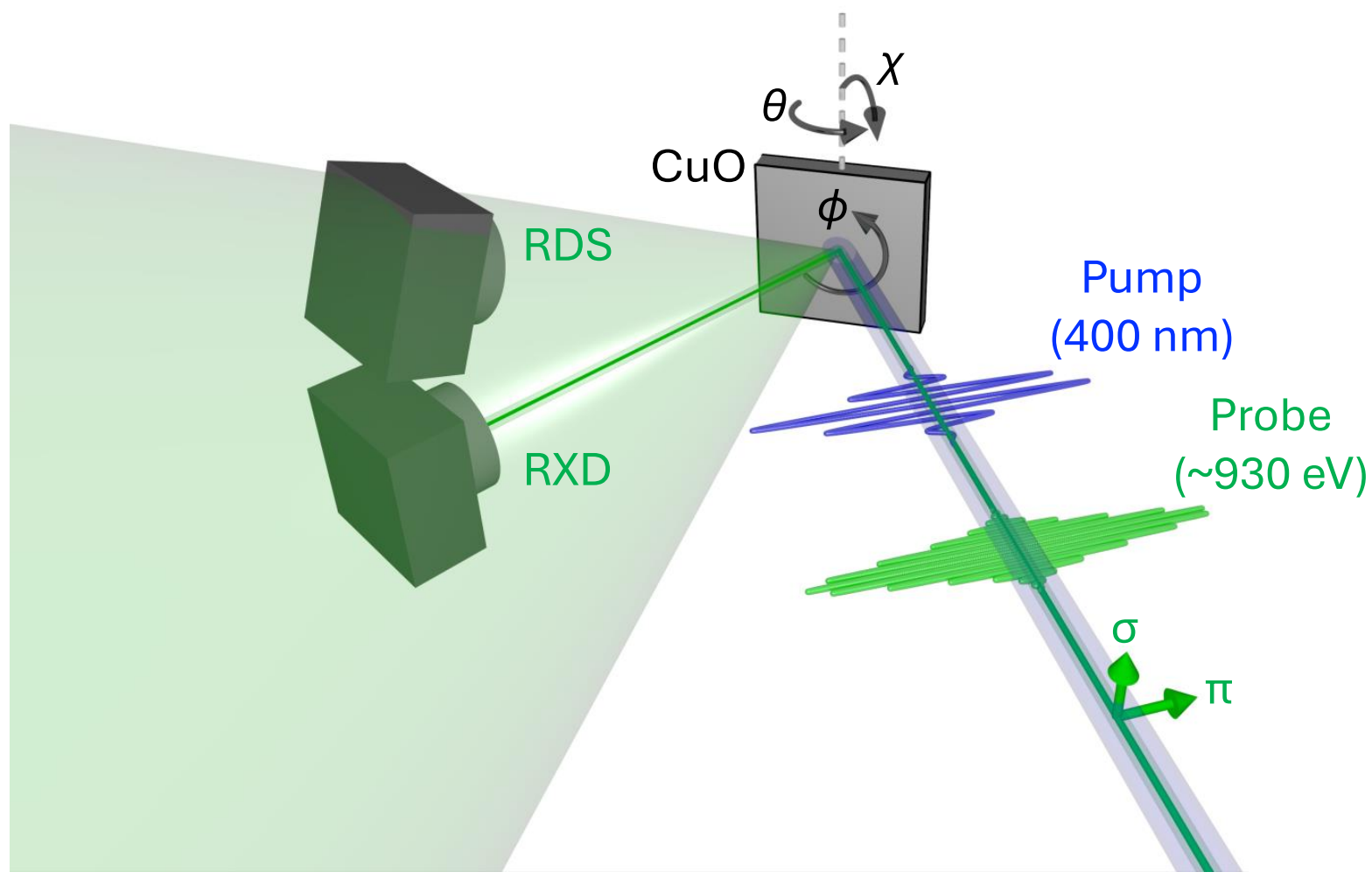

Figure S15: **Schematic of experimental setup for tr-RXD and tr-RDS measurements.**

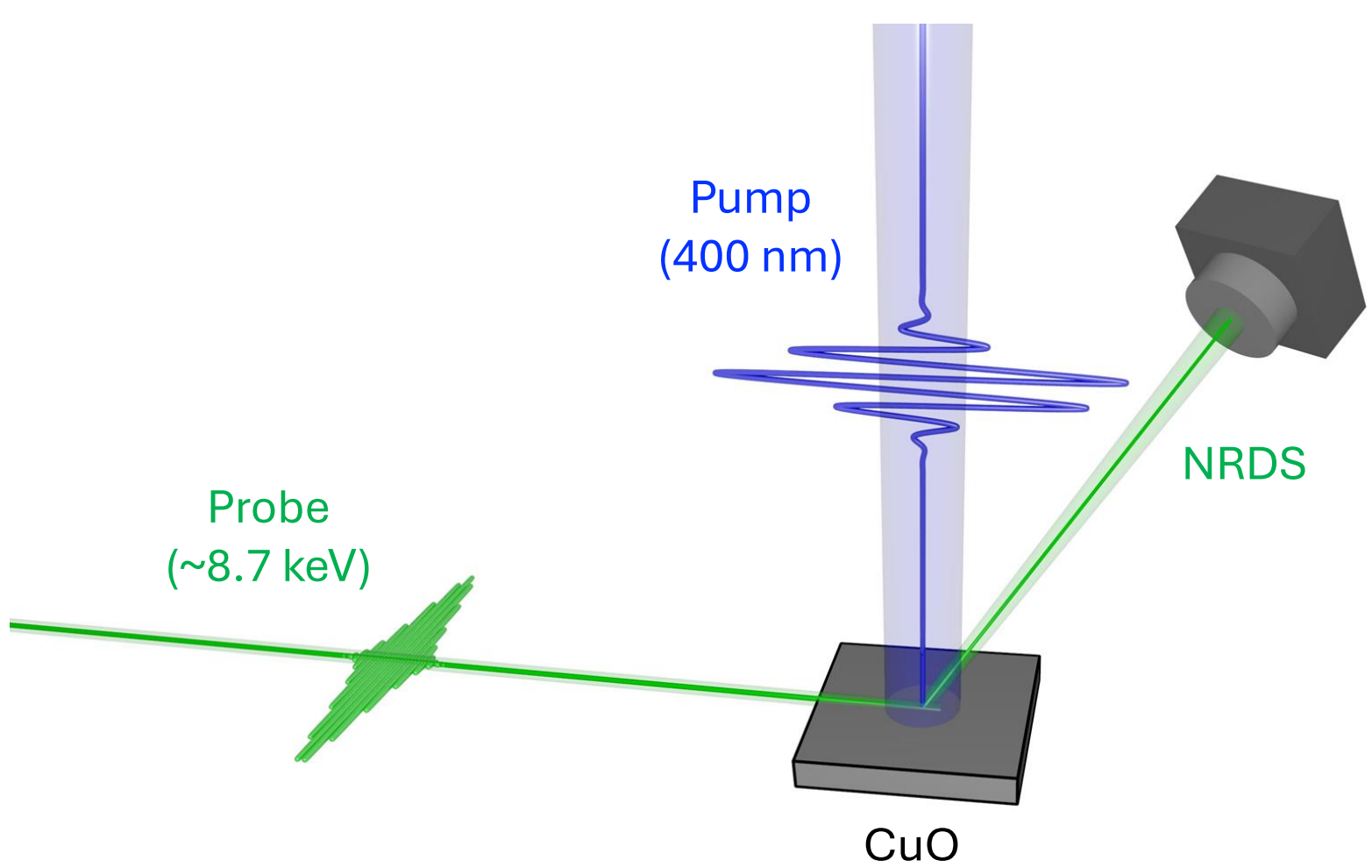

Figure S16: **Schematic of experimental setup for tr-NRDS measurements.**

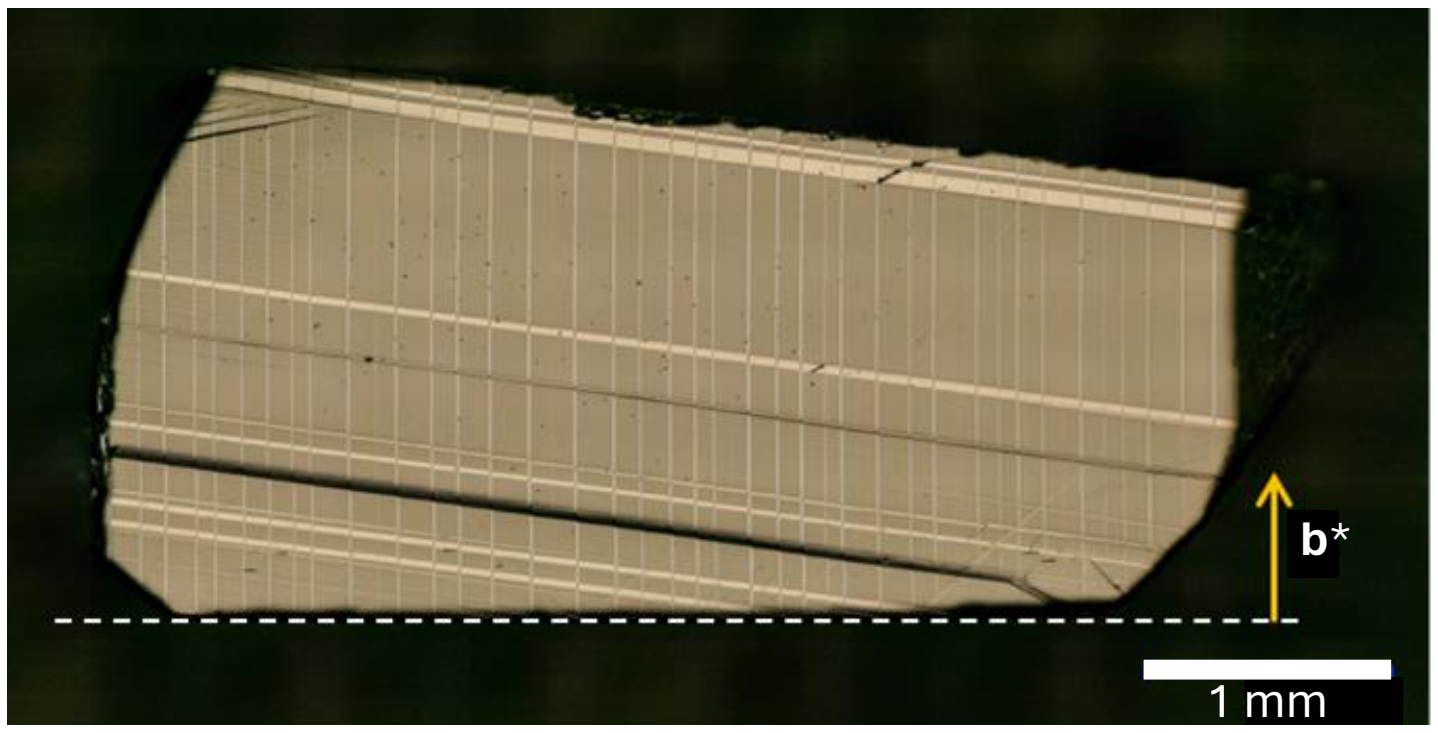

Figure S17: **Polarized microscope image on the (1 0 −1) surface of the CuO single crystal sample.** Bright regions represent differently oriented twin domains.

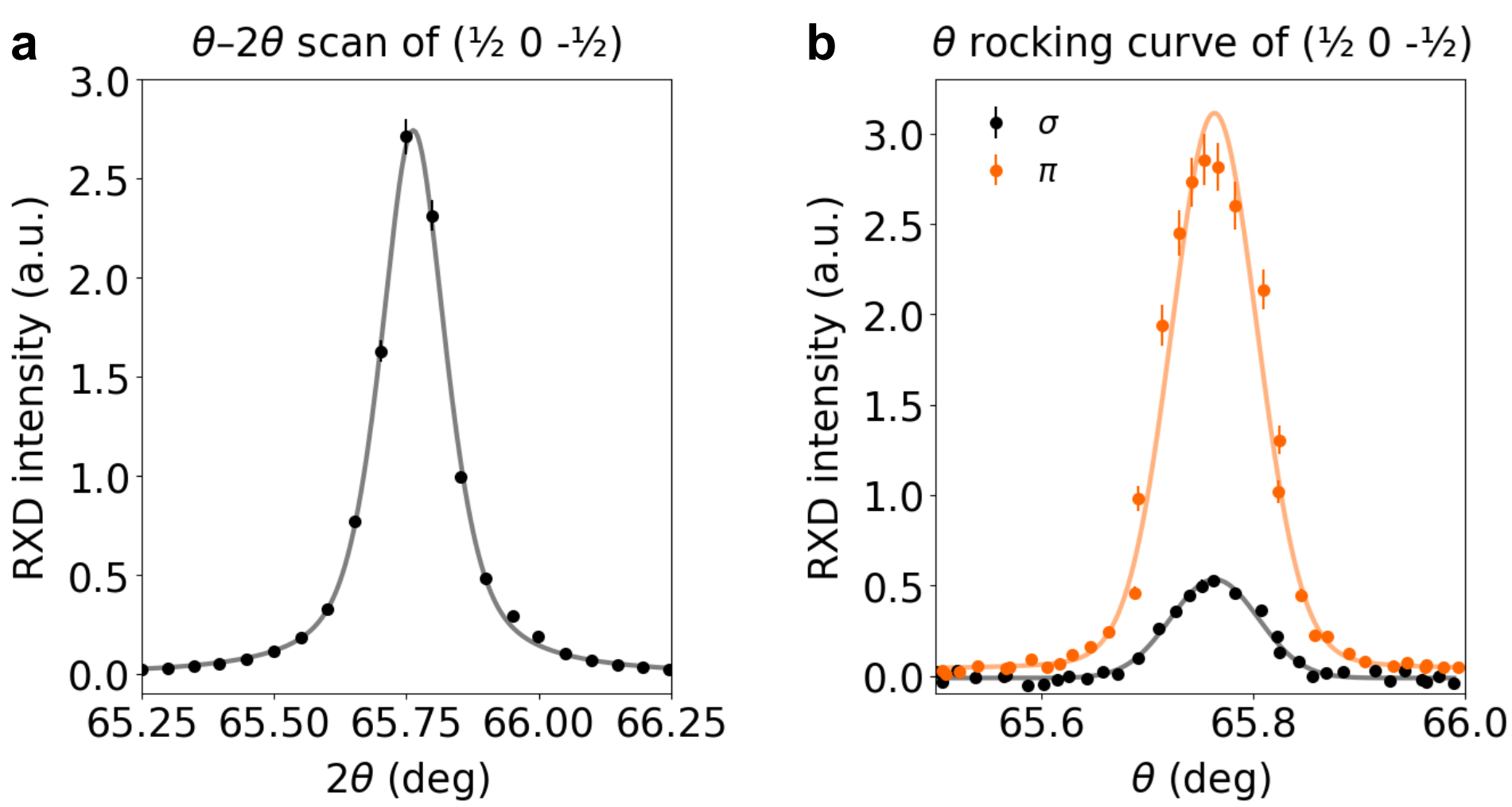

Figure S18: **Diffraction profile around the (1/2 0 −1/2) magnetic Bragg reflection. a**, A $\theta - 2\theta$ profile and **b**, rocking curve profiles around the (1/2 0 −1/2) magnetic Bragg reflection. Polarization dependence is also shown in **b**. Solid curves represent Gaussian fit. Error bars are standard errors.

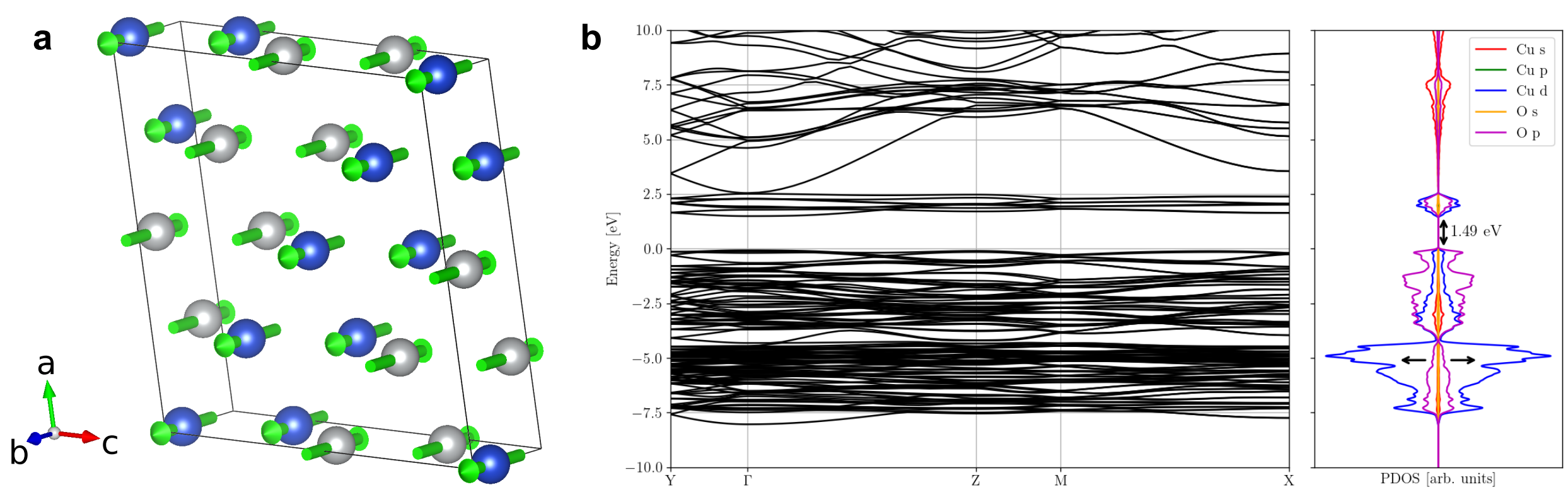

Figure S19: **Electronic structure of CuO. a**, Antiferromagnetic unit cell of CuO, rotated to align the spin direction with the z direction. The magnetic moments are indicated by arrows. The structure was drawn using VESTA [23]. **b**, Band structure and corresponding partial density of states (PDOS).

References

[1] Gillmeister, K. *et al.* Ultrafast coupled charge and spin dynamics in strongly correlated NiO. *Nature Communications* **11**, 4095 (2020).

[2] Golež, D., Eckstein, M. & Werner, P. Multiband nonequilibrium G W + EDMFT formalism for correlated insulators. *Physical Review B* **100**, 235117 (2019).

[3] Huang, T.-S., Baldwin, C. L., Hafezi, M. & Galitski, V. Spin-mediated Mott excitons. *Physical Review B* **107**, 075111 (2023).

[4] Aïn, M., Reichardt, W., Hennion, B., Pepy, G. & Wanklyn, B. M. Magnetic excitations in cuo. *Physica C: Superconductivity and its Applications* **162–164**, 1279–1280 (1989).

[5] Giovannetti, G. *et al.* High-T_c ferroelectricity emerging from magnetic degeneracy in cupric oxide. *Physical Review Letters* **106**, 026401 (2011).

[6] Toth, S. & Lake, B. Linear spin wave theory for single-Q incommensurate magnetic structures. *Journal of Physics: Condensed Matter* **27**, 166002 (2015).

[7] Mazzone, D. G. *et al.* Laser-induced transient magnons in $sr_3ir_2o_7$ throughout the brillouin zone. *Proceedings of the National Academy of Sciences* **118**, e2103696118 (2021).

[8] Perfetti, L. *et al.* Ultrafasst electron relaxation in superconducting $bi_2sr_2cacu_2o_{8+\delta}$ by time-resolved photoelectron spectroscopy. *Physical Review Letters* **99**, 197001 (2007).

[9] Novelli, F. *et al.* Witnessing the formation and relaxation of dressed quasi-particles in a strongly correlated electron system. *Nature Communications* **5**, 5112 (2014).

[10] Baykusheva, D. R. *et al.* Ultrafast renormalization of the on-site coulomb repulsion in a cuprate superconductor. *Physical Review X* **12**, 011013 (2022).

[11] Johnson, S. L. *et al.* Femtosecond dynamics of the collinear-to-spiral antiferromagnetic phase transition in cuo. *Physical Review Letters* **108**, 037203 (2012).

[12] Gmelin, E., Köbler, U., Brill, W., Chattopadhyay, T. & Sastry, S. Magnetic specific heat and susceptibility of cupric oxide (cuo) single crystals. *Bulletin of Materials Science* **14**, 117–123 (1991).

[13] Ament, L. J. P., van Veenendaal, M., Devereaux, T. P., Hill, J. P. & van den Brink, J. Resonant inelastic x-ray scattering studies of elementary excitations. *Reviews of Modern Physics* **83**, 705–767 (2011).

[14] Haverkort, M. W. Theory of Resonant Inelastic X-Ray Scattering by Collective Magnetic Excitations. *Physical Review Letters* **105**, 167404 (2010).

[15] Newton, R. G. Optical theorem and beyond. *American Journal of Physics* **44**, 639–642 (1976).

[16] Haverkort, M. W., Hollmann, N., Krug, I. P. & Tanaka, A. Symmetry analysis of magneto-optical effects: The case of x-ray diffraction and x-ray absorption at the transition metal $L_{2,3}$ edge. *Physical Review B* **82**, 094403 (2010).

[17] Kalthoff, M. H., Kennes, D. M., Millis, A. J. & Sentef, M. A. Nonequilibrium phase transition in a driven-dissipative quantum antiferromagnet. *Physical Review Research* **4**, 023115 (2022).

[18] Li, Z., Glorioso, P. & Rodriguez-Nieva, J. F. Far-from-equilibrium universality in two-dimensional heisenberg antiferromagnets. *Physical Review B* **108**, 144303 (2023).

[19] Reichardt, W., Gompf, F., Aïn, M. & Wanklyn, B. M. Lattice dynamics of cupric oxide. *Zeitschrift für Physik B Condensed Matter* **81**, 19–24 (1990).

[20] Akopyan, A., Prasai, N., Rebello, A., Neumeier, J. J. & Cohn, J. L. Anisotropic thermal conductivity of cuo. *Physical Review Materials* **8**, 124403 (2024).

[21] Zheng, X.-G. *et al.* Evidence of charge stripes, charge-spin-orbital coupling and phase transition in a simple copper oxide cuo. *Journal of the Physical Society of Japan* **70**, 1054–1063 (2001).

[22] Wu, D., Zhang, Q. & Tao, M. Lsda+u study of cupric oxide: Electronic structure and native point defects. *Physical Review B* **73**, 235206 (2006).

[23] Momma, K. & Izumi, F. Vesta 3 for three-dimensional visualization of crystal, volumetric and morphology data. *Journal of Applied Crystallography* **44**, 1272–1276 (2011).
