# Supplementary material for: Hierarchical quasiparticle dynamics in antiferromagnets revealed by time- and momentum-resolved X-ray scattering
Source: Nat Commun. 2026 Sep 29;17:11222. doi: 10.1038/s41467-026-77964-5 (PMC13720009; doi:10.1038/s41467-026-77964-5)
Supplement: Supplementary file 2 [file 41467_2026_77964_MOESM2_ESM.pdf]

nature portfolio

Peer Review File

Hierarchical quasiparticle dynamics in antiferromagnets revealed by time- and momentum-resolved X-ray scattering

Corresponding Author: Dr Hiroki Ueda

This file contains all reviewer reports in order by version, followed by all author rebuttals in order by version.

Version 0:

Reviewer comments:

Reviewer #1

(Remarks to the Author)
In this manuscript, Ueda et al., are investigating the enery flow among different subsystems in the antiferromagnet CuO following the momnentum-resolved quasi-particle dynamics of magnons and phonons with femtosecond temporal resolution. They find a hierachy in how above-bandgap excitation leads to quasi-instantaneous disorder of the spin system which then couples to thermalized magnons and eventually recover via scattering to phonons. With the help of quantum-kinetic simulations and density-functional theory, the authors further identify the dispersion mismatch of magnons and phonons as the bottleneck determining the lifetime of the quasiparticles.

In my opinion, the work is very appealing as it combines theory with essentially three state-of-the-art time-resolved X-ray experiments on one and the same sample. In that sense, the auhtors have really taken great efforts to add a new perspective on how energy is distributed after laser-excitation. Especially the resonant diffuse scattering with magnons is new and can be indeed "transformative". The manuscript is also very well written, the data is of high quality and is further well presented in the figures.

However, I would like the authors to consider my following questions and comments.

1. The abstract claims, that the authors found a very "general" framework how energy is distributed. But in the introduction and later in the discussion, this generallity is narrowed down to AFM insulators. Please adjust the abstract accordingly.
2. The introduction teasers, that the "transient behavior of the electronic, magnetic, and lattice systems" is investigated. While I fully agree on the last two items, I do not see a direct experimental observable of the electronic degree of freedom (excluding spin). All discussuion about electronic dynamics is drawn indirectly from the RXD signal, literature & theory.
3. The points of measurement in recirporcal space for RDS and RXD must of course be different, but this difference seems not to be very consistent across the different datasets presented here. I suggest the authors to highlight, which miller indices are maybe changed on purpose and which changes or important or irrelevant.
4. Fig 2e: Why is the spectral behaviour of the RDS and the RXD signal so different? Are these extinctions effects in the RXD?
"without intensity reduction due to the diffraction tail (orange)." - is this what you mean here? what is meant by "diffraction tail"?
5. How is the pump-fluence exactly defined?
6. I could not find any explanation for the non-linearity of the fluence dependence (Fig 2f) for both signals.
7. Fig. 2g: the change of the difference RDS signal with polarization is not linear around 0. Why is that, and is that not a hint towards non-magnetic contributions?
8. SI Note 5: How is the temperature of the "hot magnons" estimated as 500K? I could not find anything about that in the SI note.
9. Can the authors support their statment about a constant width of the AFM Bragg peak also with a plot e.g. in the SI?
10. Please convert the detector axis in Figs S7a and S10a,b into reciprocal space or at least add the hkl direction into S7a.
11. The order of the SI notes is a bit hard to follow from the main text. Why not order them how they are referenced by in the main text?
12. Regarding the separtation of RDS and RXD signals: how can you be sure, that at certain q, the upterm is not due to redistribution of magnon population but simply by recovery? In my opinion, for difference dignals < 0 this is ambigious.
13. caption Fig 3: amplitudes (a) must be captial A

14. Fig 3a: The colorcode code could be improved to better separate the q values especially for the open symbols. Maybe one should also think of having a split axis for the positive differences asin the colorbars. Are there actually errorbars included, as the legend suggests?
15. The authors claim, that the RDS signal is symmetric around 0.5 0 -0.5 peak, but the data in 3a is clearly not laying on each other for open and filled symbols - isn't that a controversy?
16. The colorplots in Fig 3b, c,e seem to have some kind of interpolation between data points applied. If so, I strongly suggest to show only the real data point (and maybe add some interpolated contours on top for visibility)
17. As all measurements are highly momentum dependent, what is the actual resolution in q from the FEL and the detectors?
18. While the NRDS certainly addes value to this study - it was done with ps instead of fs pumping. I suggest that especially the ultrafast energy distribution between electrons, magnons and phonons is very different in this case - please comment on that.
19. While the new method of RDS with magnons is only one part of this work, the authors might add a short comparision/discussion to other alternatives than RIXS, e.g. EELS or Mie-enhanced BLS, which have been very sucessful lately in probing short wavelength magnons.

Reviewer #2

(Remarks to the Author)
I read “Hierarchical quasiparticle dynamics in antiferromagnets revealed by time- and momentum-resolved X-ray scattering” by Arnau Romaguera and colleagues. The work performs an ultrafast resonant scattering experiment on CuO to probe the dynamics of magnons in a momentum resolved way. Ultrafast photoexcitation quickly generates spin disorder that evolves back to equilibrium through a complex process of quasi-thermalization of the spins followed by magnon-phonon interactions. They are able to follow these processes thanks to the sensitivity of resonant scattering to magnetic degrees of freedom (in CuO) and the sensitivity of non-resonant scattering primarily to phonons. The experiment and theory are novel and extremely sophisticated. However, before I can recommend this work for publication I ask the authors to consider and address the following point.

The fact that the recovery dynamics are slow are unlikely to be something specific about the magnon-phonon interaction since recovery of long-range-order is observed to be universally slow in all spin- or charge-ordered systems driven out of equilibrium. This is a general behavior related to the creation of topological defects and is independent of the specifics of the system.

Driving a system with long range order out of equilibrium results in a high density of topological defects, this is the well-known Kibble-Zurek mechanism. The return to equilibrium is governed by the annihilation of these defects, which is slow. Under very general conditions the dynamics of equilibration exhibit self-similarity in the coarsening process towards long-range order and manifests as power laws in the structure factor S(k, t). The exponents depend only on the symmetry of the order parameter and the dimension. Do the authors observe self-similarity in S(k,t) as expected from theory (see see Eq. (1) and Fig. 2b in Li PRB 2023)? I believe the paper will benefit substantially from a discussion of the structure factor S(k, t) in Fig. 3b in the context of topological defects and their role on equilibration as predicted from general arguments (see the literature cited blow.) This broadens the context of the current study because this is the generic expectation of the dynamics of a broken-symmetry phase transition and while there are multiple theoretical works there is little experimental data (certainly not for spins as far as I know). Generally, topological defects are expected far from equilibrium, if this is not the case here or if there are discrepancies with predictions it should be discussed at least briefly.

Some references on universality and self-similarity of the structure factor:

Rodriguez-Nieva Phys Rev B 104, L060302 (2021) (theory Heisenberg model)
Li et al, Phys Rev B 108, 144303 (2023) (theory antiferromagnets)
Mitrano et al, Sci. Adv. 5, eaax3346 (2019); Orenstein et al, Phy Rev X 15, 031058 (2025) (charge order)
Huh et al Nat Phys 20, 402–408 (2024) (cold gasses)

Version 1:

Reviewer comments:

Reviewer #1

(Remarks to the Author)
The authors gave very satisfactory answers to all of my questions and comments.
In my view, the paper can now be published as is in Nature Communications.

Reviewer #2

(Remarks to the Author)

I have read the response and the modified manuscript. The authors have implemented my suggestions very thoroughly. I support publication of this work.

Open Access This Peer Review File is licensed under a Creative Commons Attribution 4.0 International License, which permits use, sharing, adaptation, distribution and reproduction in any medium or format, as long as you give appropriate credit to the original author(s) and the source, provide a link to the Creative Commons license, and indicate if changes were made.
In cases where reviewers are anonymous, credit should be given to 'Anonymous Referee' and the source.
The images or other third party material in this Peer Review File are included in the article's Creative Commons license, unless indicated otherwise in a credit line to the material. If material is not included in the article's Creative Commons license and your intended use is not permitted by statutory regulation or exceeds the permitted use, you will need to obtain permission directly from the copyright holder.
To view a copy of this license, visit https://creativecommons.org/licenses/by/4.0/

Reviewer feedback

Below, we provide a point-by-point response to all comments and indicate where corresponding revisions have been made in the manuscript and supplementary information in **red**, with our response in **black** and the referee comments in **blue**.

Reviewer 1

We greatly appreciate the Reviewer's positive assessment of our work as very appealing and of the resonant diffuse scattering with magnons as new and transformative. The Reviewer requested addressing the following questions and comments for clarity and consistency.

1. The abstract claims, that the authors found a very "general" framework how energy is distributed. But in the introduction and later in the discussion, this generality is narrowed down to AFM insulators. Please adjust the abstract accordingly.

   In response to this comment, we changed the last sentence of the abstract as follows.
   "This microscopic framework transcends phenomenological models and generalizes across antiferromagnetic insulators, establishing design principles for ultrafast control of antiferromagnetic order."

2. The introduction teasers, that the "transient behavior of the electronic, magnetic, and lattice systems" is investigated. While I fully agree on the last two items, I do not see a direct experimental observable of the electronic degree of freedom (excluding spin). All discussuion about electronic dynamics is drawn indirectly from the RXD signal, literature & theory.

   As the reviewer noted, we have no direct experimental data on electronic dynamics, only theoretical data. To avoid confusion, we removed "electronic" from the sentence.

3. The points of measurement in recirporcal space for RDS and RXD must of course be different, but this difference seems not to be very consistent across the different datasets presented here. I suggest the authors to highlight, which miller indices are maybe changed on purpose and which changes or important or irrelevant.

   Our experimental setup allows simultaneous collection of RXD and RDS signals when RDS signals are measured at the momentum (0.49, 0.01, -0.51) (Fig. 2**d** and 2**g**), since the two diodes are mounted on a single tower, as described in the Supplementary Information. For some measurements, such as the fixed-momentum incident X-ray energy dependence, we chose a different momentum (0.5, -0.08, -0.5) to suppress the RXD tail contribution. We clarify this in the caption of Fig. 2 of the revised manuscript.
   "RDS intensity was collected at (0.49, 0.01, -0.51), which is the momentum allowing simultaneous collection with RXD intensity, unless otherwise stated."

4. Fig 2e: Why is the spectral behaviour of the RDS and the RXD signal so different? Are these extinctions effects in the RXD?
   "without intensity reduction due to the diffraction tail (orange)." - is this what you mean here? what is meant by "diffraction tail"?

The dip structure in the energy dependence of the RXD signals arises from self-absorption, as explained in Ref. 26. A substantial fraction of diffracted photons is reabsorbed while propagating through the crystal under strong resonance, producing a dip near the absorption peak energy. This effect is significant only over long propagation distances. RDS signals, by contrast, are dominated by the region near the surface owing to the finite penetration depth of the pump, and are therefore much less affected by self-absorption. We added the following clarifying sentence.
"Note that the spectral difference from RXD signals exhibiting a dip structure due to strong self-absorption [26] likely originates from a larger magnon density, as discussed later, near the surface with a depth profile, limiting the self-absorption effect."

Regarding the "diffraction tail", the RXD profile has a finite width in reciprocal space. Even when the employed momentum is only close to but not exactly at the Bragg point, a small residual RXD contribution still appears in the resonant scattering intensities. This is what we refer to as the diffraction tail. It is removed by fitting the time traces, as described in the manuscript and shown in Figs. 3**a**-**f**.

5. How is the pump-fluence exactly defined?

The pump fluence f was defined as $f = p/\pi r^2$, where p is the pulse energy and r is the beam waist measured at $1/e^2$. We added this definition to the Method section.
"Laser fluence f is defined as $f = p/\pi r^2$, where p and r are the pulse energy and the beam waist measured at $1/e^2$, respectively."

6. I could not find any explanation for the non-linearity of the fluence dependence (Fig 2f) for both signals.

The non-linearity arises from proximity to the magnetic phase transition temperature. The RXD signal shows strong nonlinearity consistent with the known temperature dependence of the AFM Bragg reflection intensity (Ref. 25). Because the RDS signals were collected near the AFM Bragg peak, its tail also contributes to the nominal "RDS" signals (see also our reply to Comment 4). The strong suppression of the AFM Bragg peak near the transition therefore imprints non-linearity onto the "RDS" signals as well. We modified the following sentence accordingly.
"The upturn signal decreases at high fluence because the RXD intensity reduction is larger and nonlinear at temperatures closer to the AFM transition temperature (Supplementary Note 5 for temperature estimation)."

7. Fig. 2g: the change of the difference RDS signal with polarization is not linear around 0. Why is that, and is that not a hint towards non-magnetic contributions?

The non-monotonic behavior in Fig. 2**g** stems from the RXD tail contribution (see also our reply to Comment 4). We clarify this in the caption of Fig. 2.
"The fast intensity reduction observed in **d** and **g** is due to the RXD tail contribution (see main text)."

8. SI Note 5: How is the temperature of the "hot magnons" estimated as 500K? I could not find anything about that in the SI note.

The hot magnon temperature was estimated from the simulated magnon density assuming a Boltzmann distribution. We clarify this in the revised supplementary information.
"Hot-magnon temperatures at fast timescales, estimated from the simulated magnon density assuming a Boltzmann distribution, reach ~500 K at (0.5, 0.1, -0.5), far exceeding the bulk quasi-equilibrium limit of ~210 K."

9. Can the authors support their statment about a constant width of the AFM Bragg peak also with a plot e.g. in the SI?

We added rocking curves taken at a delay time of ~4.7 ps, with and without optical excitation, as shown in Fig. S4 of the revised Supplementary Information. The two curves show similar peak widths, supporting our statement that the width of the AFM Bragg peak is constant. Note that, as described in the Supplementary Information, the measurements on the AFM Bragg peak were performed with the photodiode that lacks sufficient sensitivity to detect RDS signals. We refer to this figure at the relevant point in the revised manuscript, and added the following sentences to Supplementary Note 2.
"The constant width (0.6°) of rocking curve scans with and without the optical excitation, shown in Fig. S4, confirms the constant correlation length of the AFM order. This observation indicates that we do not melt the AFM order into the disordered phase but rather reduce the amplitude of the AFM order parameter."

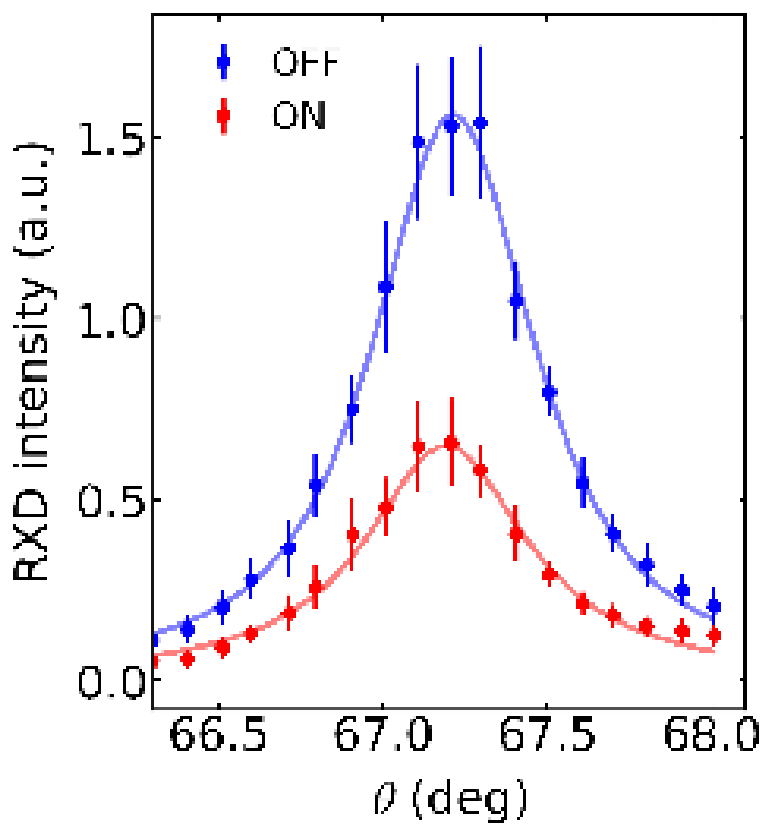

Fig. S4 | Rocking curve with (red) and without (blue) the optical excitation, measured at a delay time of ~4.7 ps.

10. Please convert the detector axis in Figs S7a and S10a,b into reciprocal space or at least add the hkl direction into S7a.

As suggested, we added the hkl direction to Fig. S7**a** in the revised Supplementary Information.

11. The order of the SI notes is a bit hard to follow from the main text. Why not order them how they are referenced by in the main text?

    As suggested, we reordered the supplementary notes to follow their order of citation in the main text.

12. Regarding the separtation of RDS and RXD signals: how can you be sure, that at certain q, the upterm is not due to redistribution of magnon population but simply by recovery? In my opinion, for difference dignals < 0 this is ambiguous.

    We understand the Reviewer's question as asking whether the upturn signal could reflect simple recovery of the magnetic order rather than redistribution of magnon population in reciprocal space, which is our interpretation.
    Long-range magnetic recovery is tracked by the RXD intensity, which stays at a reduced, roughly constant level for some time after the pump, whereas the RDS intensity evolves clearly as a function of momentum. This momentum dependence is therefore not attributable to simple recovery.
    Near the Γ point, RDS signals show an initial drop from the RXD tail contribution (see our reply to Comment 4). Fitting the data with two exponential functions, as described in the main text, successfully isolates the RXD and RDS contributions, and justifies our discussion of magnon redistribution regardless of whether the raw RDS data points are positive or negative.

13. caption Fig 3: amplitudes (a) must be captial A

    We thank the Reviewer for pointing this out. We fixed it in the revised manuscript.

14. Fig 3a: The color code could be improved to better separate the q values especially for the open symbols. Maybe one should also think of having a split axis for the positive differences as in the colorbars. Are there actually errorbars included, as the legend suggests?

    We agree the original panel could be clearer. In the revised manuscript, we added an inset in Fig. 3**a** to better show the small upturn behavior. We added the following sentence to the caption of Fig. 3.
    "The inset shows a zoom-in of the upturn signals."

15. The authors claim, that the RDS signal is symmetric around 0.5 0 -0.5 peak, but the data in 3a is clearly not laying on each other for open and filled symbols - isn't that a controversy?

    In the revised main text, Figs. 3**d** (RXD) and 3**f** (RDS) include Gaussian fits to the momentum dependence of amplitude and time constant. These fits demonstrate that the RDS signals are nearly symmetric. The amplitude of the upturn changes rapidly near the Γ point, so even a small momentum offset produces a relatively large apparent intensity deviation between positive and negative *q* data. We find that this behavior is well accounted for by a small error in the definition of the momentum origin. We added the following sentence to the caption of Fig. 3.

"The slight deviation from the symmetric behavior, represented by systematically larger signals in the negative q range than the positive q range, is due to a small error in the definition of the origin ($q = 0$)."
and
", as shown by the green, blue, and orange solid curves representing the Gaussian-dominated pseudo-Voigt fits (guides to the eye)."

16. The colorplots in Fig 3b, c,e seem to have some kind of interpolation between data points applied. If so, I strongly suggest to show only the real data point (and maybe add some interpolated contours on top for visibility)

    Following the reviewer's comment, we modified Figs. 3**b**, 3**c**, and 3**e** to show the real data points overlaid by interpolated contour plots.

17. As all measurements are highly momentum dependent, what is the actual resolution in q from the FEL and the detectors?

    We thank the Reviewer for raising this important technical point. The beamline energy resolution was ~500 meV, giving a corresponding q broadening on the order of 10^{-4} r.l.u. The angular acceptance of the avalanche photodiode was ~2.25°, corresponding to a q broadening of ~5×10^{-3} r.l.u. We added the following sentence to the Method section.
    "Our momentum resolution in tr-RDS measurements is estimated as ~5×10^{-3} r.l.u. from the beamline energy resolution (~500 meV) and the angular acceptance of the avalanche photodiode (~2.25°)."

18. While the NRDS certainly addes value to this study - it was done with ps instead of fs pumping. I suggest that especially the ultrafast energy distribution between electrons, magnons and phonons is very different in this case - please comment on that.

    The Reviewer is correct that pump pulse durations differed between the RXD/RDS (~35 fs) and NRDS (~1 ps) experiments. This could, in principle, alter the ultrafast dynamics, since the picosecond pulse continues to drive the electronic system while energy dissipates into magnons. However, we expect this effect to be minor, at most a slight smearing of the magnon creation process, because the typical doublon lifetime (~200 fs) exceeds the RXD/RDS pump pulse duration. We therefore expect the resulting states after the pulse to be very similar in both cases. We added the following sentence to the Methods section.
    "The pump pulse duration in tr-NRDS measurements is longer than in tr-RDS measurements, leading to continuous electronic excitation during the energy dissipation into magnons. However, this effect is likely not significant for the resulting states after optical excitation, because the typical doublon lifetime exceeds the pulse duration in tr-RDS measurements and limits the energy transfer process regardless of pump pulse duration."

19. While the new method of RDS with magnons is only one part of this work, the authors might add a short comparision/discussion to other alternatives than RIXS, e.g. EELS or Mie-enhanced BLS, which have been very sucessful lately in probing short wavelength magnons.

We thank the Reviewer for this constructive comment, which helps situate tr-RDS relative to existing techniques.
EELS has substantially lower sensitivity to magnons than to phonons (newly added Ref. 52), so separating the two requires careful background subtraction, and even then, magnon and phonon contributions remain difficult to disentangle in the energy-momentum range where they overlap. This limits access to low-energy magnons that RDS can resolve directly. The small cross-section also makes data acquisition slow, rendering time-dependent momentum evolution, as demonstrated here with tr-RDS, extremely difficult to access with EELS. EELS additionally requires thin-flake samples to transmit electron beams, adding a constraint on sample fabrication.
Mie-enhanced Brillouin light scattering accesses the low-energy range but only within a limited momentum range. It also requires Si nanostrips on the magnetic material to generate the Mie resonance (newly added Ref. 53), which spatially modulates the optical excitation and complicates time-resolved measurement of the material's intrinsic response under uniform excitation.
We added the following sentences on p. 9 in the main text.
"Tr-RDS measurements capture the ultrafast dynamics of magnons, which most conventional and newly developed static techniques cannot access because of fundamental limitations in pulse duration and inefficient cross-sections, e.g., inelastic neutron scattering, electron energy-loss spectroscopy [52], and Mie-enhanced Brillouin light scattering [53]."

Reviewer 2

We sincerely appreciate the Reviewer's positive assessment of our work as novel and extremely sophisticated from both experimental and theoretical perspectives. The Reviewer requested that we consider the following point before recommending our work for publication.

1. The fact that the recovery dynamics are slow are unlikely to be something specific about the magnon-phonon interaction since recovery of long-range-order is observed to be universally slow in all spin- or charge-ordered systems driven out of equilibrium. This is a general behavior related to the creation of topological defects and is independent of the specifics of the system.

   Driving a system with long range order out of equilibrium results in a high density of topological defects, this is the well-known Kibble-Zurek mechanism. The return to equilibrium is governed by the annihilation of these defects, which is slow. Under very general conditions the dynamics of equilibration exhibit self-similarity in the coarsening process towards long-range order and manifests as power laws in the structure factor S(k, t). The exponents depend only on the symmetry of the order parameter and the dimension. Do the authors observe self-similarity in S(k,t) as expected from theory (see see Eq. (1) and Fig. 2b in Li PRB 2023)? I believe the paper will benefit substantially from a discussion of the structure factor S(k, t) in Fig. 3b in the context of topological defects and their role on equilibration as predicted from general arguments (see the literature cited blow.) This broadens the context of the current study because this is the generic expectation of the dynamics of a broken-symmetry phase transition and while there are multiple theoretical works there is little experimental data (certainly not for spins as far as I know). Generally, topological defects are expected far from equilibrium, if this is not the case here or if there are discrepancies with predictions it should be discussed at least briefly.

   We thank the Reviewer for this insightful comment, which we address in two parts: (1) the possible applicability of the Kibble-Zurek mechanism, and (2) self-similarity in the resonant diffuse scattering cross-section.

   (1) The Kibble-Zurek mechanism applies when an ordered state is collapsed by excitation and then recovers through the nucleation and growth (coarsening) of small domains. This coarsening process is intrinsically slow, as the Reviewer notes.
   We believe our excitation lies in a different regime, where Kibble-Zurek physics and subsequent coarsening do not apply, because the photoexcitation does not collapse the antiferromagnetic order parameter into a disordered or incommensurate state but only reduces its amplitude. No domain nucleation or coarsening is therefore required for recovery. We tested this directly via rocking curve measurements, which probe the correlation length and thereby any coarsening. Figure S4 shows rocking curves at a delay time of ~4.7 ps with and without optical excitation (see also our reply to Comment 9 of Reviewer 1). The peak width remains constant within error bars, indicating no apparent coarsening. Additionally, while the RDS intensities evolve significantly with momentum, the reduced RXD intensities remain nearly constant, further indicating that the dynamics probed by tr-RDS are not tied to coarsening.

We further complement this view by following the analysis procedure of newly added Ref. [34] [G. Orenstein *et al.*, *Phys. Rev. X* **15**, 031058 (2025)], raised by the Reviewer. Figures R1**a**, R1**b**, and R1**c** display dynamical scaling of our resonant diffuse scattering cross-section shown in Fig. 3**e**, and correspond to Figs. 2(a), 2(b), and 2(c) of that reference. The obtained growth exponent β = 0.07 is substantially smaller than the value of 0.29 reported in the reference.

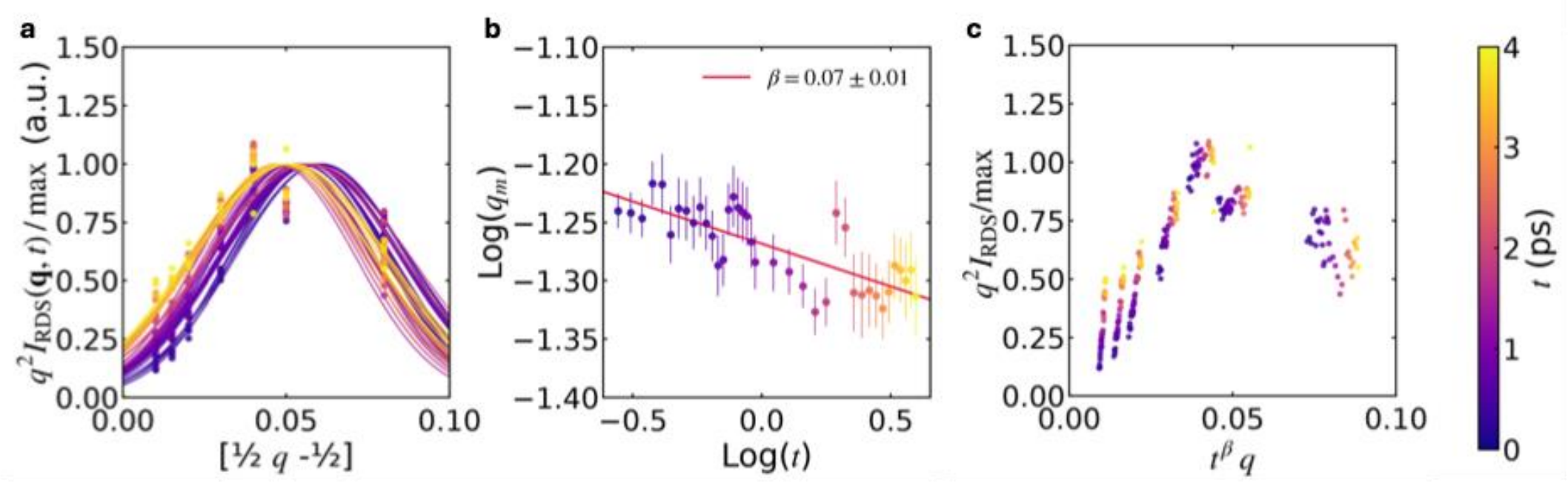

Fig. R1 Dynamical scaling of tr-RDS signals. **a**, $q^2 I_{RDS}$ plotted at different delays *t*. Solid curves are fits to a Gaussian peak, giving the peak momentum position, q_m. The vertical axis is normalized to the maximum value at each delay. **b**, log(*t*) versus log(q_m). The red straight line is a fit to the data with β = 0.07, where q_m is proportional to $t^{-\beta}$. **c**, Rescaled plot of $q^2 I_{RDS}$ as a function of $t^\beta q$, where β = 0.07.

We added the following sentences to the revised manuscript.

"Note that our excitation does not quench the AFM order parameter, as indicated by the constant correlation length after excitation. Therefore, the recovery process is not described by the Kibble-Zurek mechanism and subsequent coarsening of small domains [34]."

(2) The observed self-similarity may imply an inverse particle cascade in wave turbulence, i.e., a kinematic description of transport in momentum space in which particles of conserved number flow toward lower momentum. In our microscopic simulation, the number of magnons is indeed the conserved quantity, and the flow towards lower momentum is consistent with our microscopic view of the dynamics. Newly added Ref. [43] [Z. Li *et al.*, *Phys. Rev. B* **108**, 144303 (2023)], raised by the Reviewer, reports power-law scaling in this cascade process. While that reference considered a two-dimensional Heisenberg system, our system is described by a three-dimensional effective Heisenberg Hamiltonian, which may lead to deviations from the nominal scaling exponents α (= 1) and β (= 0.5), as well as the power-law exponent ν (= 2.33), of the model system.

Figure S10**b** shows the rescaled spin-spin correlation function using the nominal scaling exponents α and β for our data, corresponding to Fig. 2(b) of Ref. [18]. The extracted power-law exponent is 2.28±0.08, reasonably close to the nominal value of 2.33 derived from the model. Particle non-conserving processes, such as the anomalous magnon-phonon scattering discussed in the main text, will eventually become relevant at later times. Differences in dimensionality, detailed magnetic interactions in the Hamiltonian, possible violations of SU(2) symmetry, and a finite pump pulse duration and doublon lifetime, compared to Ref. [18], may together account for the small remaining discrepancy

in the small $t^\beta q$ range. Nevertheless, we believe that our result captures the essential physics and implies the occurrence of an inverse particle cascade in wave turbulence, consistent with our microscopic theory.

As the reviewer pointed out, this result may represent the first experimental data of far-from-equilibrium universality in spin dynamics in condensed matter. We are grateful to the Reviewer for raising this question; this discussion indeed adds substantial value to our manuscript, as the Reviewer anticipated.

We added the following sentences to the revised manuscript and made a new Supplementary Note.

"Our tr-RDS signals exhibit self-similarity, as shown in Fig. S10**a**. Far-from-equilibrium universality in antiferromagnets was theoretically discussed for general two-dimensional Heisenberg Hamiltonians [43]. By using the same scaling exponents as in Ref. [43], we obtained a power-law exponent of 2.28±0.08 (Fig. S10**b**), reasonably close to the nominal value (2.33) for an inverse particle cascade, in which a conserved number of particles flows to lower momentum in reciprocal space, consistent with and further validating our microscopic view of magnon dynamics (see also Supplementary Note 8). Differences in dimensionality and detailed Hamiltonian, and a possible non-conserving scattering process, as well as a finite pump pulse duration and doublon lifetime, may account for the small discrepancy in the small $t^\beta q$ range." to the main text.

"

Supplementary Note 8

Our microscopic understanding of magnon redistribution from the non-thermal photoexcited state via magnon-magnon scattering is equivalent to the inverse particle cascade, in which a conserved number of particles flows to lower momentum in reciprocal space, discussed in Ref. [18]. This theoretical study on two-dimensional Heisenberg antiferromagnets reported that a long-lived pre-thermal regime, in which an inverse particle cascade occurs, exhibits self-similarity in the fluctuations of the staggered magnetization

$$\langle M_{-k}(t)\, M_k(t)\rangle = t^\alpha f\left(t^\beta |\mathbf{q}|\right).$$

Here, α = 1.0, and β = 0.5. f is the universal scaling function, exhibiting $f(x) \sim 1/|x|^\nu$ over a broad momentum range, with ν = 2.33. The universality holds as long as the Heisenberg Hamiltonian preserves SU(2) symmetry. Our effective Hamiltonian is three-dimensional, but of Heisenberg type.

Figure S10**a** shows the momentum dependence of $q^2 I_{RDS}$, which exhibits a peak that fits well to a Gaussian and indicates self-similarity across different delays. Using the same spatial-temporal scaling exponents α and β as in Ref. [18], we rescaled our tr-RDS signals (original data shown in Fig. 3**e**), which measure the spin-spin correlation function (see Fig. S10**b**). The extracted power-law exponent is 2.28±0.08, close to the nominal value of 2.33 for the model in Ref. [18]. The small deviation may be accounted for by differences in dimensionality and detailed Hamiltonian, a possible non-conserving scattering process, a finite pump pulse duration, and a finite doublon lifetime. Nevertheless, the reasonable agreement supports our microscopic understanding of magnon-magnon scattering, redistributing magnon density in reciprocal space.

"

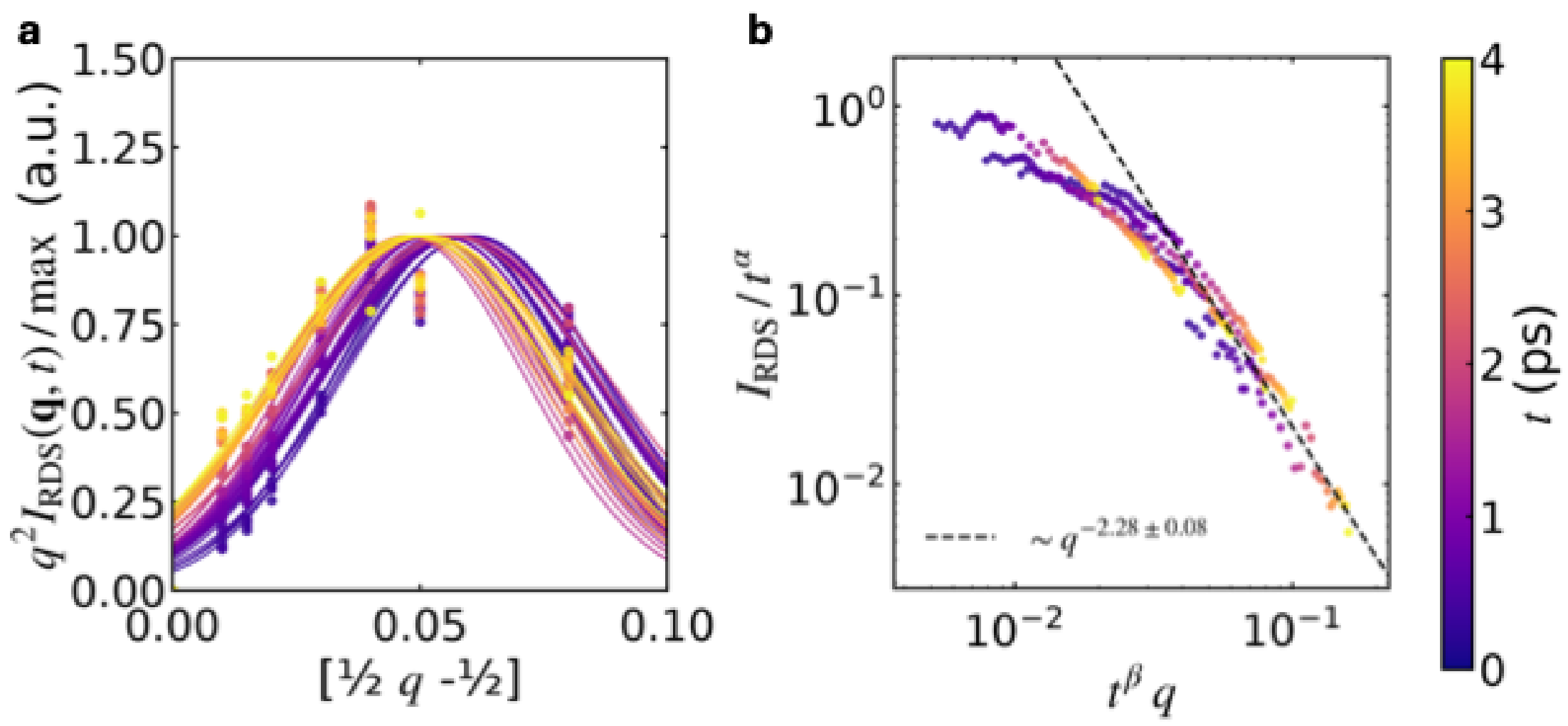

Fig. S10 | Self-similarity and power-law scaling of rescaled tr-RDS signals. **a**, $q^2 I_{RDS}$ plotted at different delays t. I_{RDS} signals are taken from Fig. 3**e**. Solid curves are fits to Gaussian peaks as guides to the eye, indicating self-similarities across different delays. The vertical axis is normalized to the maximum value for each delay. **b**, Rescaled tr-RDS signals with α = 1.0 and β = 0.5, following Ref. [18]. A linear fit yields a power-law exponent of 2.28±0.08, close to the nominal value of 2.33 for an inverse particle cascade in wave turbulence, in which particles flow toward lower momentum.
